# Simple synthesis of 2-(phenylsulphinyl)benzo[*d*]oxazole derivatives *via* a silver-catalysed tandem condensation reaction†

**DOI:** 10.1039/d5ra00983a

**Published:** 2025-02-19

**Authors:** Runsheng Xu, Qi Hu, Jiahao Hu, Guangqu Liu, Jin Xu

**Affiliations:** a College of Life and Health, Huzhou College Huzhou Zhejiang 211300 China xurunsheng@zjhzu.edu.cn xujin@zjhzu.edu.cn

## Abstract

Silver catalysed reactions have become an indispensable tool in organic synthesis due to their high efficiency, selectivity, and environmental friendliness. In this manuscript, the simple synthesis reaction generating 2-(phenylsulphinyl)benzo[*d*]oxazole derivatives *via* a silver-catalysed tandem condensation reaction is described. Starting from substituted 2-aminophenols or benzene-1,2-diamines, formaldehyde with substituted benzenethiols efficiently yields versatile biologically active 2-(phenylsulphinyl)benzo[*d*]oxazole derivatives and 2-(phenylsulphinyl)-1*H*-benzo[*d*]imidazole derivatives. These protocols were performed under mild reaction conditions, tested for wider substrate scope, and provide an economical approach for C(sp^2^)–sulphoxide bond formation.

## Abstract 2

Silver catalysed reactions have become an indispensable tool in organic synthesis due to their high efficiency, selectivity, and environmental friendliness.

## Graphical abstract

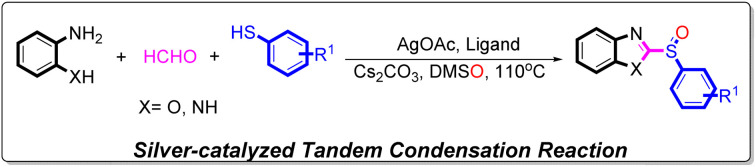

## 1. Introduction

**Scheme 1:**
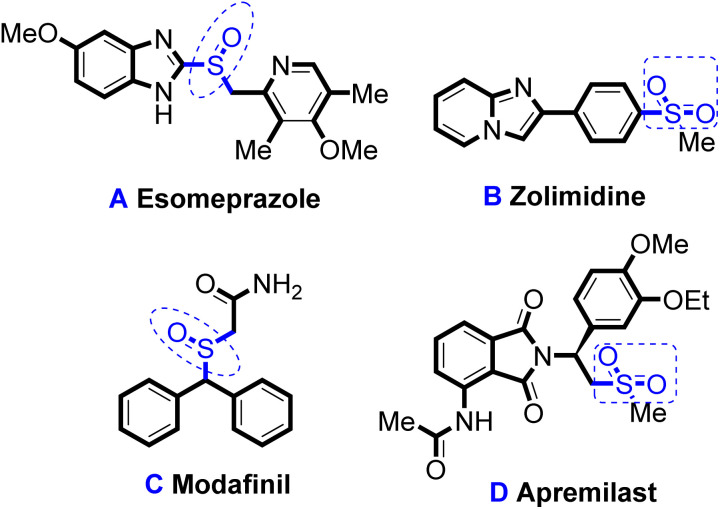
Important clinical drugs using organic sulphoxides and sulphone compounds.

Organic sulphoxides and sulphone compounds series have important applications in organic synthesis,^1–3^ medicines,^4,5^ and as functional materials.^6,7^ Many well-known general antibacterial agents utilising these materials have been commercialised and hold a significant place for application as pharmaceuticals worldwide (Scheme 1). Equally noteworthy is their significant market size. For example, esomeprazole (A) can effectively inhibit gastric acid secretion, and is the most widely used drug for treating diseases such as duodenal ulcer. Since its commercialisation in 1989, its global cumulative sales have exceeded 60 billion dollars.^8^ Zolimidine (B), an imidazole heterocyclic derivative drug, is mainly used for the treatment of digestive system disorders.^9^ Modafinil (C) is an *α*_1_ receptor agonist, used for the treatment of spontaneous hypersomnia and sleep disorders, and was first commercialised in the 1990s.^10^ Apremilast (D) is the first oral phosphodiesterase-selective inhibitor used to treat active and plaque psoriasis.^11^

The synthesis of organic sulphoxides and sulphone compounds has attracted extensive attention. Transition-metal catalysed cross-coupling reactions are the most frequently used methodology for the incorporation of a sulfur atom into aromatic frameworks.^12^ However, these methods commonly have significant limitations and shortcomings. For example, the starting aryl selenium reagents have to be synthesised and tailored to the substrate.^13^ Thus, it is of synthetic value to provide an efficient and concise pathway to access diverse unsymmetrical diaryl selenides. Methods of C(sp^2^)–sulphoxide bond formation have not been fully described previously.^14–18^

The introduction of organic sulphoxides and sulphone compounds into organic molecules *via* a transition-metal-catalysed reaction is an attractive and promising method for organic synthesis. In the reaction, organic sulphoxides compounds are synthesised *via* two cross-coupling partners. In recent years, significant progress with silver catalysed reactions have been made in the field of organic synthesis. Silver catalysed reactions have become an indispensable tool in organic synthesis due to their high efficiency, selectivity, and environmental friendliness. Our group has focused on traditional-metal catalysed C–H bond functionalisation.^19–25^ In this study, we describe a simple synthesis of 2-(phenylsulphinyl)benzo[*d*]oxazole derivatives *via* a silver-catalysed tandem condensation reaction. Starting from substituted 2-aminophenols or benzene-1,2-diamines and formaldehyde compounds, versatile and biologically active with substituted benzenethiols 2-(phenylsulphinyl)benzo[*d*]oxazole derivatives and 2-(phenylsulphinyl)-1*H*-benzo[*d*]imidazole derivatives can be efficiently synthesised. These protocols are performed under mild reaction conditions, allow wider substrate scope, and provide an economical approach toward C(sp^2^)–sulphoxide bond formation. Furthermore, the reaction mechanism was confirmed using control experiments.

## 2. Experimental methods and details

### 2.1 General procedures for preparation of 4, 7 and 9

A mixture of 2-aminophenol 1 (1.09 g, 10 mmol), formaldehyde 2 (0.45 g, 15 mmol) and benzenethiol 3a (2.43 g, 10 mmol), AgOAc (167 mg, 10 mol%), L4 (22 mg, 10 mol%), Cs_2_CO_3_ (6.52 g, 2 equiv.), DMSO (15 mL). The tube was evacuated and refilled with N_2_ three times. The reaction is carried out under nitrogen protection. The reaction mixture was stirred at 110 °C for 24 h. After it was cooled, the reaction mixture was diluted with 20 mL of ethyl ether for 3 times. The filtrate was washed with water (3 × 15 mL). The organic phase was dried over Na_2_SO_4_, filtered, and concentrated under reduced pressure. And filtered through a pad of silica gel, followed by washing the pad of silica gel with the same solvent (20 mL). The residue was then purified by flash chromatography on silica gel to provide the corresponding product. The pure product 2-(phenylsulfinyl)benzo[*d*]oxazole (4a) was obtained 1.92 g, 79% yield.

## 3. Results and discussion

At the beginning of our investigation, we developed the model reaction using 2-aminophenol 1, formaldehyde 2 and benzenethiol 3a to study reaction conditions including the optimisation of catalysts, ligands, bases, and solvents. As shown in Table 1, silver salts were used as the catalysts (entries 1–4), no desired product was obtained when the reaction was conducted in the presence of Ag_2_O as the catalyst in DMSO (entry 1). AgOAc was the most efficient catalyst species in this reaction (entry 4). All available ligands were then evaluated including L1–L6 (entries 4–9), and L4 resulted to be the most efficient catalyst species for this transformation (entry 7). Notably, the yield of product 4a was increased by 12% when the catalyst was changed to AgNO_3_ (entry 2). Screening different bases for C(sp^2^)–sulphoxide bond formation, Cs_2_CO_3_ was a more suitable base than others such as NaOH, Na_2_CO_3_, or K_2_CO_3_ (entries 11–14). The experimental results indicated that the proper solvent was critical for this reaction. When the reactions were conducted in apolar solvent such as CH_3_CN, or weak coordination solvent DMF, trace product was detected (entries 14 and 15). In addition, replacing DMF with DMSO, produced a better yield, this control experiment suggested that DMSO was critical for successful transformation. Lower yields were obtained under reactions performed at 100 °C and 120 °C. Remarkably, no desired product was obtained under O_2_ atmosphere (entry 15), indicating that N_2_ was essential for the reaction. Finally, the optimal reaction conditions were determined to be AgOAc as the catalyst, L4 as the ligand, Cs_2_CO_3_ as the base, the ratio of 1 : 1.5 : 1 (1 : 2 : 3a), under N_2_, in 110 °C, preparation for 24 h.

**Table 1:** Optimisation of the reaction conditions^a^

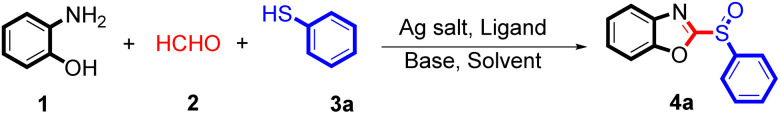					
Entry	Ligand	Ag salt	Base	Ratio 1 : 2 : 3a	Yield^b^ (%)
1	L1	Ag_2_O	Cs_2_CO_3_	1 : 1 : 1	0
2	L1	AgNO_3_	Cs_2_CO_3_	1 : 1 : 1	14
3	L1	AgBF_4_	Cs_2_CO_3_	1 : 1 : 1	17
4	L1	AgOAc	Cs_2_CO_3_	1 : 1 : 1	44
5	L2	AgOAc	Cs_2_CO_3_	1 : 1 : 1	31
6	L3	AgOAc	Cs_2_CO_3_	1 : 1 : 1	38
7	L4	AgOAc	Cs_2_CO_3_	1 : 1 : 1	73
8	L5	AgOAc	Cs_2_CO_3_	1 : 1 : 1	51
9	L6	AgOAc	Cs_2_CO_3_	1 : 1 : 1	38
10	L4	AgOAc	Cs_2_CO_3_	1 : 1.5 : 1	79
11	L4	AgOAc	NaOH	1 : 1.5 : 1	55
12	L4	AgOAc	Na_2_CO_3_	1 : 1.5 : 1	0
13	L4	AgOAc	K_2_CO_3_	1 : 1.5 : 1	44
14	L4	AgOAc	Cs_2_CO_3_	1 : 1.5 : 1	64^c^
15	L4	AgOAc	Cs_2_CO_3_	1 : 1.5 : 1	71^d^
16	L4	AgOAc	Cs_2_CO_3_	1 : 1.5 : 1	trace^e^
17	L4	AgOAc	Cs_2_CO_3_	1 : 1.5 : 1	trace^f^
18	L4	AgOAc	Cs_2_CO_3_	1 : 1.5 : 1	trace^g^
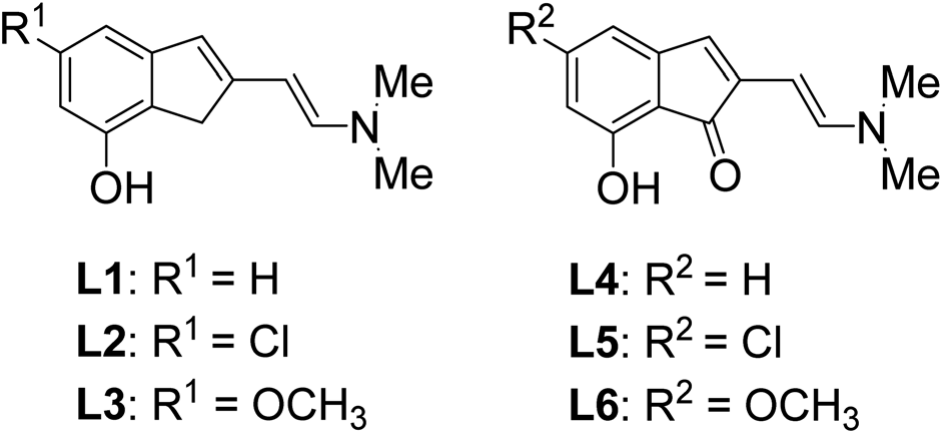					

a Unless otherwise noted, reactions conditions were 1 (10 mmol), 2 (10 mmol), 3a (10 mmol), Ag salt (10 mol%), ligand (10 mol%), base (2 equiv.), solvent (15 mL), 110 °C for 24 h, under N_2_.

b Isolated yield.

c 100 °C.

d 120 °C.

e In CH_3_CN.

f In DMF.

g Under O_2_.

The scope of aryl iodides was examined under optimal conditions. The results are shown in Table 2. 2-Aminophenol 1, formaldehyde 2, and a wide array of benzenethiol derivatives 3 were subjected to this reaction and generated products with good to excellent yields (59–92%). A variety of functional groups including methyl, methoxy, halogen, cyano, trifluoromethyl, and nitro groups were compatible with the benzenethiol derivatives 3. Both the electron-donating and electron-withdrawing benzenethiol derivatives 3 reacted smoothly with 2-aminophenol 1 and formaldehyde 2. Benzenethiol derivatives 3 bearing electron-donating groups showed better activity than electron-withdrawing groups. The free proton amine group may more strongly coordinate with the silver catalyst, which provided good yields (4g 85% yield, 4h 92% yield). Despite the electron-withdrawing effects of the trifluoromethyl group being so strong, the corresponding product 4p was still obtained at a 59% yield.

**Table 2:** Screening of aryl iodides in the silver-catalysed tandem condensation reaction^a^^,^^b^

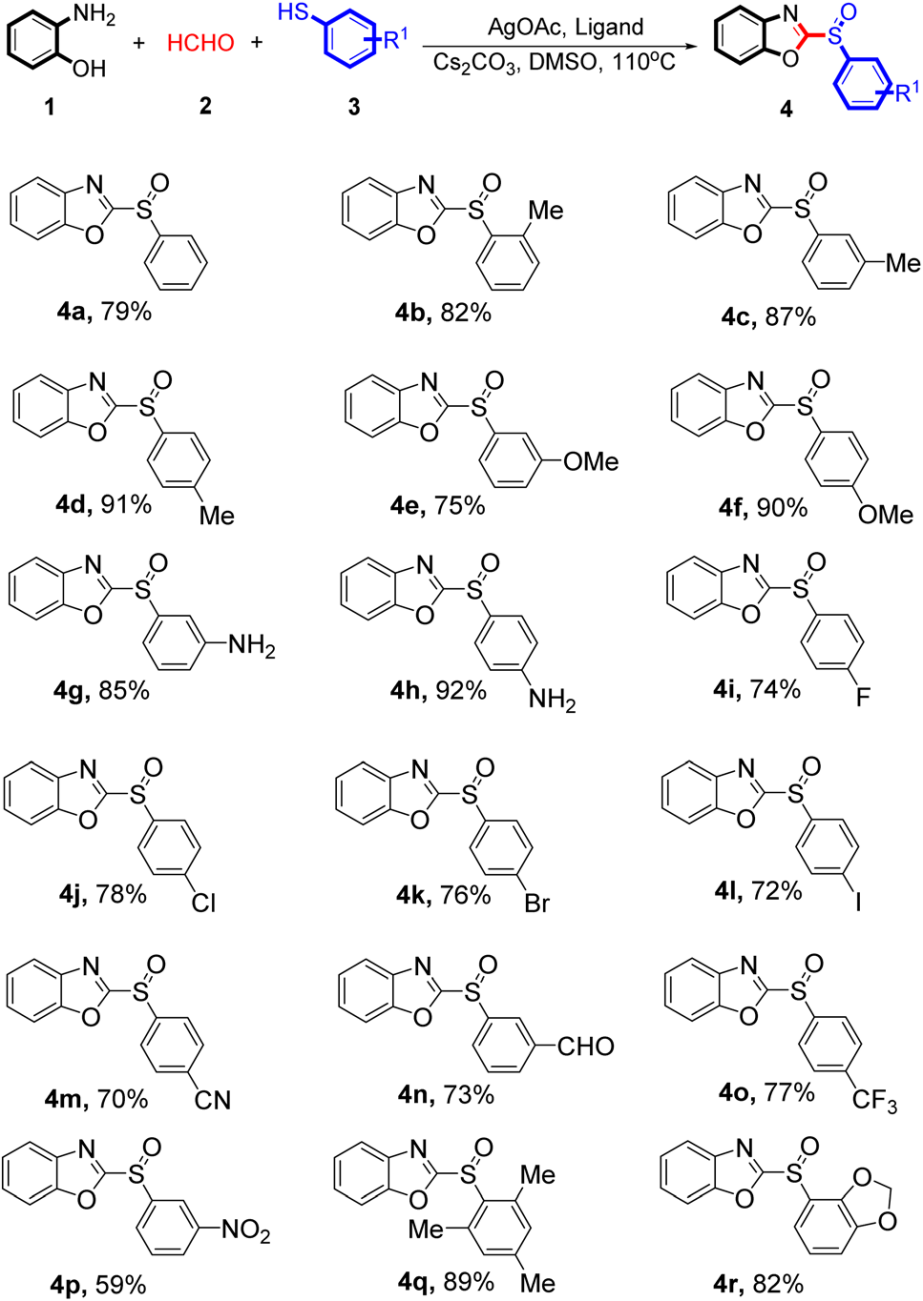

a Unless otherwise noted, reactions conditions were 1 (10 mmol), 2 (15 mmol), 3 (10 mmol), AgOAc (10 mol%), L4 (10 mol%), Cs_2_CO_3_ (2 equivalents), DMSO (15 mL), 110 °C for 24 h, under N_2_.

b Isolated yield.

Next, the reaction tolerance of benzenethiol derivatives 3 was evaluated and the diversity of 2-aminophenol derivatives 5 was further investigated under the optimised reaction conditions. A wide array of 2-aminophenol derivatives 5 were subjected to this reaction and the products were obtained in moderate to good yields (7979–86%). A variety of functional groups including methyl, methoxy, halogen, and amino groups were found to be compatible, as shown in Table 3. The free proton amine group may strongly coordinate with the silver catalyst, which attenuates the reactivity of transition-metal. We also evaluated strong electron withdrawing groups such as trifluoromethyl and nitro under the current reaction conditions, however, only achieved a decomposition of the starting material without any expected product.

**Table 3:** Silver-catalysed tandem condensation reaction using 2-aminophenol derivatives^a^^,^^b^

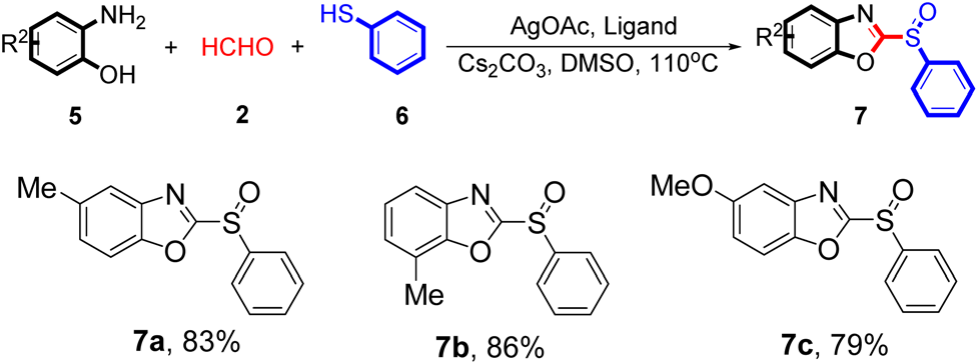

a Unless otherwise noted, reactions conditions were 5 (10 mmol), 2 (15 mmol), 6 (10 mmol), AgOAc (10 mol%), L4 (10 mol%), Cs_2_CO_3_ (2 equivalents), DMSO (15 mL), 110 °C for 24 h, under N_2_.

b Isolated yield.

Interestingly, the application scope of the reaction could be expanded to a wide array of benzene-1,2-diamine derivatives 8, generating products with good yields (75–88%, Table 4). Both electron-donating and electron-withdrawing benzene-1,2-diamine derivatives 8 reacted smoothly. Benzene-1,2-diamine derivatives 8 bearing electron-donating groups had better activity than derivatives bearing electron-withdrawing groups. Despite the electron-withdrawing effect of chlorinated substituent being so strong, the corresponding product 9c was still obtained at a yield of 81%.

**Table 4:** Silver-catalysed tandem condensation reaction^a^^,^^b^

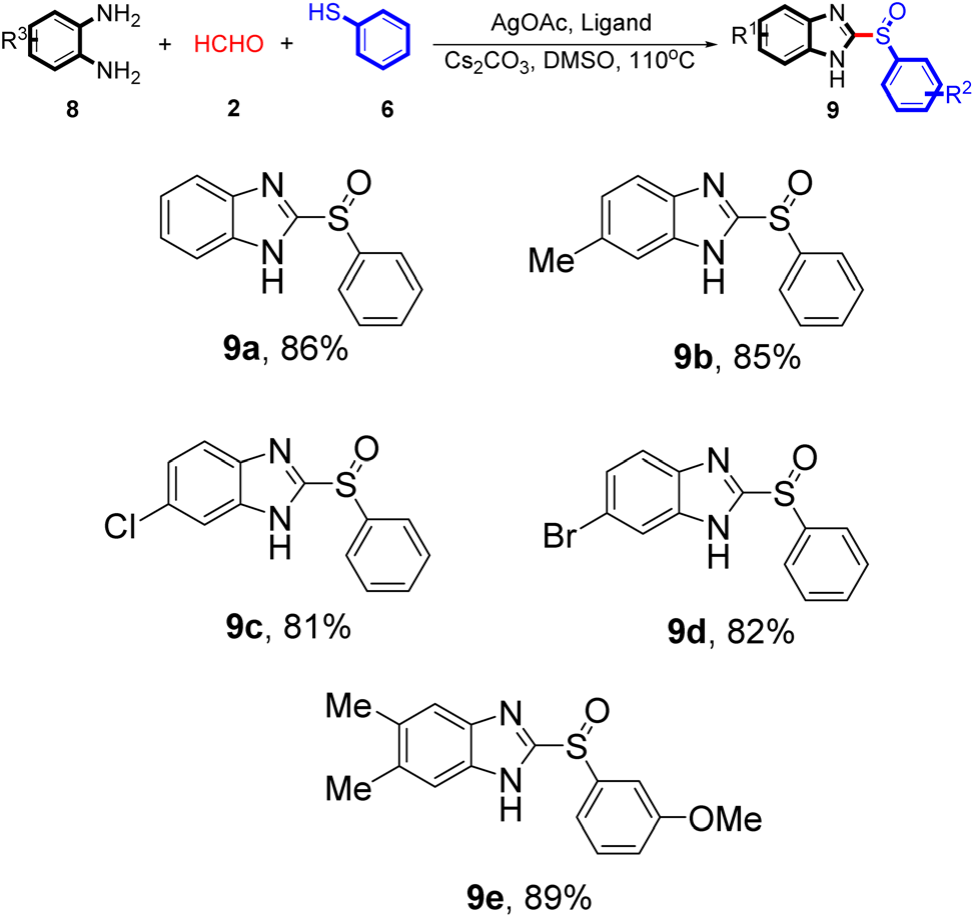

a Unless otherwise noted, reactions conditions were 8 (10 mmol), 2 (15 mmol), 6 (10 mmol), AgOAc (10 mol%), L4 (10 mol%), Cs_2_CO_3_ (2 equivalents), DMSO (15 mL), 110 °C for 24 h, under N_2_.

b Isolated yield.

**Scheme 2:**
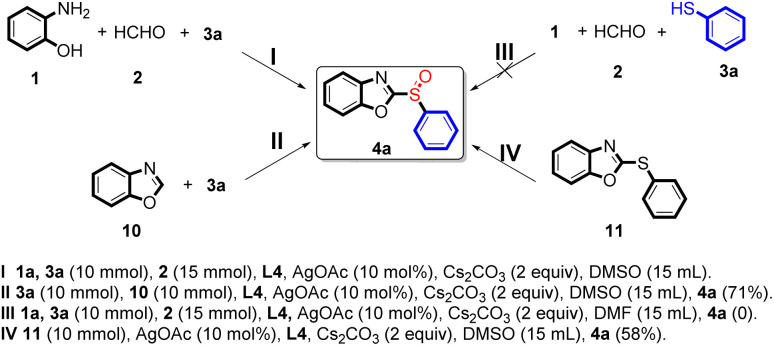
Silver-catalyzed tandem condensation reaction preliminary mechanism investigation.

Preliminary results using the reaction mechanism, were also obtained using additional reactions (Scheme 2). The model reaction (Scheme 2I) was tested with three other parallel reactions (Scheme 2II–IV). Benzo[*d*]oxazole 10 reacted with 3a promoted by hydrogen peroxide under the standard conditions and successfully obtained the target product 4a in 71% yield (Scheme 2II). Furthermore, the reaction with 11 performed under the standard conditions, successfully obtained the target product 4a at a yield of 58% (Scheme 2IV), which indicated that the reaction first undergoes a condensation reaction process. Furthermore, these results also indicated that DMSO was the necessary solvent for this reaction.

To obtain the preliminary results of the reaction mechanism, some additional reactions were been done (Scheme 2). At first, the model reaction (Scheme 2I) was conducted in other three parallel reactions (Scheme 2II–IV). However, results show that benzo[*d*]oxazole 10 reacted with 3a promoted by hydrogen peroxide under our standard conditions, successfully obtained the target product 4a in 71% yield (Scheme 2II). Forthermore, 11 reacted promoted under our standard conditions, successfully obtained the target product 4a in 58% yield (Scheme 2IV), which indicated that the reaction first undergoes a condensation reaction process. And those results also indicated that DMSO was the necessary solvent for this reaction.

**Scheme 3:**
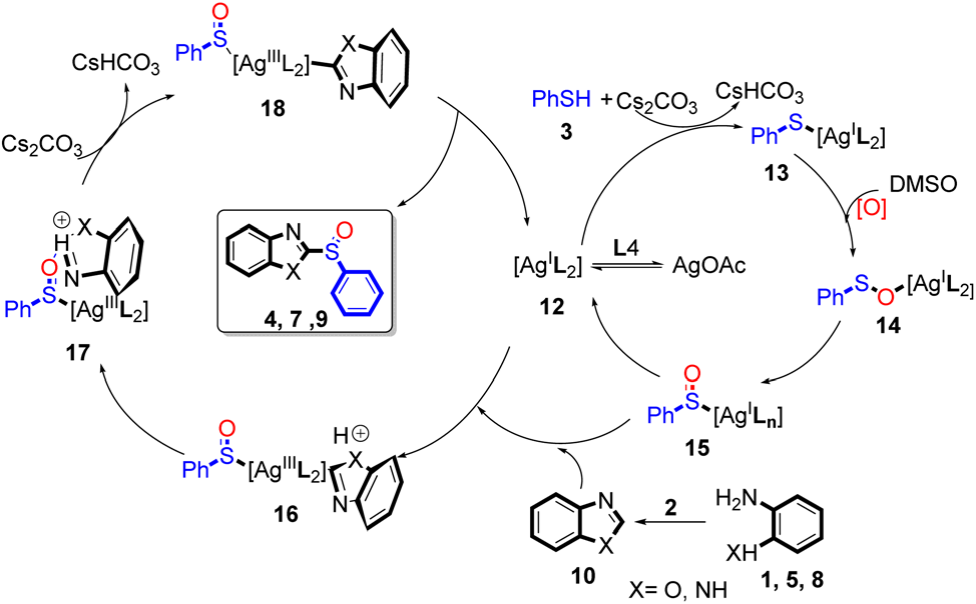
Proposed silver-catalysed tandem condensation reaction mechanism.

The above results suggested that the sulphoxidation products originated from thiophenol followed by the Ag-catalysed oxidation in the presence of DMSO.^26^ From these observations, we propose a possible mechanism (Scheme 3). At the beginning of the reaction, ligand coordination of the AgOAc and L4 generate complex 12. Next, oxidation allows the addition of 12, which is followed by a ligand exchange process with Cs_2_CO_3_ to give intermediate 13. The intermediate 13 is then transformed to intermediate 14 under DMSO by oxygen transfer.^27–29^ A silver *p*-benzyl intermediate 16 has been described previously,^30–36^ and has been used to develop useful synthetic intermediates. Intermediate 16 is used to produce 17*via* the silver *p*-benzyl coordination of generated Ag^III^ species, which further undergoes rapid oxidation to generate the keto functionality intermediate 18. Through the reductive elimination step, intermediate 18 generates the desired 2-(phenylsulphinyl)-6,7-dihydroquinoxaline derivatives, during which concomitantly complex 12 is formed, which re-enters the catalytic cycle.

## 4. Conclusions

Silver catalysed reactions have become an indispensable tool in organic synthesis due to their high efficiency, selectivity, and environmental friendliness. In this study, the development of a simple synthesis of 2-(phenylsulphinyl)benzo[*d*]oxazole derivatives *via* silver-catalysed tandem condensation reaction was described. Starting from substituted 2-aminophenols or benzene-1,2-diamines, and formaldehyde compounds with substituted benzenethiols, versatile biologically active 2-(phenylsulphinyl)benzo[*d*]oxazole derivatives and 2-(phenylsulphinyl)-1*H*-benzo[*d*]imidazole derivatives were efficiently synthesised. These protocols were performed under mild reaction conditions, and demonstrated wider substrate scope, and thus, provide an economical approach toward C(sp^2^)–sulphoxide bond formation. Furthermore, the reaction mechanism was confirmed using control experiments. Despite the great advancements in synthesis protocols, the capacity to incorporate substrates that allow the production of diverse 2-(phenylsulphinyl)benzo[*d*]oxazole derivatives and 2-(phenylsulphinyl)-1*H*-benzo[*d*]imidazole derivatives in a versatile way remains a significant challenge.

## Data availability

Data will be made available on request.

## Author contributions

Runsheng Xu conceptualization, supervision, project administration, writing – review & editing; Qi Hu: investigation, visualization, formal analysis; Jiahao Hu: investigation, formal analysis; Jin Xu: formal analysis, validation, supervision, project administration, writing – review & editing.

## Conflicts of interest

There are no conflicts to declare.

## Supplementary Material

RA-015-D5RA00983A-s001

## References

[cit1] Trenner J., Depken C., Weber T., Breder A. (2013). Angew. Chem., Int. Ed..

[cit2] Huang L. W., Xun X. D., Zhao M., Xue J. Z., Li G. F., Hong L. (2019). J. Org. Chem..

[cit3] Wei R. B., Xiong H. G., Ye C. Q., Li Y. J., Bao H. L. (2020). Org. Lett..

[cit4] Engman L., Stern D., Frisell H., Vessman K., Berglund M., Ek B., Andersson C.-M. (1995). Bioorg. Med. Chem..

[cit5] Wirth T. (2015). Angew. Chem., Int. Ed..

[cit6] Panda S., Panda A., Zade S. S. (2015). Coord. Chem. Rev..

[cit7] Somasundaram S., Chenthamarakshan C. R., de Tacconi N. R., Ming Y., Rajeshwar K. (2004). Chem. Mater..

[cit8] Jain K. S., Shah A. K., Bariwal J., Shelke S. M., Kale A. P., Jagtap J. R., Bhosale A. V. (2007). Bioorg. Med. Chem..

[cit9] He C., Hao J., Xu H., Mo Y. P., Liu H. Y., Han J. J., Lei A. W. (2012). Chem. Commun..

[cit10] Tanganelli S., Fuxe K., Ferraro L., Janson A. M., Bianchi C. (1992). Naunyn-Schmiedebergs Arch. Pharmakol..

[cit11] Man H. W., Schafer P., Wong L. M., Patterson R. T., Corral L. G., Raymon H., Blease K., Leisten J., Shirley M., Tang A. Y., Babusis D. M., Chen R., Stirling D., Muller G. W. (2009). J. Med. Chem..

[cit12] Qiu R., Reddy V. P., Iwasaki T., Kambe N. (2015). J. Org. Chem..

[cit13] Thurow S., Abenante L., Anghinoni J. M., Lenardão E. J. (2022). Curr. Org. Synth..

[cit14] Yu S., Wan B., Li X. (2015). Org. Lett..

[cit15] Xie W., Li B., Wang B. (2016). J. Org. Chem..

[cit16] He G., Zhao Y., Zhang S. (2011). J. Am. Chem. Soc..

[cit17] Xie P., Xie Y., Qian B. (2012). J. Am. Chem. Soc..

[cit18] He J., Li S., Deng Y. (2014). Science.

[cit19] Xu R. S., Wan J. P., Mao H., Pan Y. J. (2010). J. Am. Chem. Soc..

[cit20] Duan F. F., Song S. Q., Xu R. S. (2017). Chem. Commun..

[cit21] Cai R. R., Zhou Z. D., Chai Q. Q., Zhu Y. E., Xu R. S. (2018). RSC Adv..

[cit22] Guan S. L., Chen Y., Wu H. J., Xu R. R., Zhu Y. E., Xing F. X., Tong S. L. (2019). Catalysts.

[cit23] Cai R. R., Wei Q. C., Xu R. S. (2020). RSC Adv..

[cit24] Cai R. R., Zhou Z. D., Chai Q. Q., Zhu Y. E., Xu R. S. (2020). RSC Adv..

[cit25] Zhou X. Y., Xue Y. Q., Cheng Y. Y., Xu R. S. (2021). Arkivoc.

[cit26] Gelinas D. A. (1973). Plant Physiol..

[cit27] Bera D., Sarkar R., Dhar T., Saha P., Ghosh P., Mukhopadhyay C. (2024). Org. Biomol. Chem..

[cit28] Liu H., Liu J., Cheng X., Jia X., Yu L., Xu Q. (2019). ChemSusChem.

[cit29] Lim B. S., Holm R. H. (2001). J. Am. Chem. Soc..

[cit30] Jiang C., Wu Y., Zhang Y., Zong J., Wang N., Liu G., Liu R., Yu H. (2024). Angew. Chem., Int. Ed..

[cit31] Yang Z., Lia J., Yang X. G., Xie X. F., Wu Y. (2005). J. Mol. Catal. A:Chem..

[cit32] Fuson C. R. (1926). J. Am. Chem. Soc..

[cit33] Yamashita K., Matsui M., Agr J. (1960). Chem. Soc. Jpn..

[cit34] Tebben L., Studer A. (2011). Angew Chem. Int. Ed. Engl..

[cit35] Ronga L., Varcamonti M. (2023). Molecules.

[cit36] Padmaja K., Lysenko A. B., Mathur G., Li Q. l., Bocian D. F., Misra V., Lindsey J. S. (2004). J. Org. Chem..

