# Supplementary material for: Simple synthesis of 2-(phenylsulphinyl)benzo[d]oxazole derivatives via a silver-catalysed tandem condensation reaction
Source: RSC Adv. 2025 Feb 19;15(8):5698–702. doi: 10.1039/d5ra00983a (PMC11836957; doi:10.1039/d5ra00983a)
Supplement: RA-015-D5RA00983A-s001 [file RA-015-D5RA00983A-s001.pdf]

Supplementary Information (SI) for RSC Advances.
This journal is © The Royal Society of Chemistry 2025

Supporting Information

Simple synthesis of 2-(phenylsulphinyl)benzo[d]oxazole derivatives via a silver-catalysed tandem condensation reaction

Runsheng Xu,*[a] Qi Hu,a Jiahao Hu,[a] Guangqu Liu[a] and Jin Xu*[a]
College of Life and Health, Huzhou College, Huzhou Zhejiang 211300
Email: xurunsheng@zjhzu.edu.cn, xujin@zjhzu.edu.cn

Contents

General Information.. S2
Procedure for optimization of the reaction condition... S2
General procedures for preparation of 4, 7 and 9.. S4
Analytical Data.. S6
Spectrum..S15

General Information

All reagents used in experiment were obtained from commercial sources and used without further purification. Solvents for chromatography were technical grade and distilled prior for using. Solvent mixtures were understood as volume/volume. Chemical yields refer to pure isolated substances. Catalysts were purchased for analytical reagent. Thin layer chromatography employed glass 0.25 mm silica gel plates with F_{254} indicator, visualized by irradiation with UV light. Reactions were carried out under argon in flame-dried or oven-dried glassware unless otherwise specified. Dichloroethane, dichloromethane, acetonitrile, toluene (after distilling from sodium), dimethyl sulfoxide, and tetrahydrofuran (after distilling from sodium) were dried from 4Å molecular sieves. Synthesis-grade solvents were used after as purchased. Chromatographic purification of products was accomplished using silica gel (300-400 mesh). For thin layer chromatography (TLC) analysis, Merck pre-coated TLC plates (silica gel 60 GF_{254}, 0.25 mm) were employed, using UV light as the visualizing agent. The compounds were isolated using Biotage flash column chromatography.

The NMR spectra were recorded at 500 MHz for ^{1}H, 125 MHz for ^{13}C, and 470 MHz for ^{19}F spectra. The NMRs were recorded in the DMSO-d_6 as solvent. The chemical shift (δ) for ^{1}H NMR and ^{13}C NMR are given in ppm relative to residual signals of the solvents. Coupling constants are given in Hertz (Hz). The following abbreviations are used to indicate the multiplicity: s, singlet; d, doublet; t, triplet; q, quartet; p, pentet; sept, septet; m, multiplet. High-resolution mass spectra (HRMS) were obtained from the High-Resolution Mass Spectrometry using electrospray ionization time-of-flight (ESI-TOF) reflection experiments.

Procedure for optimization of the reaction condition

At the beginning, our investigated with the model reaction of 2-aminophenol **1,** formaldehyde **2** and benzenethiol **3a** to study reaction conditions including the optimization of catalysts, ligands, bases and solvents. As shown in Scheme 1, at the outset, silver salts were used as catalyst (entries 1-4), no desired product was gained when the reaction conducted in the presence of Ag_2O as the catalyst in DMSO (entry

1). AgOAc was proved to be the best efficient catalyst species in this reaction (entry 4). The examination of all available ligands including **L1**-**L6** (entries 4-9), **L4** was proved to be the best efficient catalyst species for this transformation (entry 7). Gratifyingly, the yield of product **4a** was obtained in 12% when the catalyst changed to $AgNO_3$ (entries 2). By screening different bases for C(sp^2)-sulfoxide bonds formation reaction, Cs_2CO_3 was demonstrated to be more suitable base than others such as NaOH, Na_2CO_3 and K_2CO_3 (entries 11-14). The experiment result shows proper solvent was critical for this reaction, when the reactions were conducted in apolar solvent such as CH_3CN, or weak coordination solvent DMF, trace product was detected (entry 14, 15). In addition, replacing DMF with DMSO, gave a better yield, this control experiment suggested that the DMSO was critical for successful for this transformation. Reducing yield was obtained in the reaction operated in 100 °C and 120 °C. Remarkably, no desired product was obtained under O_2 atmosphere (entry 15), indicating that N_2 was essential for present reaction. Finally determine the optimal reaction conditions were AgOAc as the catalyst, L4 as the best ligand, Cs_2CO_3 as the base, the ratio of **1**:**2**:**3a** 1:1.5:1, under N_2, in 110 °C, preparation for 24 hours.

Scheme 1. Optimization of the reaction conditions.[a]

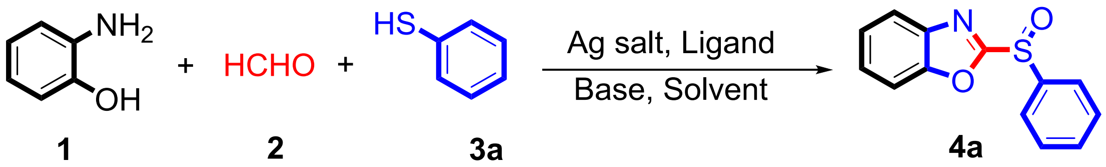

Entry	Ligand	Ag salt	Base	Ratio 1:2:3a	Yield (%)[b]
1	**L1**	Ag_2O	Cs_2CO_3	1:1:1	0
2	**L1**	$AgNO_3$	Cs_2CO_3	1:1:1	14
3	**L1**	$AgBF_4$	Cs_2CO_3	1:1:1	17
4	**L1**	AgOAc	Cs_2CO_3	1:1:1	44
5	**L2**	AgOAc	Cs_2CO_3	1:1:1	31
6	**L3**	AgOAc	Cs_2CO_3	1:1:1	38
7	**L4**	AgOAc	Cs_2CO_3	1:1:1	73

**8**	**L5**	AgOAc	Cs_2CO_3	1:1:1	51
**9**	**L6**	AgOAc	Cs_2CO_3	1:1:1	38
**10**	**L4**	AgOAc	Cs_2CO_3	1:1.5:1	79
**11**	**L4**	AgOAc	NaOH	1:1.5:1	55
**12**	**L4**	AgOAc	Na_2CO_3	1:1.5:1	0
**13**	**L4**	AgOAc	K_2CO_3	1:1.5:1	44
**14**	**L4**	AgOAc	Cs_2CO_3	1:1.5:1	64[d]
**15**	**L4**	AgOAc	Cs_2CO_3	1:1.5:1	71[e]
**16**	**L4**	AgOAc	Cs_2CO_3	1:1.5:1	trace[f]
**17**	**L4**	AgOAc	Cs_2CO_3	1:1.5:1	trace[g]
**18**	**L4**	AgOAc	Cs_2CO_3	1:1.5:1	trace[h]

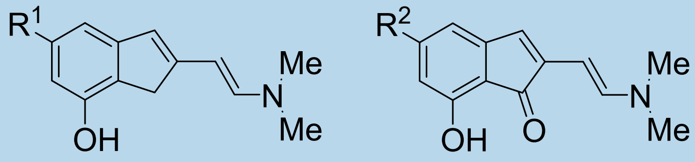

L1: R^1 = H
L2: R^1 = Cl
L3: R^1 = OCH_3

L4: R^2 = H
L5: R^2 = Cl
L6: R^2 = OCH_3

[a] Unless otherwise noted, reactions conditions were **1** (10 mmol)**, 2** (10 mmol), **3a** (10 mmol), Ag salt (10 mol%), ligand (10 mol%), base (2 equiv), solvent (15 mL), 110 °C for 24 h, under N_2. [b] Isolated yield. [d] 100 °C. [c] 120 °C. [f] in CH_3CN. [g] In DMF. [h]Under O_2.

General procedures for preparation of 4, 7 and 9

A mixture of 2-aminophenol **1** (1.09 g, 10 mmol)**,** formaldehyde **2** (0.45 g, 15 mmol) and benzenethiol **3a** (2.43 g, 10 mmol), AgOAc (167 mg, 10 mol%), **L4** (22 mg, 10 mol%), Cs_2CO_3 (6.52 g, 2 equiv), DMSO (15 mL). The tube was evacuated and refilled with N_2 three times. The reaction is carried out under nitrogen protection. The reaction mixture was stirred at 110 °C for 24 h. After it was cooled, the reaction mixture was diluted with 20 mL of ethyl ether for 3 times. The filtrate was washed with water (3×15 mL). The organic phase was dried over Na_2SO_4, filtered, and concentrated under

reduced pressure. and filtered through a pad of silica gel, followed by washing the pad of silica gel with the same solvent (20 mL). The residue was then purified by flash chromatography on silica gel to provide the corresponding product. The pure product 2-(phenylsulfinyl)benzo[d]oxazole (4a) was obtained 1.92 g, 79% yield.

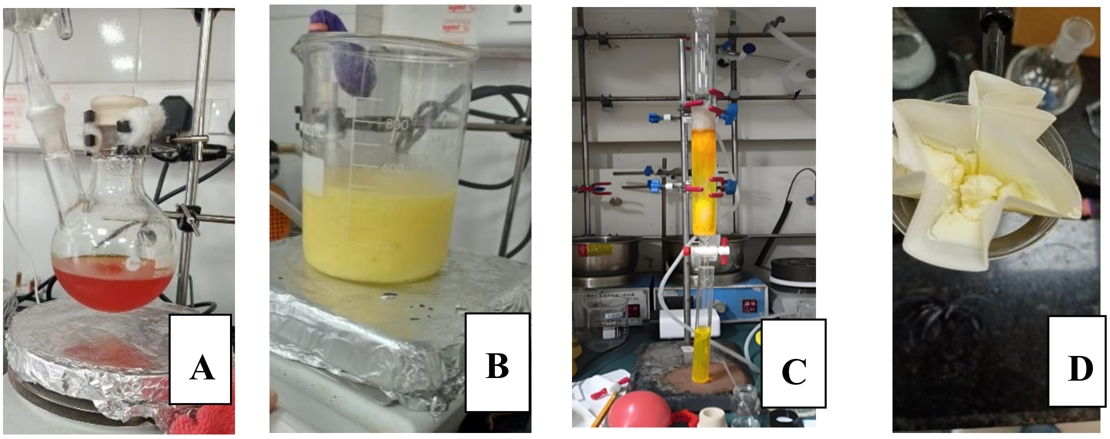

Scheme 2. Synthesis of 2-(phenylsulfinyl)benzo[d]oxazole **3a.**

A. React via standard conditions for 24 hours under nitrogen protection at 110 °C.

B. After cooled, the reaction mixture was diluted with 20 mL of ethyl ether for 3 times.

C. Silica gel column chromatography with gradient elution consisting of ethyl acetate and petroleum ethe mixture solvent.

D. The product obtained by rotary evaporation of solvent.

Preliminary Mechanism Investigation.

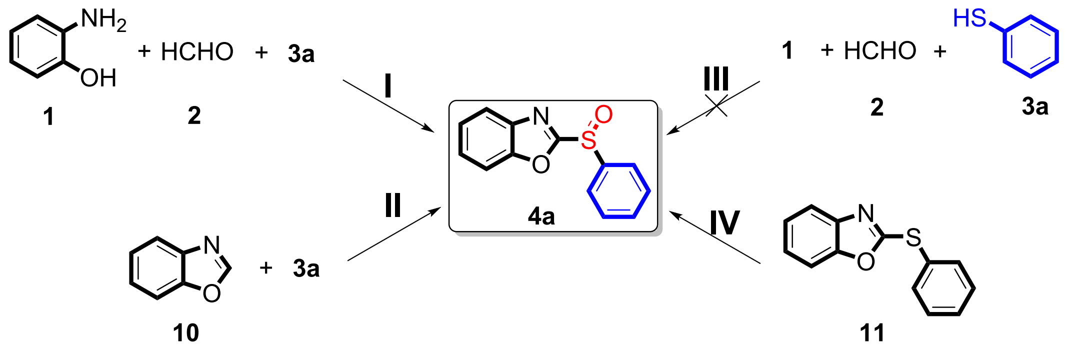

I 1a, 3a (10 mmol), **2** (15 mmol), **L4**, AgOAc (10 mol%), Cs_2CO_3 (2 equiv), DMSO (15 mL).
II 3a (10 mmol), **10** (10 mmol), **L4**, AgOAc (10 mol%), Cs_2CO_3 (2 equiv), DMSO (15 mL), **4a** (71%).
III 1a, 3a (10 mmol), **2** (15 mmol), **L4,** AgOAc (10 mol%), Cs_2CO_3 (2 equiv), DMSO (15 mL), **4a** (0).
IV 11 (10 mmol), AgOAc (10 mol%), **L4**, Cs_2CO_3 (2 equiv), DMSO (15 mL), **4a** (58%).

Scheme 3. Preliminary mechanism investigation.

To obtain the preliminary results of the reaction mechanism, some additional reactions were been done, Scheme 2. At first, the model reaction (Scheme 2I) **1a** (10 mmol), **3a** (10 mmol), **2** (15 mmol), **L4** (10 mol%), AgOAc (10 mol%), Cs_2CO_3 (2 equiv), DMSO (15 mL). After it was cooled, the reaction mixture was diluted with 10 mL of ethyl ether, and filtered through a pad of silica gel, followed by washing the pad of silica gel with the same solvent (20 mL). The filtrate was washed with water (3×15 mL). The organic phase was dried over Na_2SO_4, filtered, and concentrated under reduced pressure. The residue was then purified by flash chromatography on silica gel to provide the corresponding product.

Other reactions were conducted in other three parallel reactions, Scheme 2II, Scheme 2III and Scheme 2IV). However, scheme 2II, results show that benzo[d]oxazole **10** reacted with **3a** promoted by hydrogen peroxide under our standard conditions, successfully obtained the target product **4a** in 71% yield, **3a** (10 mmol), **10** (10 mmol), **L4** (10 mol%), AgOAc (10 mol%), Cs_2CO_3 (2 equiv), DMSO (15 mL). After it was cooled, the reaction mixture was diluted with 10 mL of ethyl ether, and filtered through a pad of silica gel, followed by washing the pad of silica gel with the same solvent (20 mL). The filtrate was washed with water (3×15 mL). The organic phase was dried over Na_2SO_4, filtered, and concentrated under reduced pressure. The residue was then purified by flash chromatography on silica gel to provide the corresponding product.

Forthermore, Scheme 2IV, **11** reacted promoted under our standard conditions, successfully obtained the target product **4a** in 58% yield, **11** (10 mmol), AgOAc (10 mol%), **L4**, Cs_2CO_3 (2 equiv), DMSO (15 mL), which indicated that the reaction first undergoes a condensation reaction process. And those results also indicated that DMSO was the necessary solvent for this reaction. After it was cooled, the reaction mixture was diluted with 10 mL of ethyl ether, and filtered through a pad of silica gel, followed by washing the pad of silica gel with the same solvent (20 mL). The filtrate was washed with water (3×15 mL). The organic phase was dried over Na_2SO_4, filtered, and concentrated under reduced pressure. The residue was then purified by flash

chromatography on silica gel to provide the corresponding product.

Analytical Data

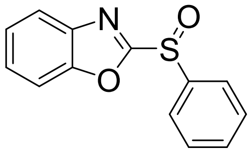

2-(phenylsulfinyl)benzo[d]oxazole (**4a**). Yellow solid, 1.92 g, 79% yield, mp 141-142 °C;

^{1}HNMR (500 MHz, DMSO-d_6): δ 7.94 (d, *J*=8.0 Hz, 1H), 7.88 (d, *J*=8.0 Hz, 1H), 7.58 (m, 2H), 7.57-7.51 (m, 3H), 7.43 (m, 1H), 7.33 (s, 1H); ^{13}C NMR (125 MHz, DMSO-d_6): δ 162.24, 154.02, 136.22, 135.89, 130.25, 130.18, 126.26, 124.51, 121.61, 121.40;

ESI HRMS *m/z*: Calcd for $C_{13}H_{10}NO_2S^+$ [M+H]$^+$: 244.0432; Found 244.0328.

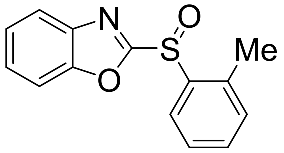

2-(o-tolylsulfinyl)benzo[d]oxazole (**4b**). Yellow oil liquid, 2.11 g, 82% yield;

^{1}HNMR (500 MHz, DMSO-d_6): δ 7.92 (d, *J*=8.0 Hz, 1H), 7.85 (d, *J*=8.0 Hz, 1H), 7.65 (d, *J* = 8.0 Hz, 1H), 7.44-7.39 (m, 3H), 7.27-7.22 (m, 2H), 2.55 (s, 3H); ^{13}C NMR (125 MHz, DMSO-d_6): δ 162.95, 154.78, 143.04, 138.25, 136.60, 130.95, 130.90, 127.67, 127.34, 125.99, 124.21, 121.87, 120.76, 23.15;

ESI HRMS *m/z*: Calcd for $C_{14}H_{12}NO_2S^+$ [M+H]$^+$: 258.0589; Found 258.0593.

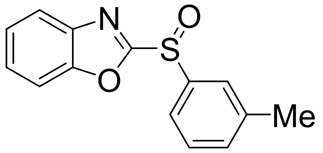

2-(m-tolylsulfinyl)benzo[d]oxazole (**4c**). Yellow oil liquid, 2.24 g, 87% yield;

^{1}H NMR(500 MHz, DMSO-d_6): δ 7.95 (d, *J*=8.0 Hz, 1H), 7.89 (d, *J*=8.0 Hz, 1H), 7.66 (s, 1H), 7.64 (d, *J*=7.5 Hz, 1H), 7.47-7.33 (m, 4H), 2.36 (s, 3H); ^{13}C NMR (125 MHz, DMSO-d_6): δ 162.53, 154.01, 139.78, 136.61, 135.88, 133.32, 130.97, 129.95, 126.21, 125.73, 124.42, 121.62, 121.35, 20.73;

ESI HRMS *m/z*: Calcd for $C_{14}H_{12}NO_2S^+$ [M+H]$^+$: 258.0589; Found 258.0593.

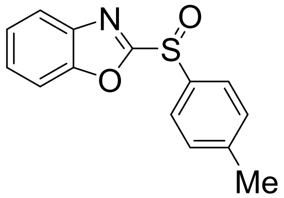

2-(p-tolylsulfinyl)benzo[d]oxazole (**4d**). Yellow solid, 2.34 g, 91% yield，mp 112-114°C;

^{1}H NMR (500 MHz, DMSO-d_6): δ 7.90 (d, *J*=8.0 Hz, 1H), 7.71 (d, *J*=7.5 Hz, 2H), 7.65 (d, *J*=7.5 Hz, 1H), 7.38 (m, 1H), 7.26-7.13 (m, 3H), 2.42 (s, 3H); ^{13}C NMR (125MHz, DMSO-d_6): δ 163.71, 154.69, 140.58, 136.76, 136.55, 130.77, 125.96, 124.20, 122.91, 121.96, 120.73, 21.41;

ESI HRMS *m/z*: Calcd for $C_{14}H_{12}NO_2S^+$ [M+H]$^+$: 258.0589; Found 258.0593.

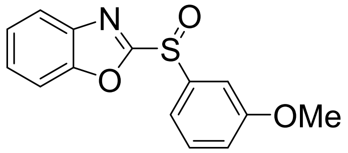

2-((3-methoxyphenyl)sulfinyl)benzo[d]oxazole (**4e**). Yellow oil liquid, 2.05 g, 75% yield;

^{1}H NMR (500 MHz, DMSO-d_6): δ 7.97-7.89 (m, 2H), 7.45-7.42 (m, 5H), 7.34 (s, 1H), 3.81 (s, 3H); ^{13}C NMR (125 MHz, DMSO-d_6): δ 162.19, 159.94, 154.00, 135.88, 131.07, 128.15, 126.72, 126.26, 124.51, 121.69, 121.39, 121.19, 116.14, 55.44;

ESI HRMS *m/z*: Calcd for $C_{14}H_{12}NO_3S^+$ [M+H]$^+$: 274.0538; Found 274.0534.

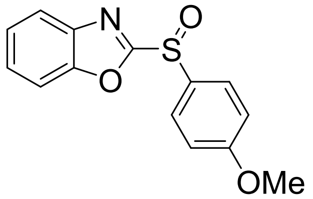

2-((4-methoxyphenyl)sulfinyl)benzo[d]oxazole (**4f**). Yellow oil liquid, 2.46 g, 90% yield;

^{1}H NMR(500 MHz, DMSO-d_6): δ 7.94 (d, *J*=8.0 Hz, 1H), 7.87 (d, *J*=8.0 Hz, 1H), 7.78 (m, 2H), 7.45 (m, 1H), 7.32 (m, 1H), 7.08 (d, 2H), 3.84 (s, 3H); ^{13}CNMR (125 MHz, DMSO-d_6): δ 164.47, 161.08, 154.28, 138.57, 135.73, 126.19, 124.26, 121.19, 115.92, 115.89, 55.40;

ESI HRMS *m/z*: Calcd for $C_{14}H_{12}NO_3S^+$ [M+H]$^+$: 274.0538; Found 274.0534.

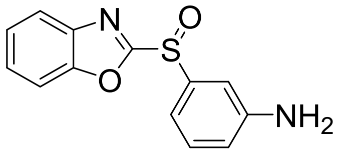

3-(benzo[d]oxazol-2-ylsulfinyl)aniline (**4g**). Yellow oil liquid. 2.20 g, 85% yield;

^{1}H NMR (500 MHz, DMSO-d_6): δ 7.96 (d, *J*=8.0 Hz, 1H), 7.87 (d, *J*=8.0 Hz, 1H), 7.42 (m, 1H), 7.33 (m, 1H), 7.17 (m, 1H), 7.07 (s, 1H), 6.94 (d, *J*=7.5 Hz, 1H), 6.75 (m, 1H), 5.45 (s, 2H); ^{13}C NMR (125 MHz, DMSO-d_6): δ 163.14, 154.11, 150.29, 135.95, 130.50, 126.16, 125.93, 124.35, 122.83, 121.60, 121.28, 120.82, 115.58;

ESI HRMS *m/z*: Calcd for $C_{13}H_{11}N_2O_2S^+$ $[M+H]^+$: 259.0541; Found 259.0537.

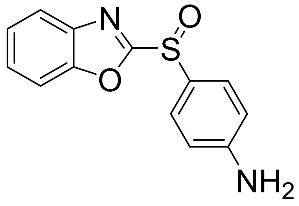

4-(benzo[d]oxazol-2-ylsulfinyl)aniline (**4h**). Yellow oil liquid, 2.38 g, 92% yield;

^{1}H NMR (500 MHz, DMSO-d_6): δ 7.92 (d, *J*=8.0 Hz, 1H), 7.84 (d, *J*=8.5 Hz, 1H), 7.46 (m, 2H), 7.41 (t, 1H), 7.8 (m, 1H), 6.68 (d, *J*=8.0 Hz, 2H), 5.72 (s, 2H); ^{13}C NMR (125 MHz, DMSO-d_6): δ 166.94, 154.58, 151.20, 138.29, 135.79, 126.02, 123.98, 121.52, 121.01, 115.10, 108.83;

ESI HRMS *m/z*: Calcd for $C_{13}H_{11}N_2O_2S^+$ $[M+H]^+$: 259.0541; Found 259.0537.

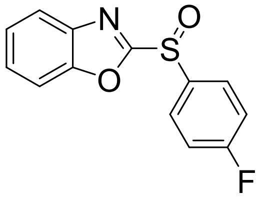

2-((4-fluorophenyl)sulfinyl)benzo[d]oxazole (**4i**). Yellow oil liquid, 1.93 g, 74% yield;

^{1}H NMR (500 MHz, DMSO-d_6): δ 7.97 (m, 4H), 7.47 (m, 1H), 7.40-7.34 (m, 3H); ^{13}C NMR (125 MHz, DMSO-d_6): δ 164.39, 162.59, 162.41, 154.05, 139.11, 135.81, 126.28, 124.49, 121.67, 121.37, 121.29, 117.49, 117.31; ^{19}F NMR (470 MHz, DMSO-d_6): δ -110.15 (s, 1F);

ESI HRMS *m/z*: Calcd for $C_{13}H_9FNO_2S^+$ $[M+H]^+$: 262.0338; Found 262.0334.

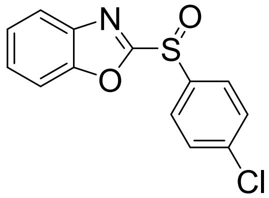

2-((4-chlorophenyl)sulfinyl)benzo[d]oxazole (**4j**). Yellow oil liquid, 2,17 g, 78% yield;

^{1}H NMR (500 MHz, DMSO-d_6): δ 7.98 (d, *J*=8.0 Hz, 1H), 7.91 (d, *J*= 8.5Hz, 1H), 7.86 (d, *J*=8.5 Hz, 2H), 7.57 (d, *J*=8.5 Hz, 2H), 7.48 (m, 1H), 7.38 (m, 1H); ^{13}C NMR(125 MHz, DMSO-d_6): δ 161.63, 153.95, 137.96, 135.91, 135.43, 130.15, 126.33, 124.89, 124.62, 121.71, 121.47;

ESI HRMS *m/z*: Calcd for $C_{13}H_9ClNO_2S^+$ $[M+H]^+$: 278.0043; Found 278.0047.

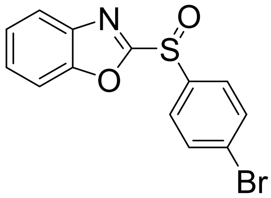

2-((4-bromophenyl)sulfinyl)benzo[d]oxazole (**4k**). Yellow oil liquid, 2.45 g, 76% yield;

^{1}H NMR (500 MHz, DMSO-d_6): δ 7.96 (d, *J*=8.0 Hz, 1H), 7.88 (d, *J*=8.0 Hz, 1H), 7.77 (m, 4H), 7.45 (m, 1H), 7.35 (m, 1H); ^{13}C NMR (125 MHz, DMSO-d_6): δ 161.42, 153.93, 138.09, 135.93, 133.09, 126.31, 125.44, 124.62, 124.16, 121.69, 121.48;

ESI HRMS *m/z*: Calcd for $C_{13}H_9BrNO_2S^+$ $[M+H]^+$: 321.9537; Found 321.9533.

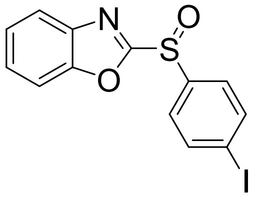

2-((4-iodophenyl)sulfinyl)benzo[d]oxazole (**4l**). White solid, 2.66 g, 72% yield, mp 146-147 °C;

^{1}H NMR (500 MHz, DMSO-d_6): δ 7.97 (d, *J*=7.5 Hz, 1H), 7.90-7.85 (m, 2H), 7.76 (m, 1H), 7.61 (d, *J*=7.5 Hz, 1H), 7.47-7.44 (m, 2H), 7.37-7.32 (m, 1H); ^{13}C NMR (125 MHz, DMSO-d_6): δ 161.34, 153.93, 138.92, 138.58, 137.94, 137.02, 135.80, 132.78, 126.31, 124.61, 121.48;

ESI HRMS *m/z*: Calcd for $C_{13}H_9INO_2S^+$ $[M+H]^+$: 369.9399; Found 369.9395.

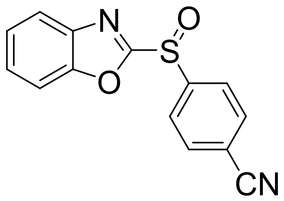

4-(benzo[d]oxazol-2-ylsulfinyl)benzonitrile (**4m**). Yellow solid, 1.88 g, 70% yield, mp 125-126 °C;

^1H NMR (500 MHz, DMSO-d_6): δ 7.97-7.95 (m, 4H), 7.81 (d, *J*=8.5 Hz, 1H), 7.64 (d, *J*=8.5Hz, 1H), 7.51(m, 1H), 7.43(m, 1H); ^{13}C NMR (125 MHz, DMSO-d_6): δ 158.57, 153.66, 136.34, 135.17, 134.00, 133.31, 133.14, 126.51, 125.14, 121.90, 111.95, 110.05;

ESI HRMS *m/z*: Calcd for $C_{14}H_9N_2O_2S^+$ $[M+H]^+$: 269.0385; Found 269.0387.

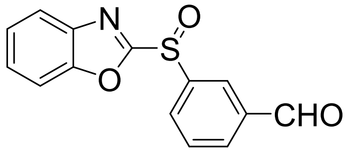

3-(benzo[d]oxazol-2-ylsulfinyl)benzaldehyde (**4n**). Yellow oil liquid, 1.99 g, 73% yield;

^1H NMR (500 MHz, DMSO-d_6): δ 10.08 (s, 1H), 8.35 (s, 1H), 8.18 (d, *J*=7.5 Hz, 1H), 8.09 (d, *J*=8.0 Hz, 1H), 7.97 (d, *J*=8.0 Hz, 1H), 7.91 (d, *J*=8.5 Hz, 1H), 7.76 (m, 1H), 7.49 (m, 1H), 7.39 (m, 1H); ^{13}C NMR (125 MHz, DMSO-d_6): δ 192.38, 160.95, 153.88, 141.61, 137.52, 136.66, 135.98, 130.65, 127.59, 126.38, 124.74, 121.75;

ESI HRMS *m/z*: Calcd for $C_{14}H_{10}NO_3S^+$ $[M+H]^+$: 272.0381; Found 272.0385.

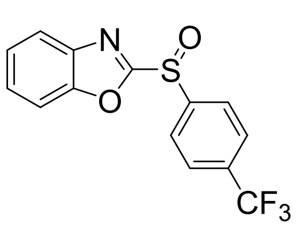

2-((4-(trifluoromethyl)phenyl)sulfinyl)benzo[d]oxazole (**4o**). Yellow solid, 2.40 g, 77% yield, mp 105-106 °C;

^1H NMR (500 MHz, DMSO-d_6): δ 8.05 (m, 3H), 7.96 (d, *J*=8.5 Hz, 1H), 7.84 (m, 2H), 7.51 (m, 1H), 7.41 (m, 1H); ^{13}C NMR (125 MHz, DMSO-d_6): δ 159.62, 153.76, 136.17,

135.93, 132.23, 129.80, 126.65, 126.43, 124.93, 122.78, 121.82, 121.73; ^{19}F NMR (470 MHz, DMSO-d_6): δ 61.40 (s, 3F);

ESI HRMS *m/z*: Calcd for $C_{14}H_9F_3NO_2S^+$ [M+H]$^+$: 312.0306; Found 312.0302.

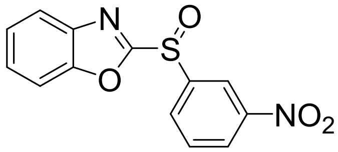

2-((3-nitrophenyl)sulfinyl)benzo[d]oxazole (**4p**). Yellow oil liquid, 1.70 g, 59% yield;

^{1}H NMR (500 MHz, DMSO-d_6): δ 8.27-8.18 (m, 2H), 8.07-7.99 (m, 4H), 7.53 (m, 1H), 7.45 (d, *J*=7.0 Hz, 1H); ^{13}C NMR (125 MHz, DMSO-d_6): δ 157.87, 153.60, 147.74, 136.63, 136.49, 135.05, 133.38, 126.58, 125.30, 124.48, 122.04, 121.95, 121.93;

ESI HRMS *m/z*: Calcd for $C_{13}H_9N_2O_4S^+$ [M+H]$^+$: 289.0283; Found 289.0279.

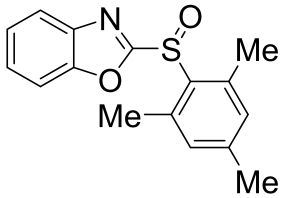

2-(mesitylsulfinyl)benzo[d]oxazole (**4q**). Yellow solid, 2.54 g, 88% yield, mp 90-91 °C;

^{1}H NMR (500 MHz, DMSO-d_6): δ 7.89 (d, *J*=8.0 Hz, 1H), 7.85 (d, *J*=8.0 Hz, 1H), 7.42 (m, 1H), 7.31 (m, 1H), 7.15 (s, 2H), 2.46 (s, 6H), 2.32 (s, 3H); ^{13}C NMR (125 MHz, DMSO-*d6*): δ 163.43, 154.48, 143.30, 140.70, 135.65, 129.27, 126.11, 125.42, 124.06, 121.60, 121.02, 23.78, 20.67;

ESI HRMS *m/z*: Calcd for $C_{16}H_{16}NO_2S^+$ [M+H]$^+$: 286.0902; Found 286.0900.

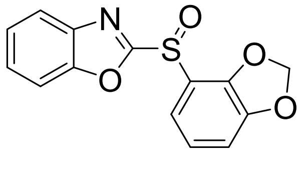

2-(benzo[d][1,3]dioxol-4-ylsulfinyl)benzo[d]oxazole (**4r**). Yellow oil liquid, 2.36 g, 82% yield;

^{1}H NMR (500MHz, DMSO-d_6): δ 7.96 (d, *J*=8.0 Hz, 1H), 7.86 (d, *J*=8.0 Hz, 1H), 7.46-7.43 (m, 2H), 7.37 (d, *J*=8.0 Hz, 1H), 7.34 (d, *J*=8.0 Hz, 1H), 7.09 (d, *J*=8.0 Hz, 1H), 6.16 (s, 2H); ^{13}C NMR (125MHz, DMSO-d_6): δ 163.94, 154.19, 149.46, 148.42,

135.77, 131.47, 126.21, 124.33, 121.65, 121.25, 116.81,116.38, 109.92, 101.95;

ESI HRMS *m/z*: Calcd for $C_{14}H_{10}NO_4S^+$ [M+H]$^+$: 288.0331; Found 2888.0327.

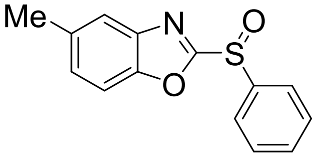

5-methyl-2-(phenylsulfinyl)benzo[d]oxazole (**7a**). Yellow oil liquid, 2.14 g, 83% yield;

^{1}H NMR (500 MHz, DMSO-d_6): δ 7.84 (d, *J*=7.5 Hz, 2H), 7.76 (d, *J*=8.5 Hz, 1H), 7.72 (s, 1H), 7.58-7.50 (m, 3H), 7.27 (d, *J*=8.5 Hz, 1H), 2.38 (s, 3H); ^{13}C NMR (125 MHz, DMSO-d_6): δ 165.56, 157.43, 141.35, 141.30, 139.52, 135.39, 132.86, 132.86, 131.49, 126.43, 126.25, 26.15;

ESI HRMS *m/z*: Calcd for $C_{14}H_{12}NO_2S^+$ [M+H]$^+$: 258.0589; Found 258.0584.

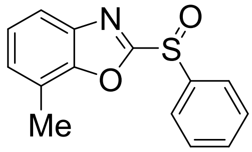

3-methyl-2-(phenylsulfinyl)benzo[d]oxazole (**7b**). Yellow oil liquid, 2.22 g, 86% yield;

^{1}H NMR (500MHz, DMSO-d_6): δ 7.86 (d, *J*=7.5 Hz, 2H), 7.74 (d, *J*=7.5 Hz, 1H), 7.58-7.51 (m, 3H), 7.27-7.21 (m, 2H), 2.62 (s, 3H); ^{13}C NMR (125 MHz, DMSO-d_6): δ 160.76, 153.29, 136.10, 135.74, 130.97, 130.16, 126.67, 126.30, 124.48, 118.97, 17.89;

ESI HRMS *m/z*: Calcd for $C_{14}H_{12}NO_2S^+$ [M+H]$^+$: 258.0589; Found 258.0584.

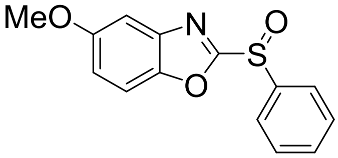

5-methoxy-2-(phenylsulfinyl)benzo[d]oxazole (**7c**). Yellow oil liquid, 2.16 g, 79% yield;

^{1}H NMR (500 MHz, DMSO-d_6): δ 7.82-7.81 (m, 3H), 7.54-7.48 (m, 4H), 7.07 (d, *J*=8.5 Hz, 1H), 3.78 (s, 3H); ^{13}C NMR (125 MHz, DMSO-d_6)： δ 157.50, 156.84, 148.52, 137.58, 135.73, 130.07, 129.91, 126.60, 122.05, 115.15, 104.69, 55.65;

ESI HRMS *m/z*: Calcd for $C_{14}H_{13}NO_3S^+$ [M+H]$^+$: 274.0538; Found 274.0534.

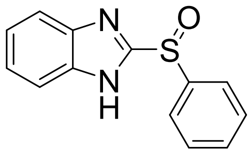

2-(phenylsulfinyl)-1H-benzo[d]imidazole (**9a**). Yellow oil liquid, 2.08 g, 86% yield; ^{1}H NMR (500 MHz, DMSO-d_6): δ 12.77 (s, 1H), 7.59 (d, *J*=8.0 Hz, 3H), 7.44 (d, *J*=8.0 Hz, 1H), 7.36 (s, 3H), 7.17 (m, 2H); ^{13}C NMR (125 MHz, DMSO-d_6): δ 141.14, 132.73, 129.61, 128.03, 127.94, 122.42, 121.42, 118.26, 110.93;

ESI HRMS *m/z*: Calcd for $C_{13}H_{11}N_2OS^+$ $[M+H]^+$: 243.0592; Found 243.0588.

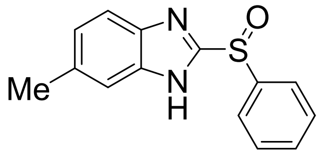

6-methyl-2-(phenylsulfinyl)-1H-benzo[d]imidazole (**9b**). Yellow oil liquid, 2.18 g, 85% yield;

^{1}H NMR (500 MHz, DMSO-d_6): δ 12.69 (s, 1H), 7.57-7.55 (m, 2H), 7.35-7.34 (m, 5H), 7.01 (m, 1H), 2.39 (s, 3H); ^{13}C NMR (125 MHz, DMSO-d_6): δ 173.11, 169.94, 139.95, 134.69, 132.38, 129.56, 128.34, 127.85, 123.12, 117.57, 110.65, 21.17;

ESI HRMS *m/z*: Calcd for $C_{14}H_{10}N_2OS^+$ $[M+H]^+$: 257.0749; Found 257.0745.

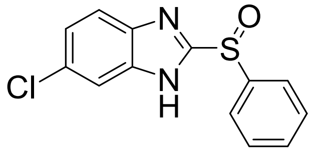

6-chloro-2-(phenylsulfinyl)-1H-benzo[d]imidazole (**9c**). Yellow oil liquid, 2.24 g, 81% yield;

^{1}H NMR (500 MHz, DMSO-d_6): δ 12.91 (s, 1H), 7.62 (m, 3H), 7.38 (m, 4H), 7.19 (d, *J*=7.5 Hz, 1H); ^{13}C NMR (125 MHz, DMSO-d_6) δ 133.23, 130.10, 130.06, 129.66, 128.31, 127.29, 123.74, 123.74, 117.62, 112.19, 110.57;

ESI HRMS *m/z*: Calcd for $C_{13}H_{10}ClN_2OS^+$ $[M+H]^+$: 277.0202; Found 277.0118.

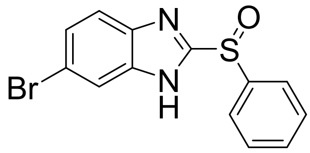

6-bromo-2-(phenylsulfinyl)-1H-benzo[d]imidazole (**9d**) Yellow oil liquid, 2.63 g, 82% yield;

^1H NMR (500 MHz, DMSO-d_6): δ 12.90 (s, 1H), 7.62 (m, 3H), 7.39 (m, 4H), 7.31 (d, *J*=8.0 Hz, 1H); ^{13}C NMR (125 MHz, DMSO-d_6): δ 133.23, 130.13, 130.12, 130.07, 129.67, 129.43, 129.37, 128.32, 127.26, 123.88, 123.75;

ESI HRMS *m/z*: Calcd for $C_{13}H_{10}BrN_2OS^+$ $[M+H]^+$: 320.9697; Found 320.9693.

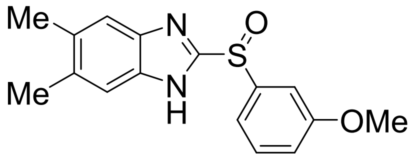

2-((3-methoxyphenyl)sulfinyl)-5,6-dimethyl-1H-benzo[d]imidazole (**9e**). oil liquid, 2.68 g, 89% yield;

^1H NMR (500 MHz, DMSO-d_6): δ 12.59 (s, 1H), 7.53 (m, 2H), 7.38-7.34 (m, 4H), 7.22 (s, 1H), 2.29 (s, 6H); ^{13}C NMR (125 MHz, DMSO-d_6): δ 142.93, 139.10, 132.04, 129.83, 129.54, 128.74, 127.70, 118.37, 111.01, 99.49, 19.86;

ESI HRMS *m/z*: Calcd for $C_{16}H_{16}N_2O_2S^+$ $[M+H]^+$: 301.1011; Found 301.1014.

Spectrums

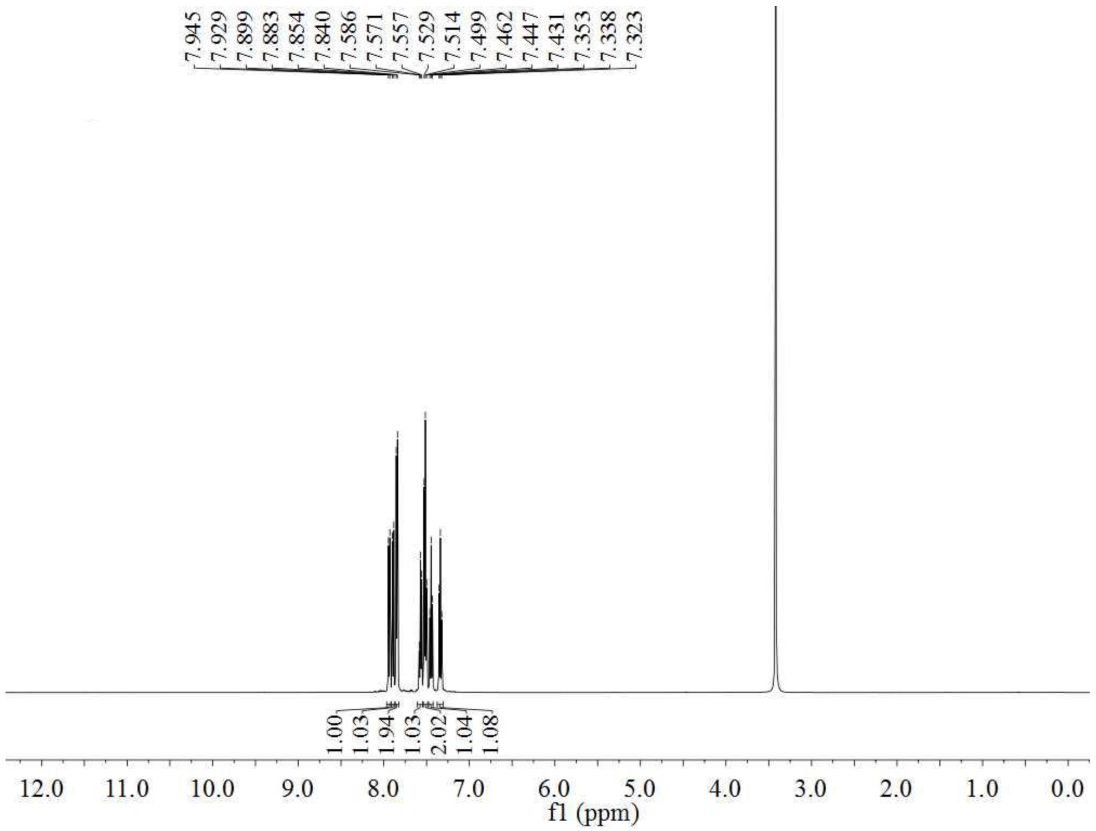

Figure 1. **4a** ^{1}H NMR

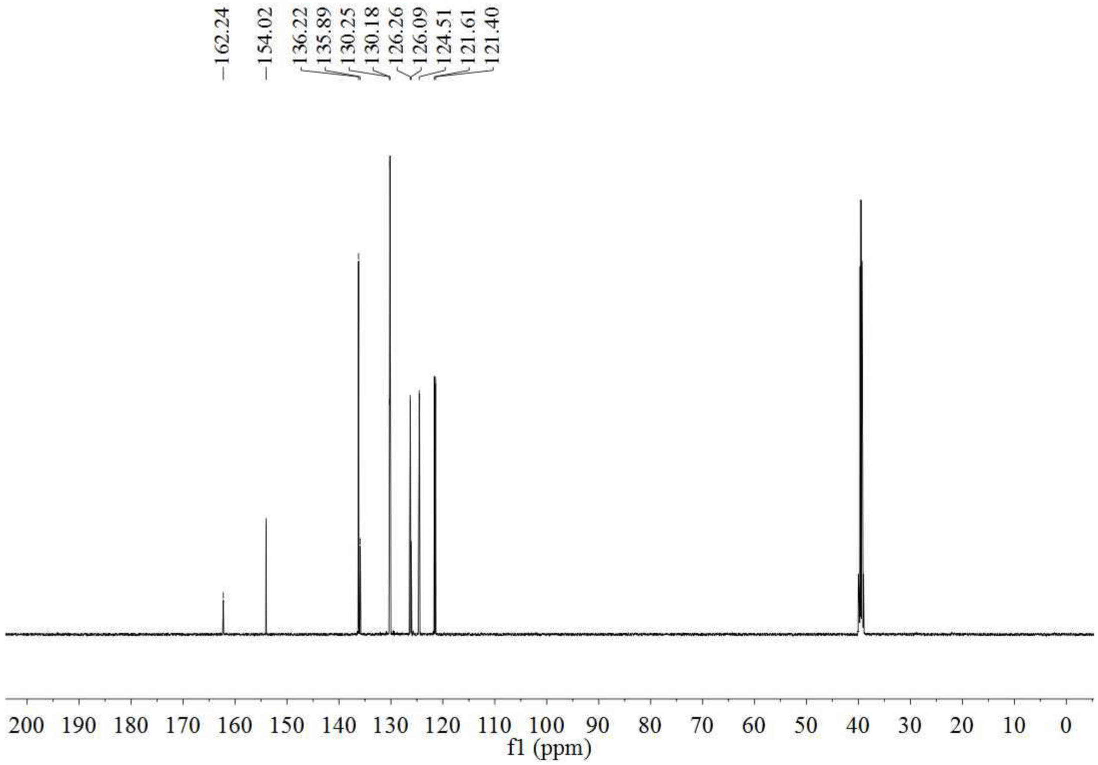

Figure 2. **4a** ^{13}C NMR

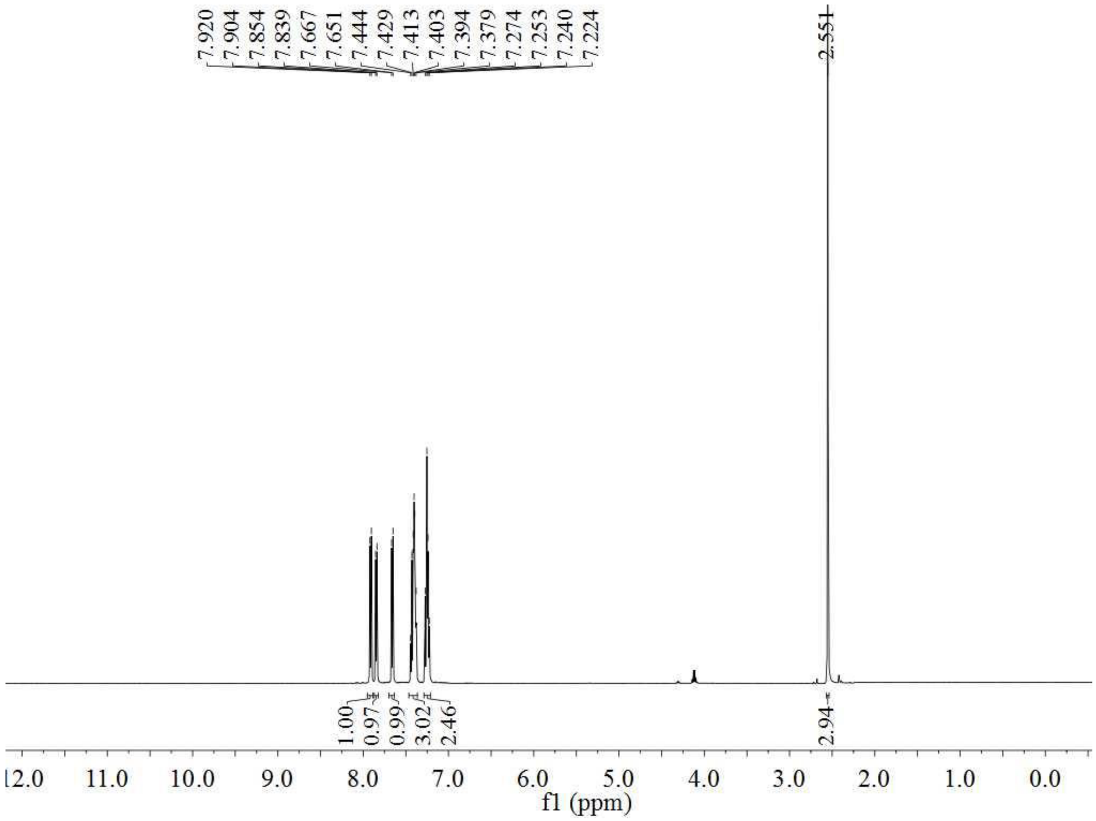

Figure 3. **4b** ^{1}H NMR

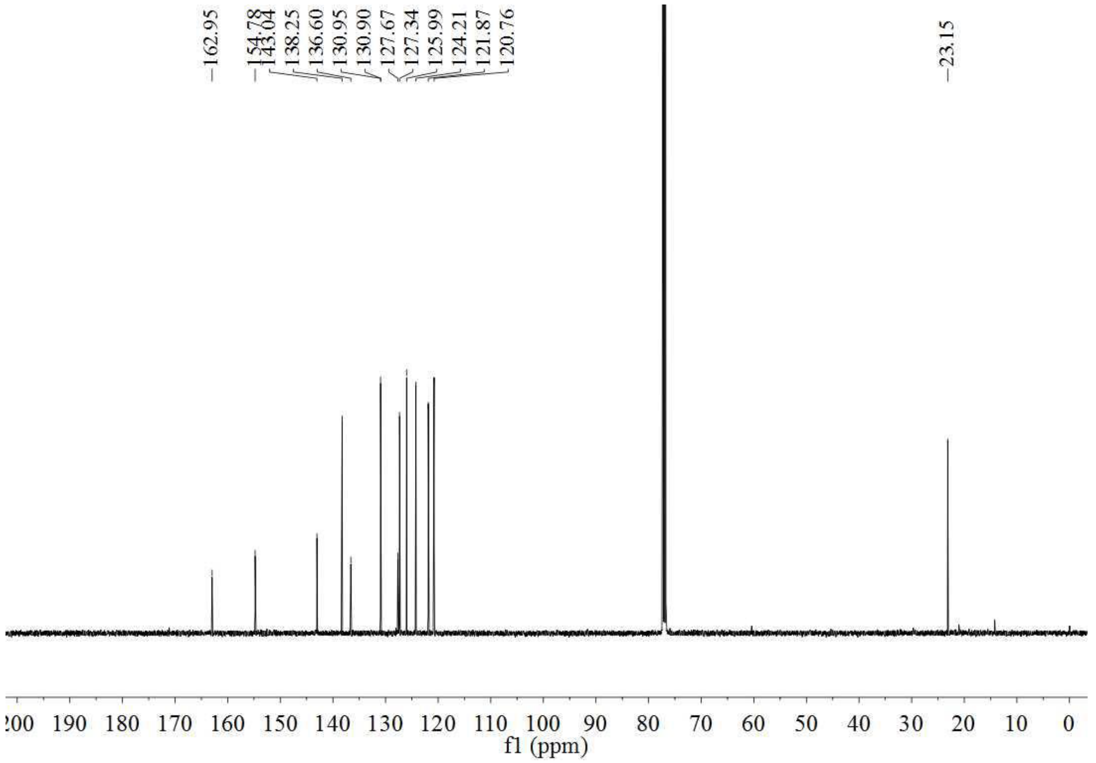

Figure 4. **4b** ^{13}C NMR

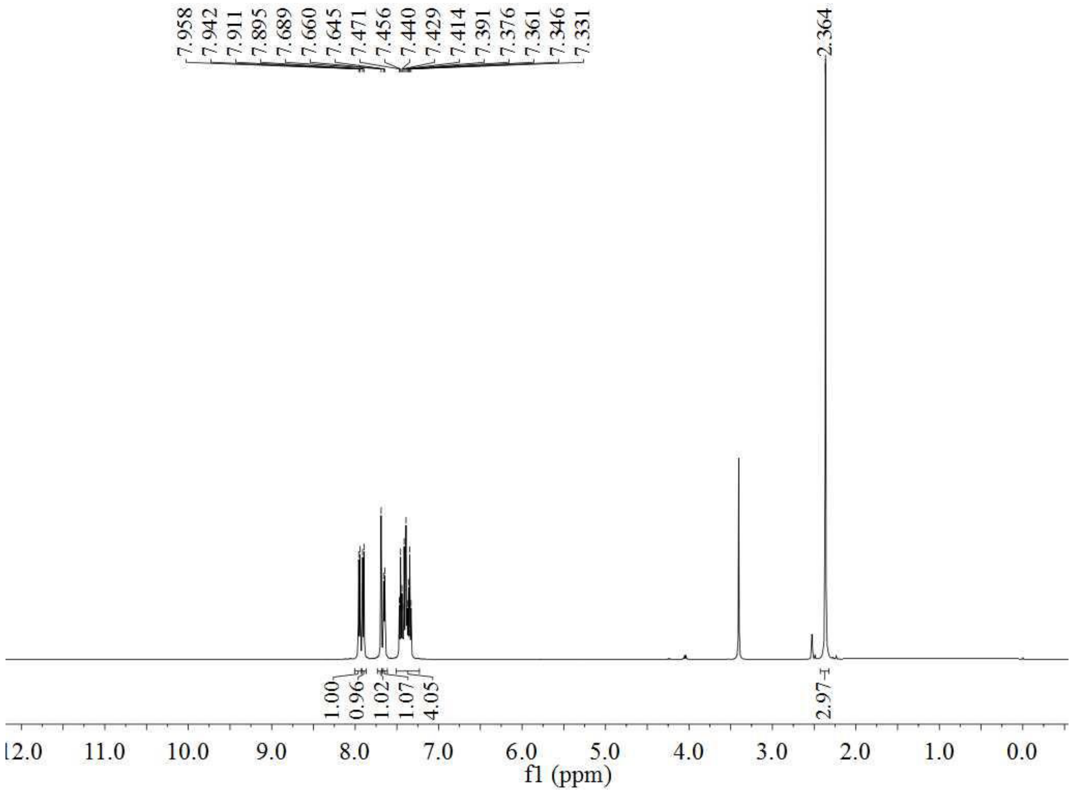

Figure 5. **4c** ^{1}H NMR

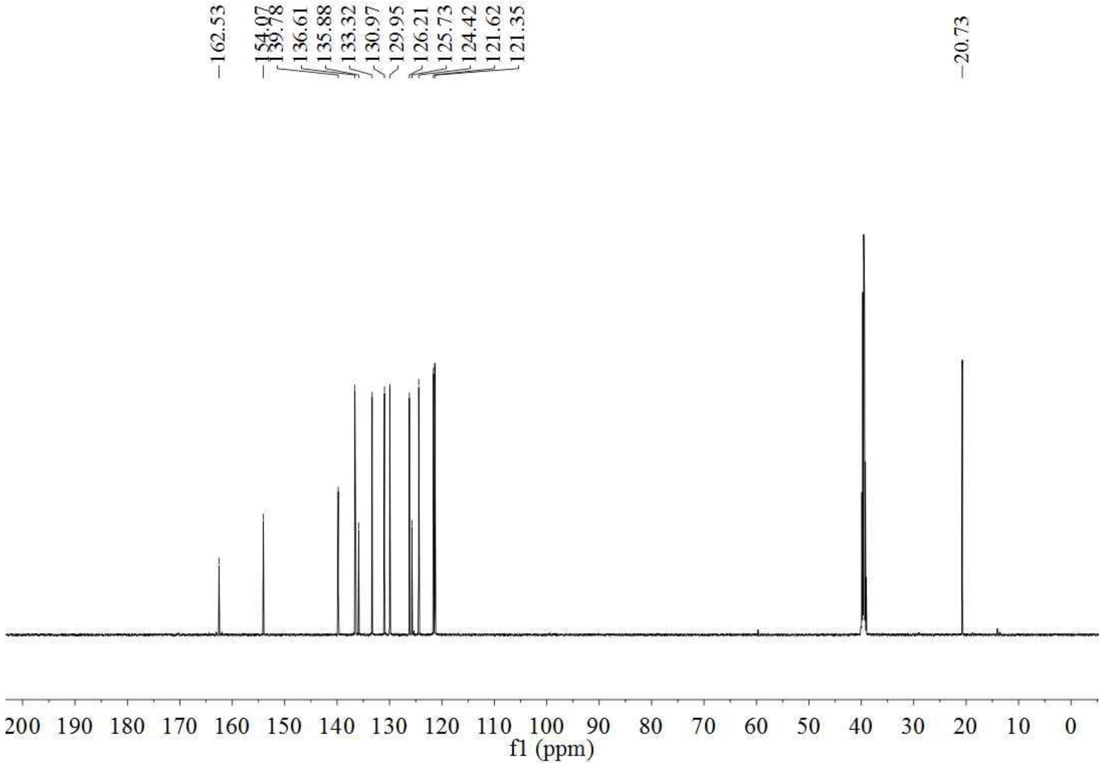

Figure 6. **4c** ^{13}C NMR

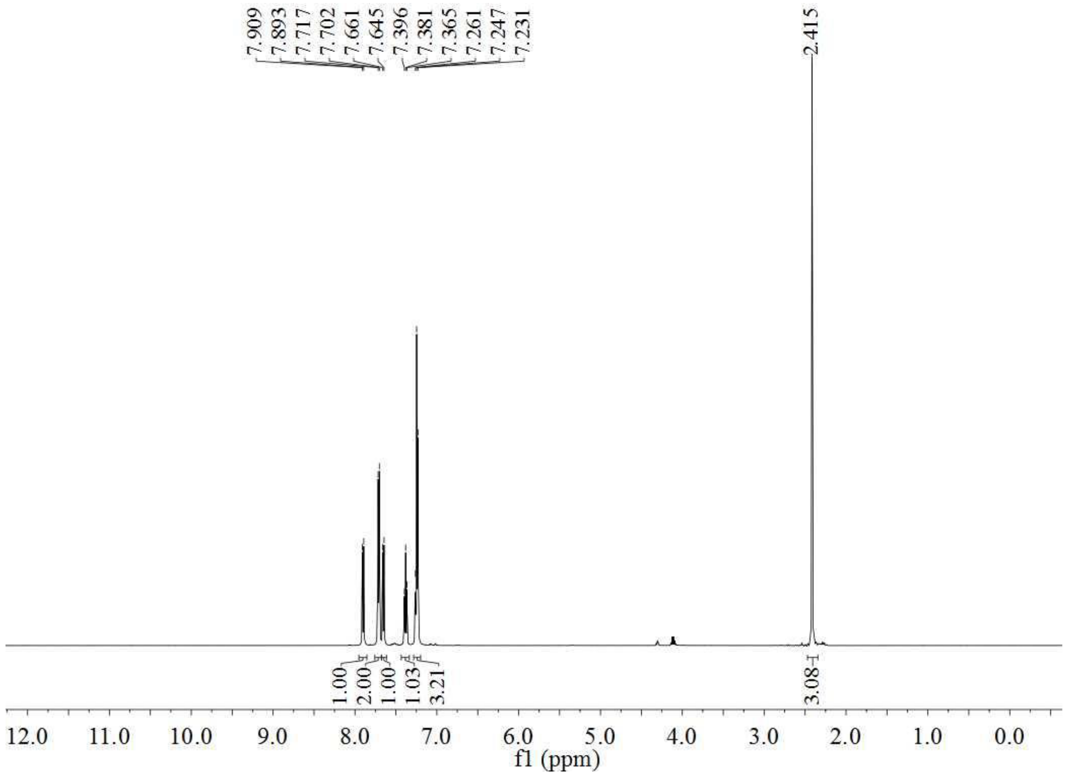

Figure 7. **4d** ^{1}H NMR

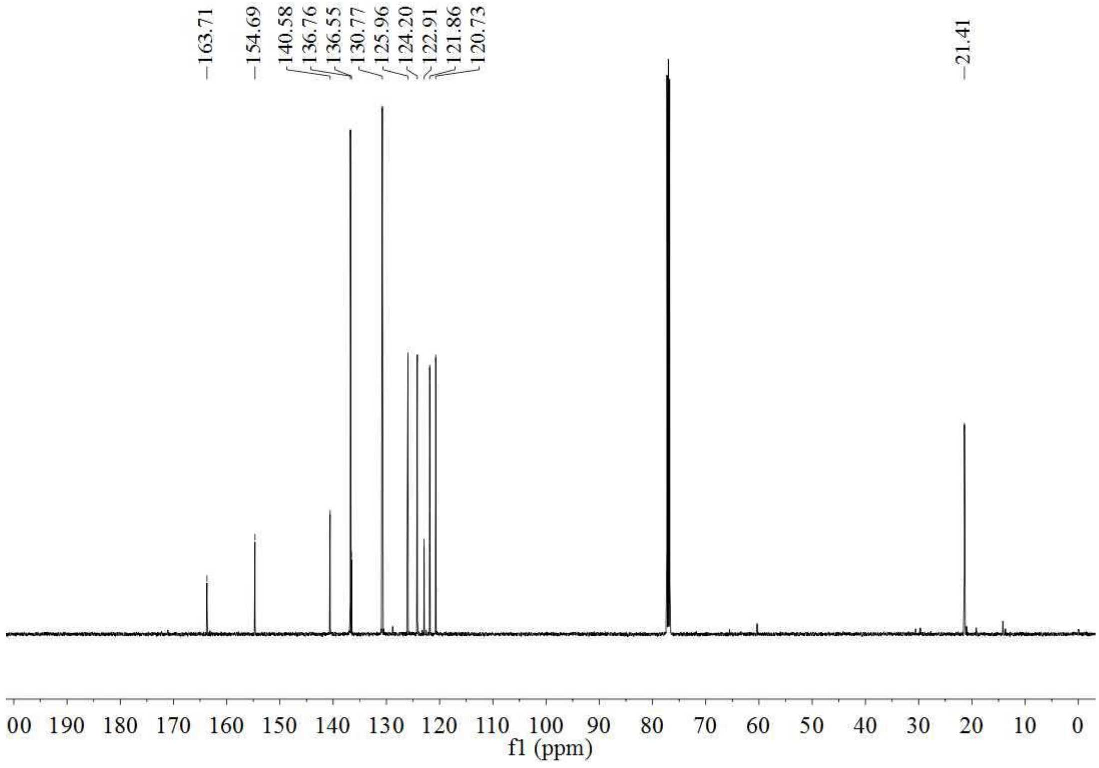

Figure 8. **4d** ^{13}C NMR

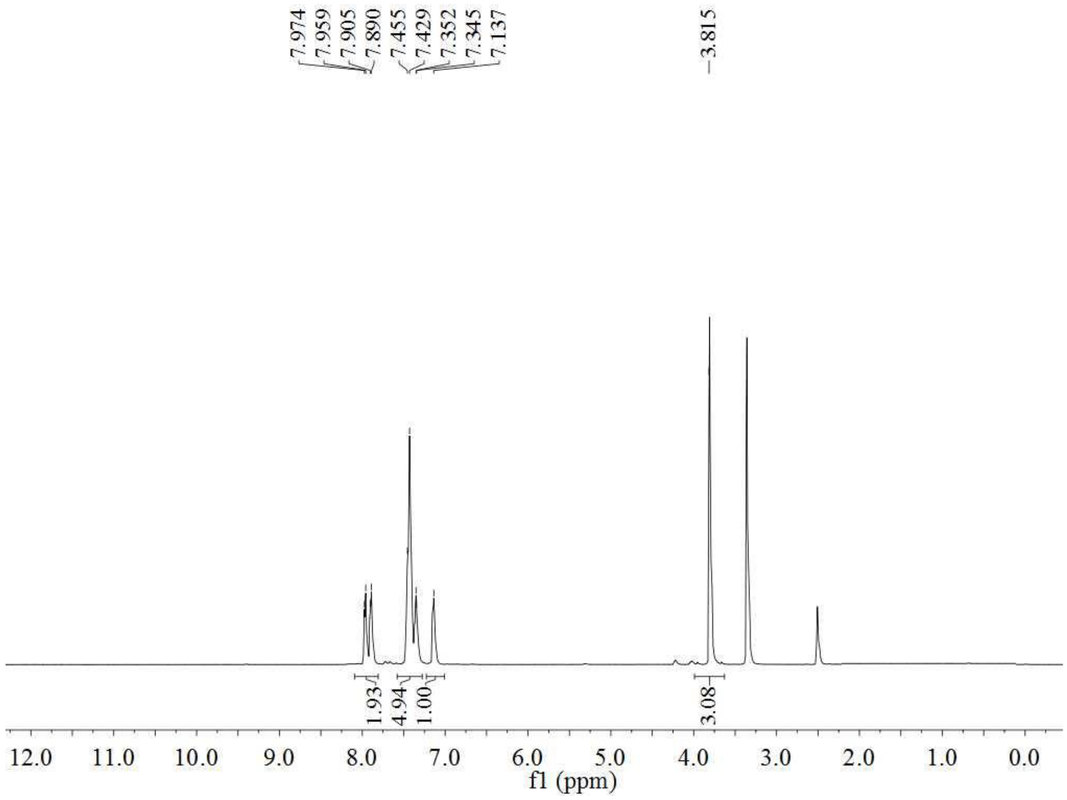

Figure 9. **4e** ^{1}H NMR

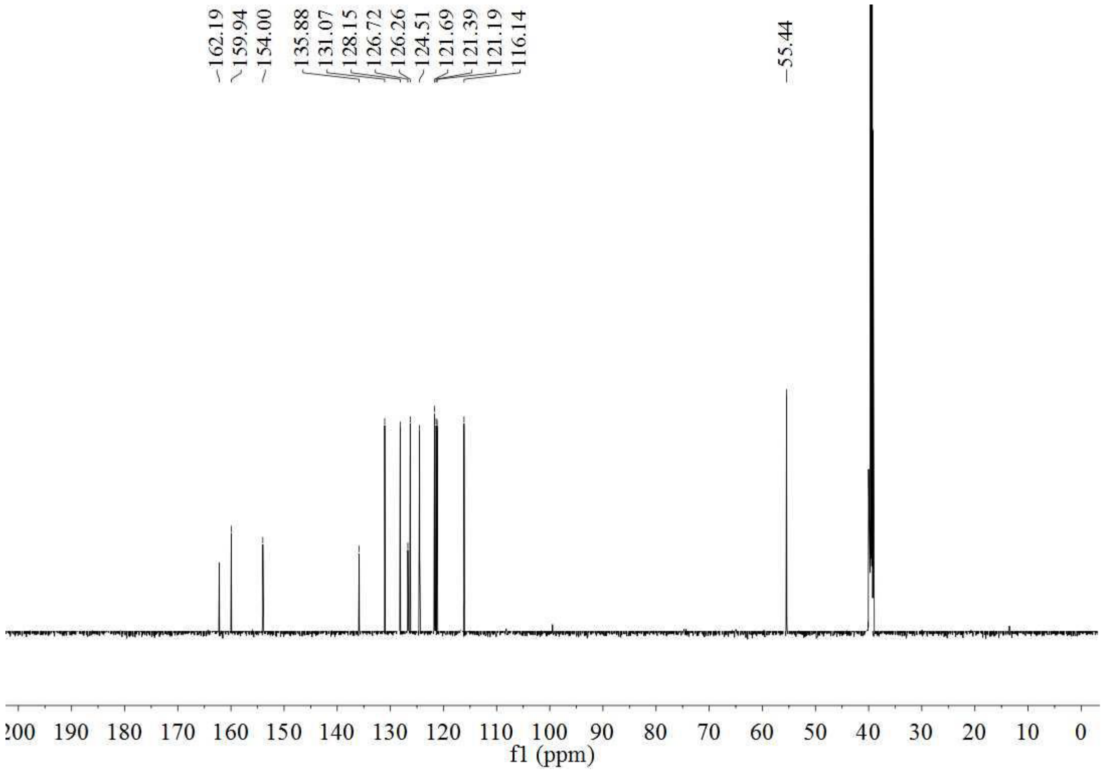

Figure 10. **4e** ^{13}C NMR

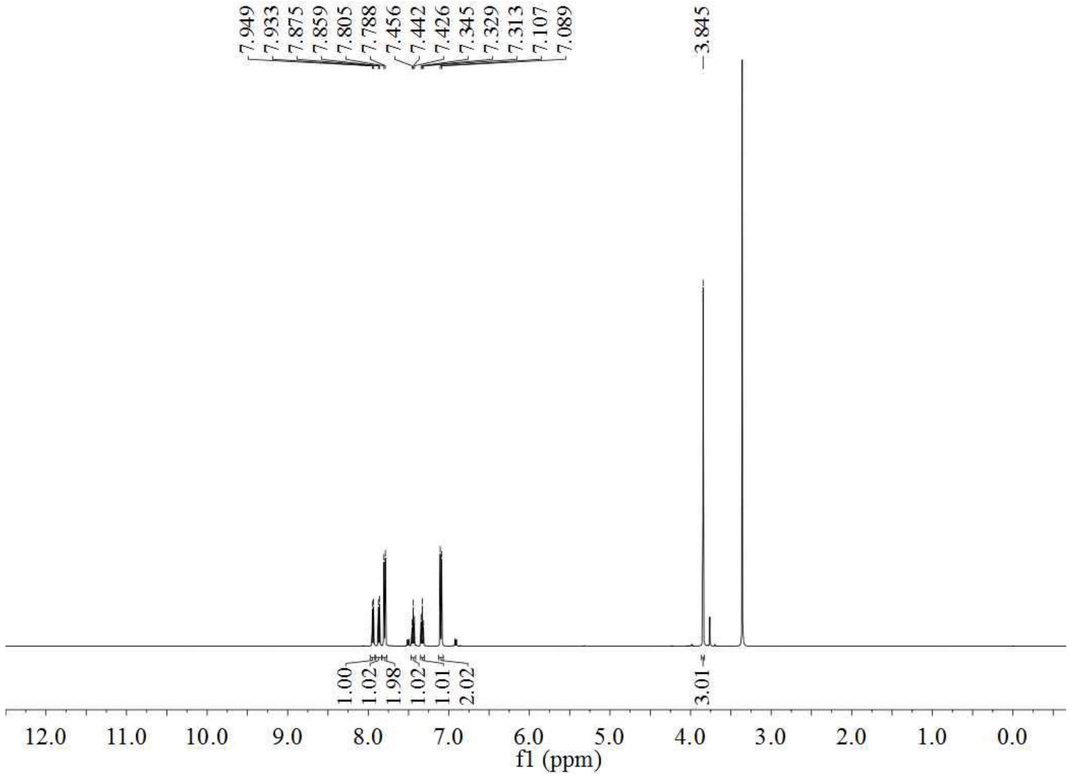

Figure 11. **4f** ^{1}H NMR

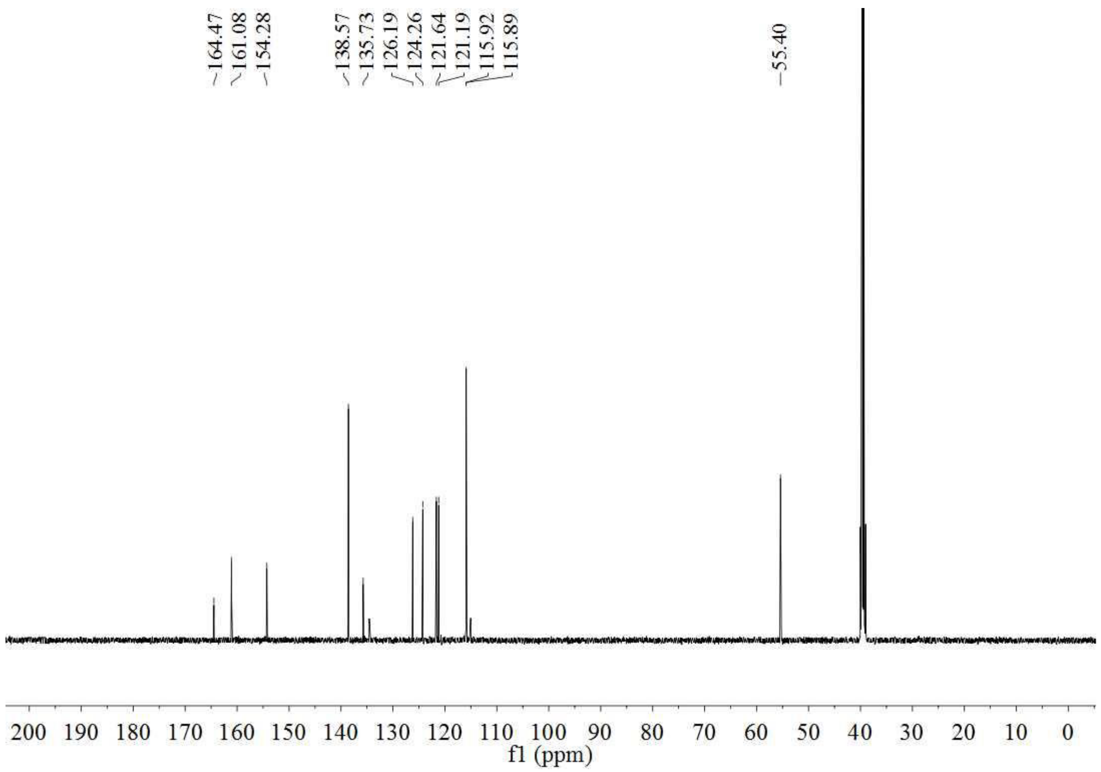

Figure 12. **3f** ^{13}C NMR

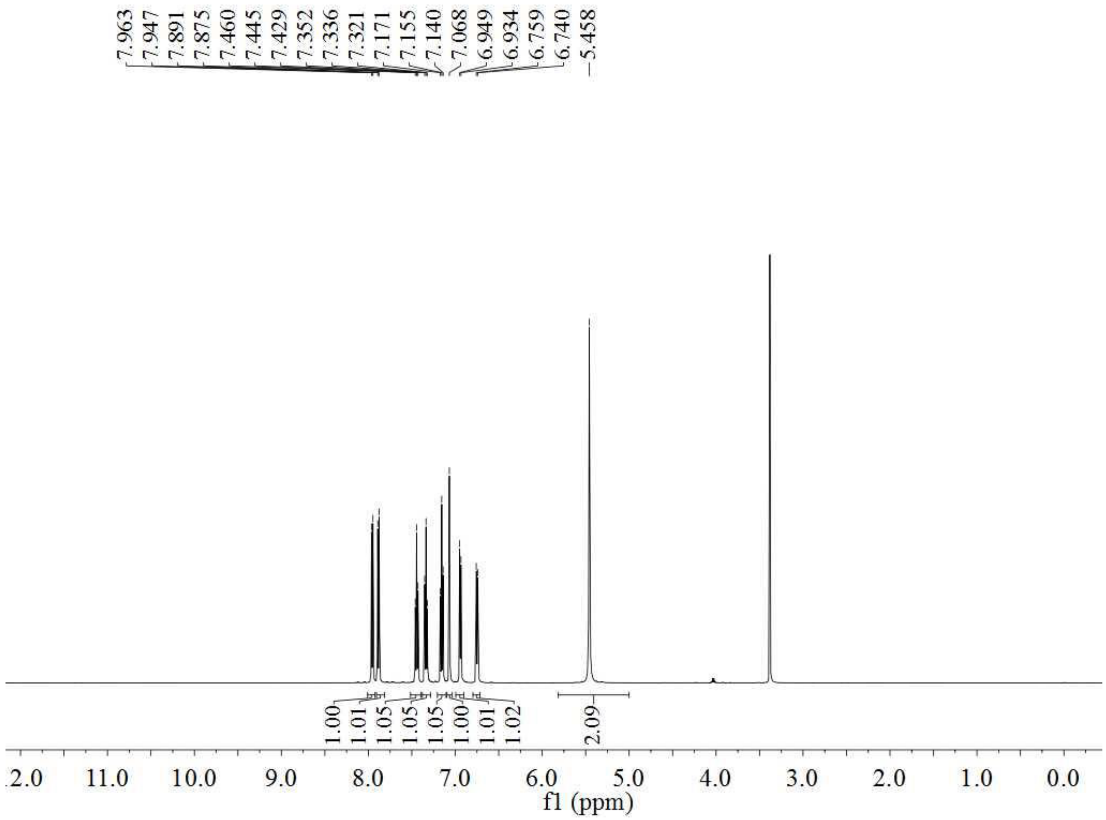

Figure 13. **4g** ^{1}H NMR

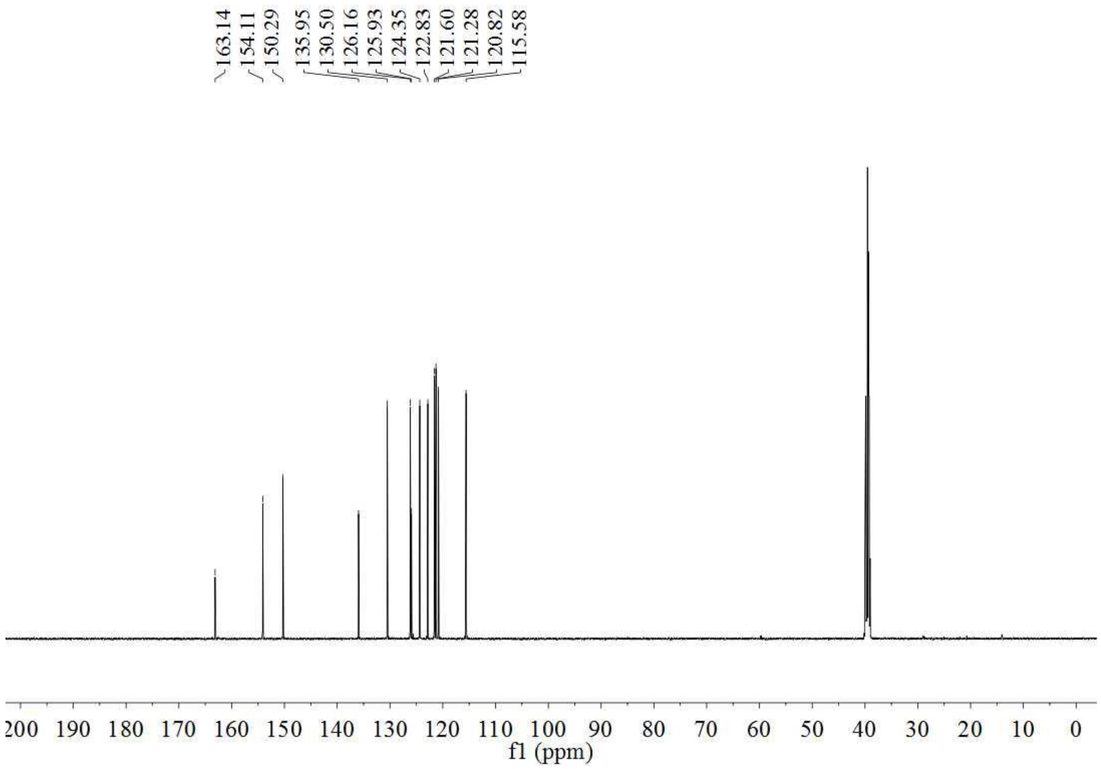

Figure 14. **4g** ^{13}C NMR

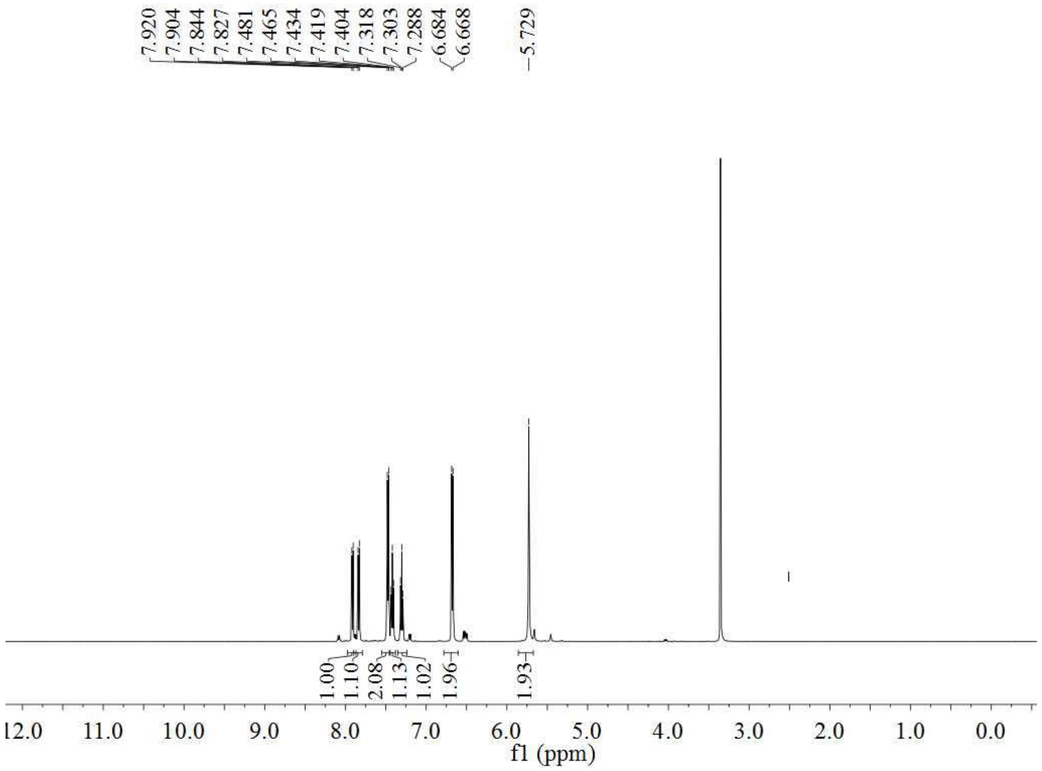

Figure 15. **4h** ^{1}H NMR

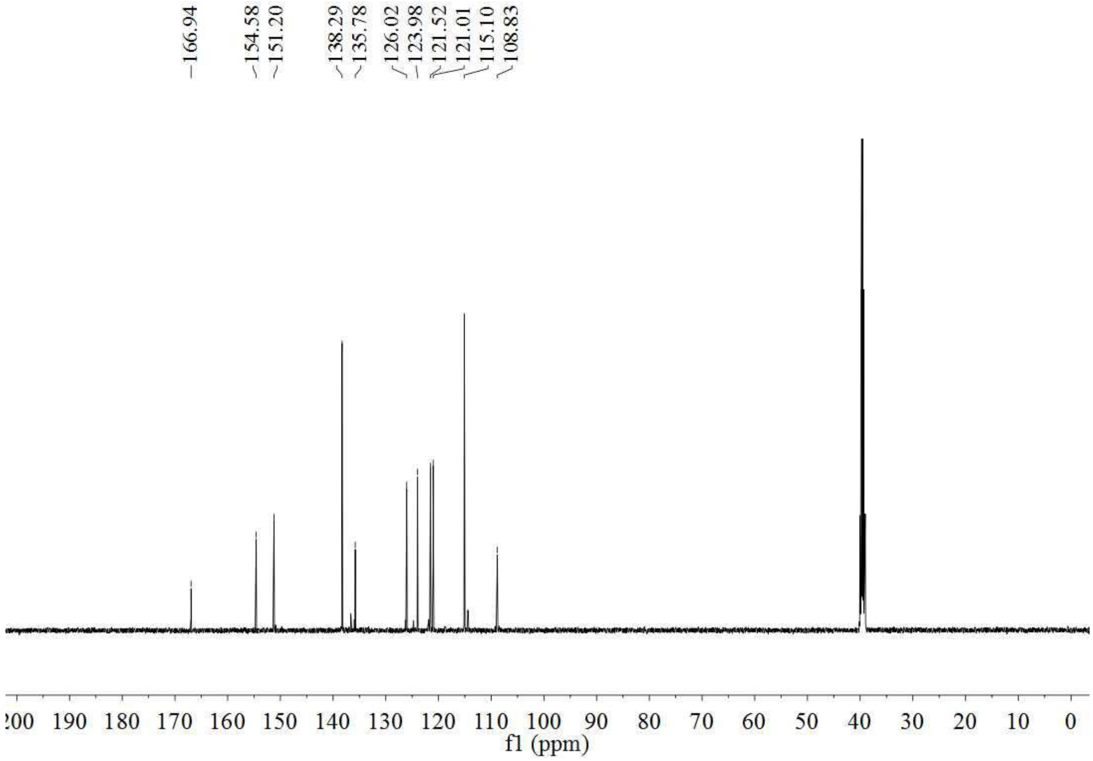

Figure 16. **4h** ^{13}C NMR

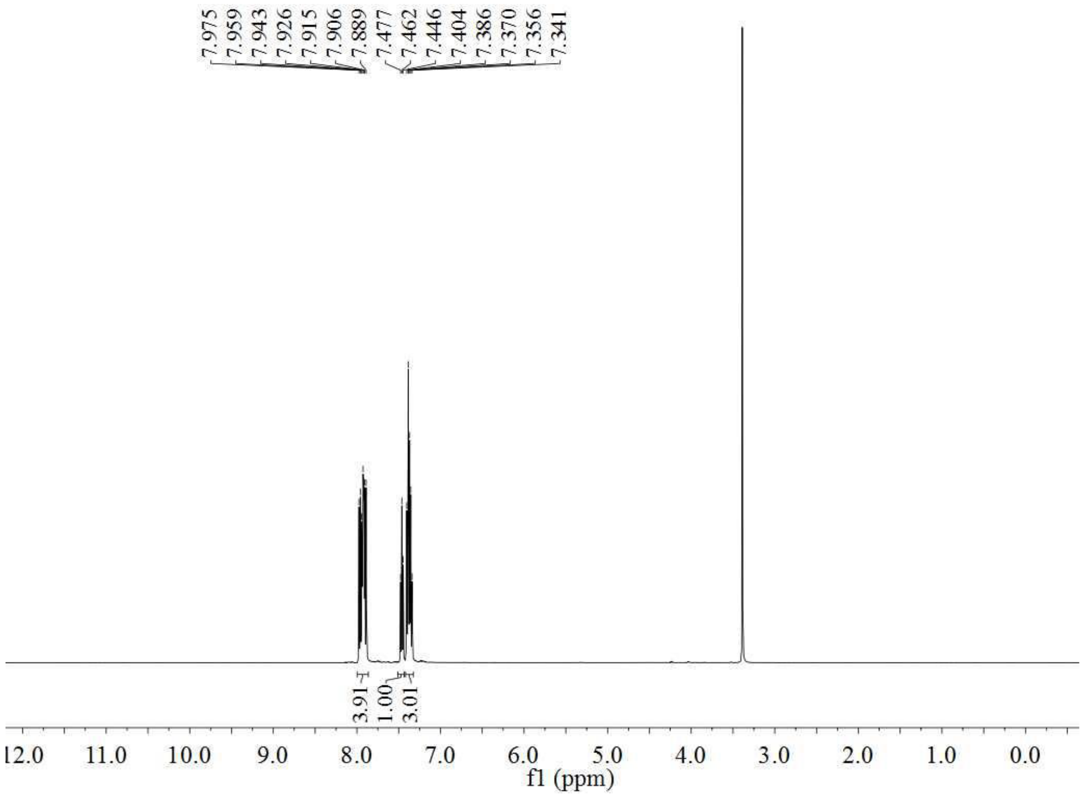

Figure 17. **4i** ^{1}H NMR

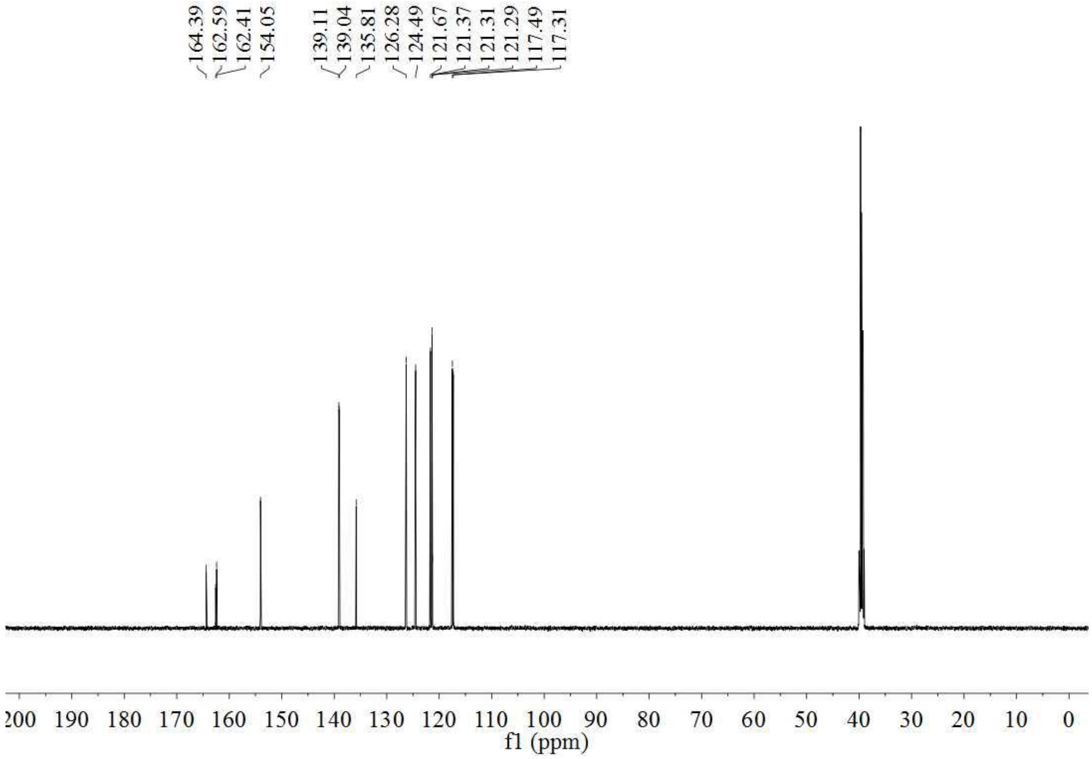

Figure 18. **4i** ^{13}C NMR

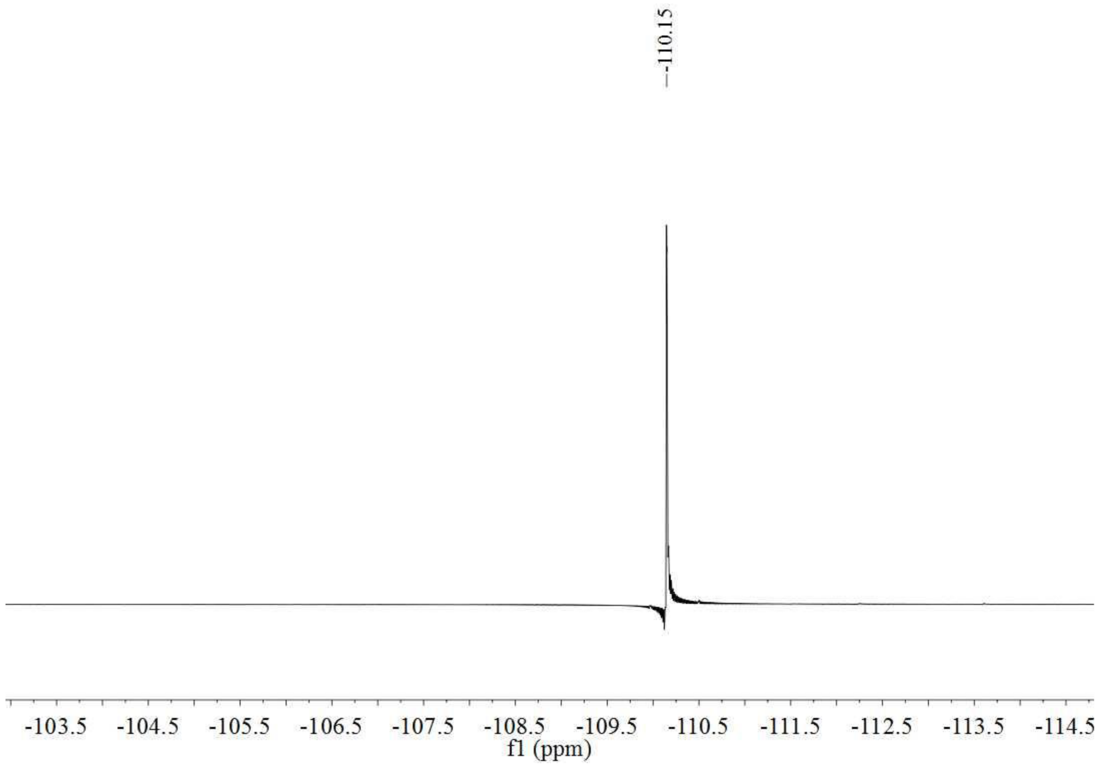

Figure 19. **4i** [19]f NMR

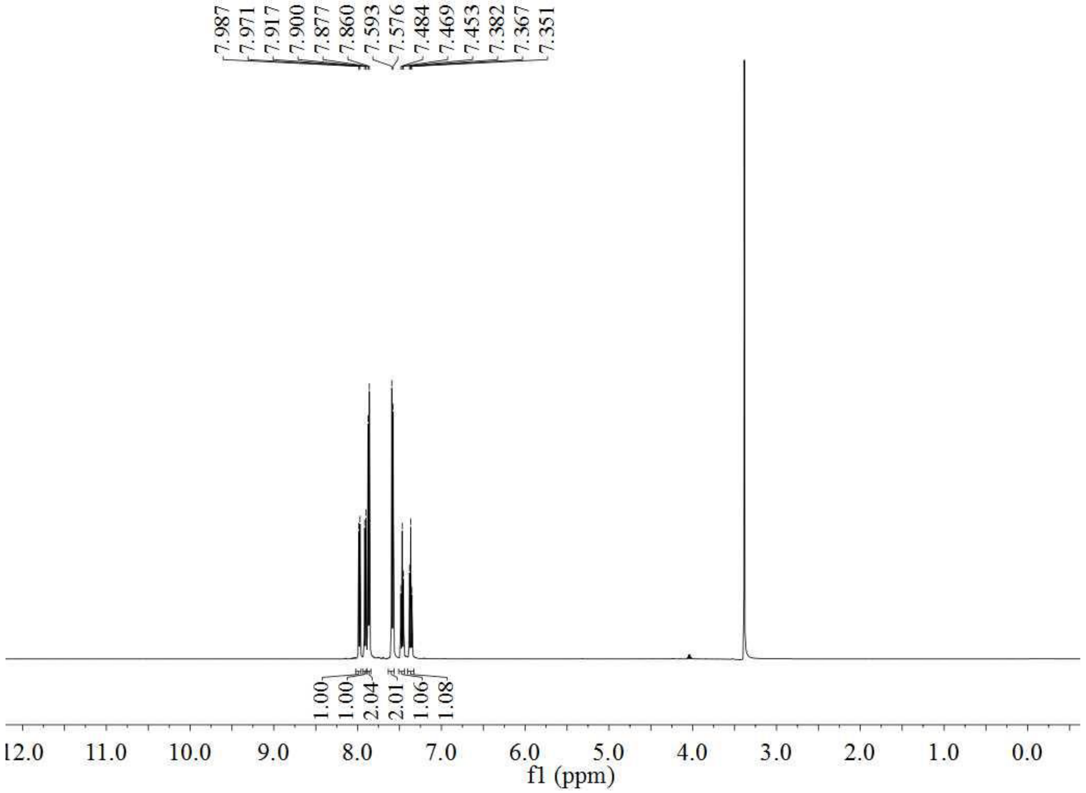

Figure 20. **4j** ^{1}H NMR

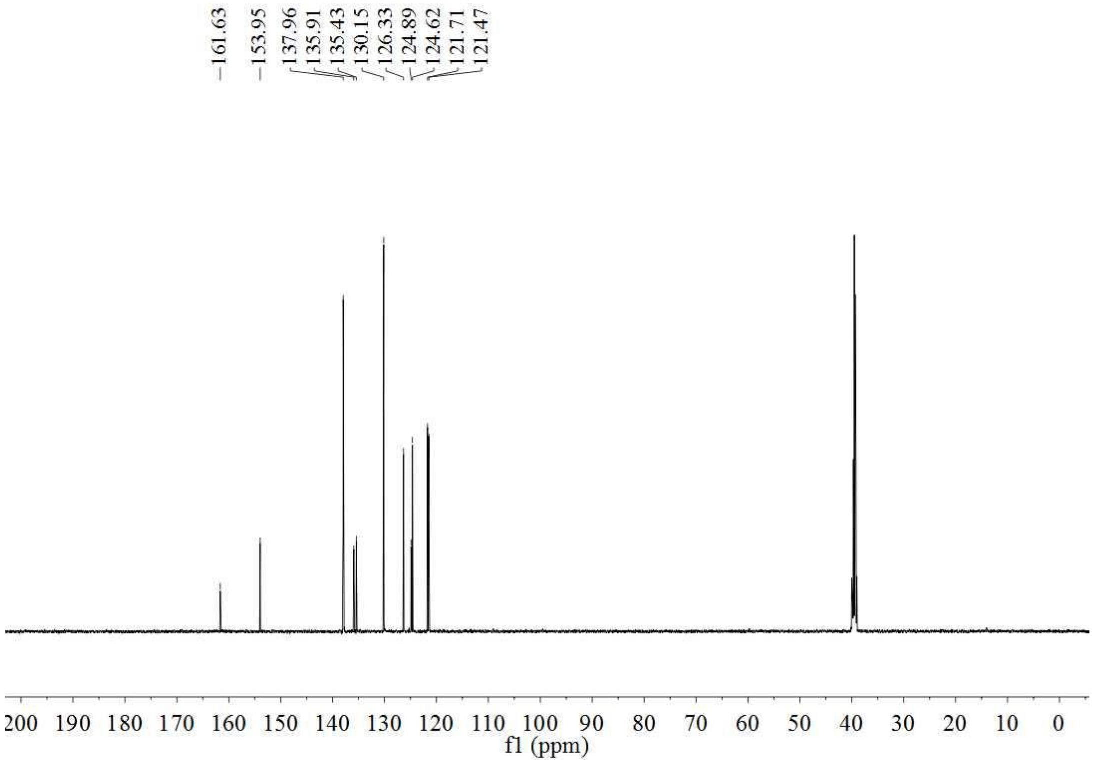

Figure 21. **4j** ^{13}C NMR

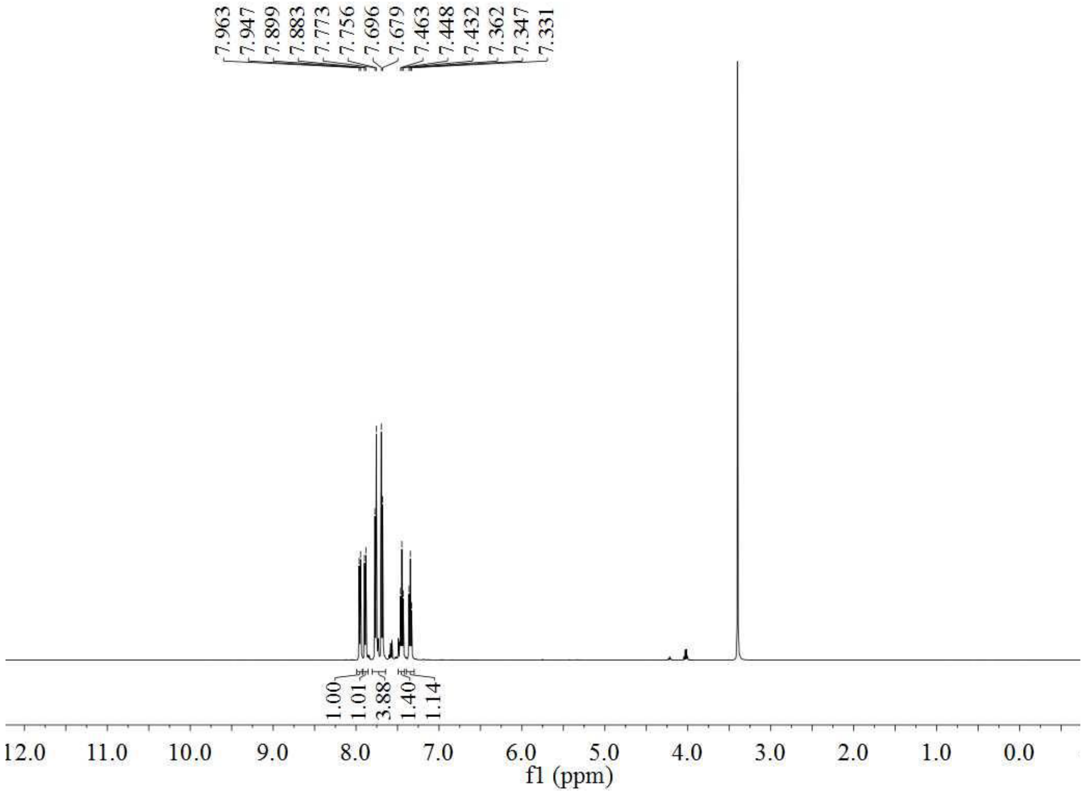

Figure 22. **4k** ^{1}H NMR

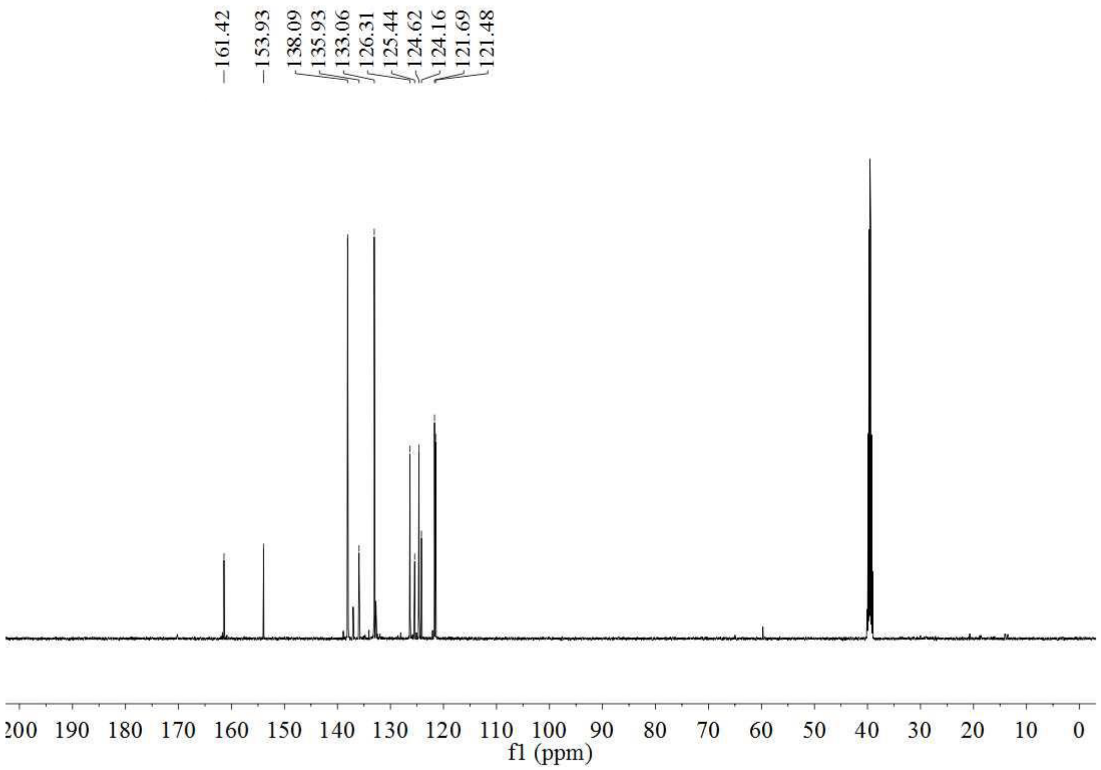

Figure 23. **4k** ^{13}C NMR

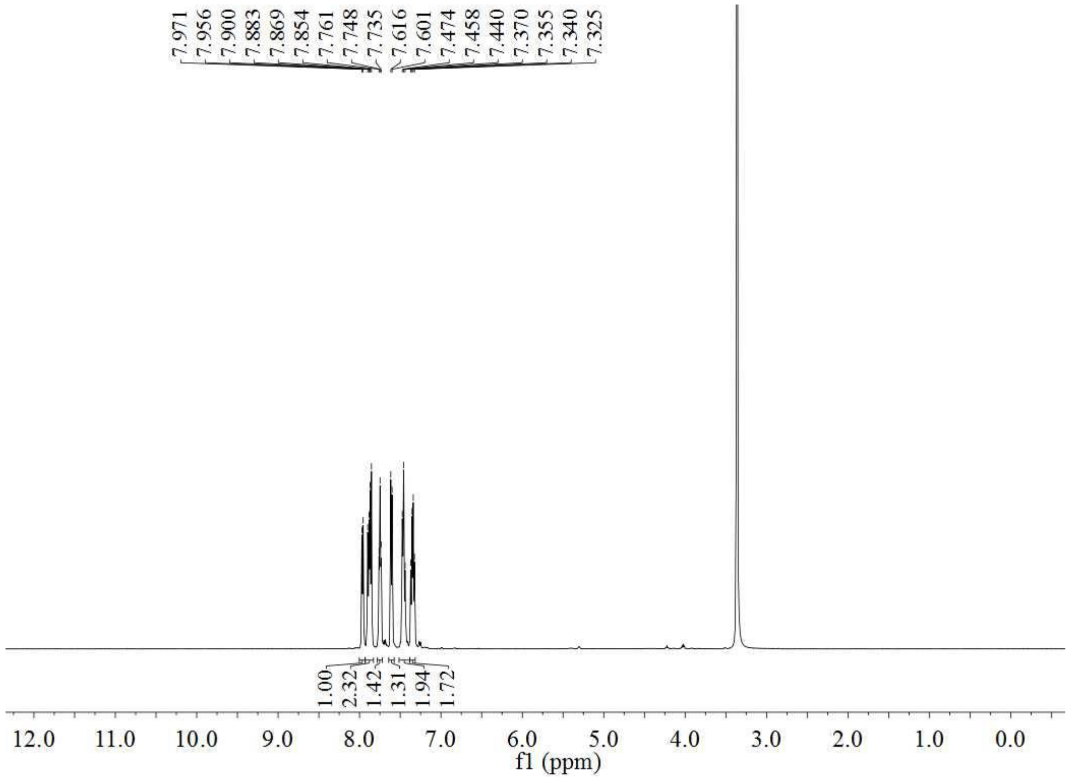

Figure 24. **4l** ^{1}H NMR

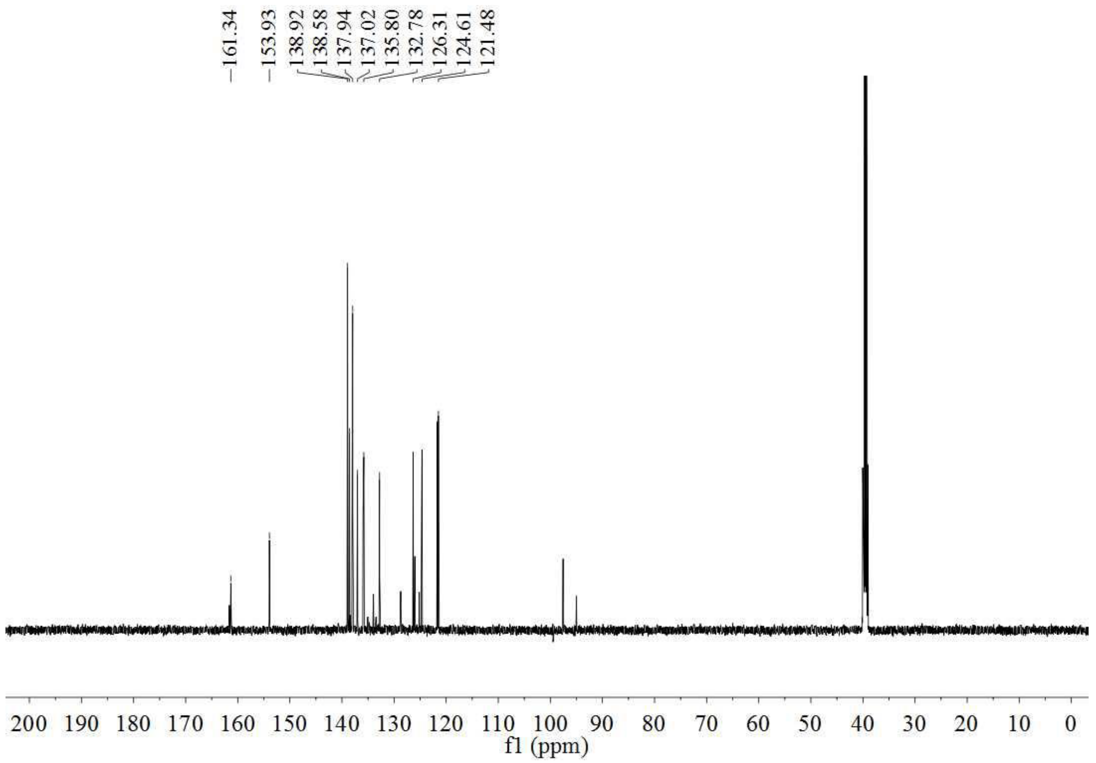

Figure 25. **4l** ^{13}C NMR

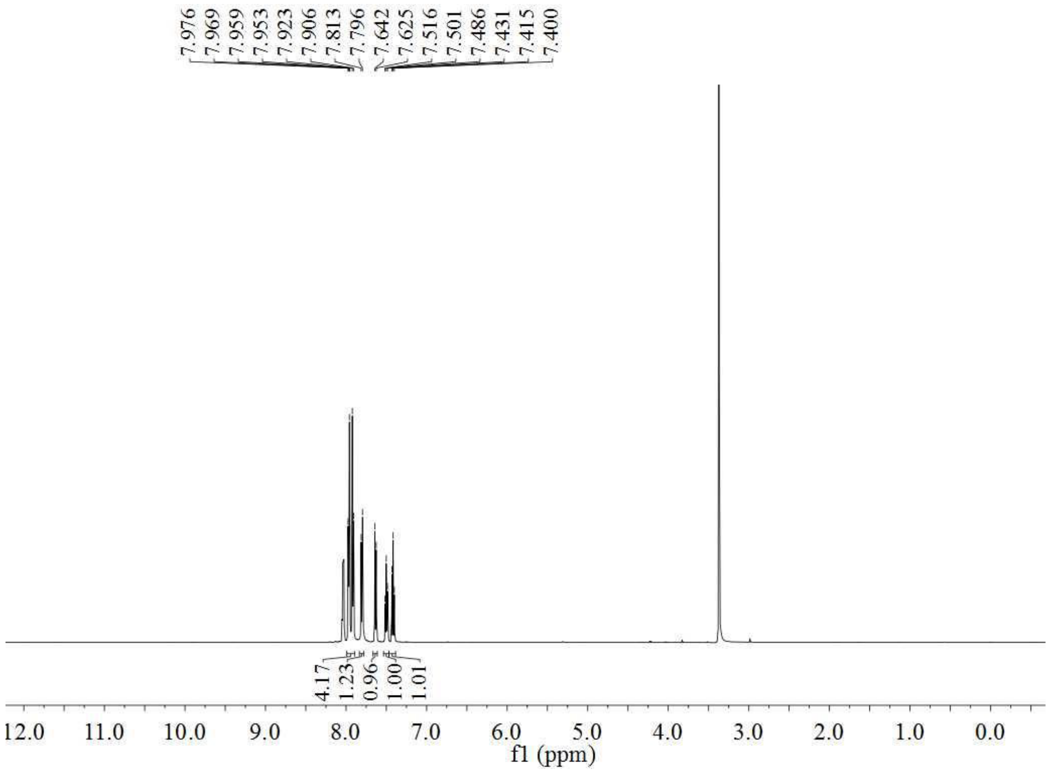

Figure 26. **4m** ^{1}H NMR

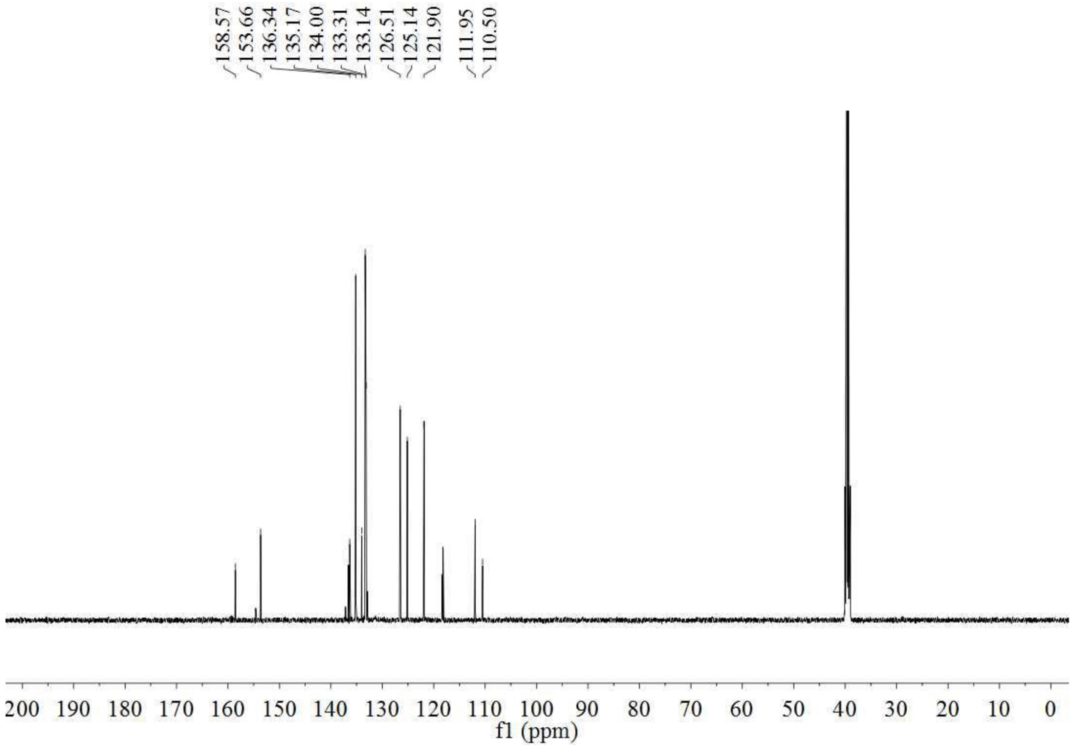

Figure 27. **4m** ^{13}C NMR

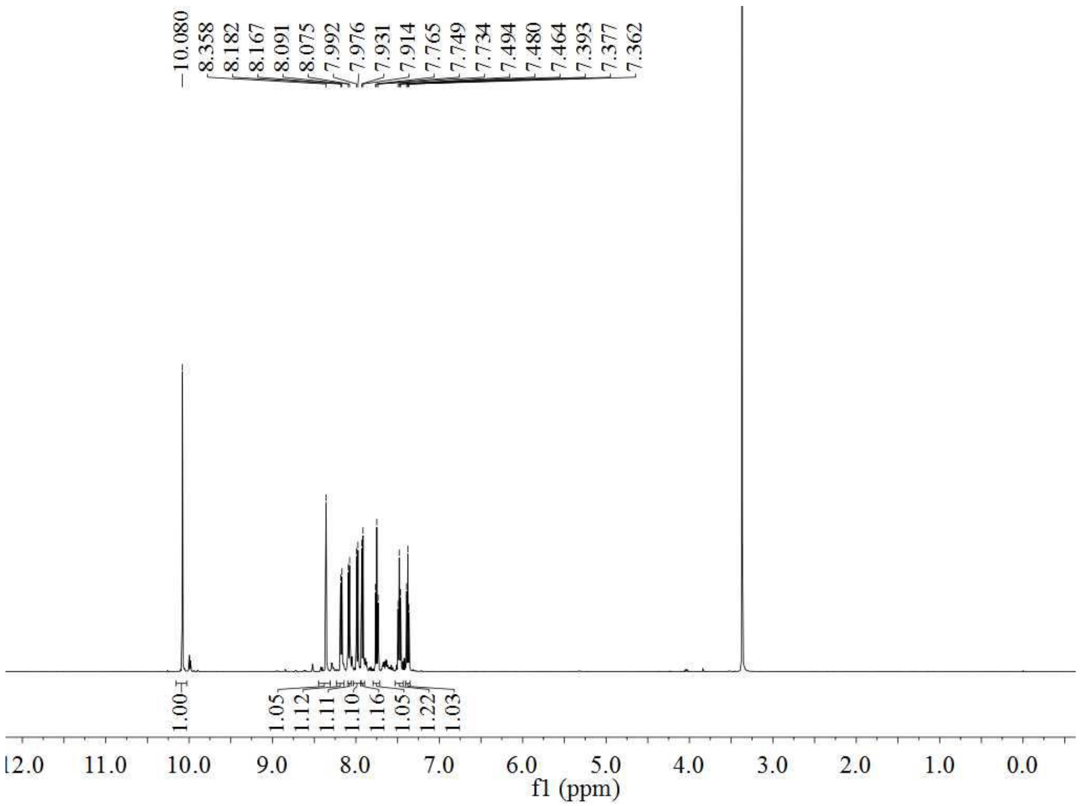

Figure 28. **4n** ^{1}H NMR

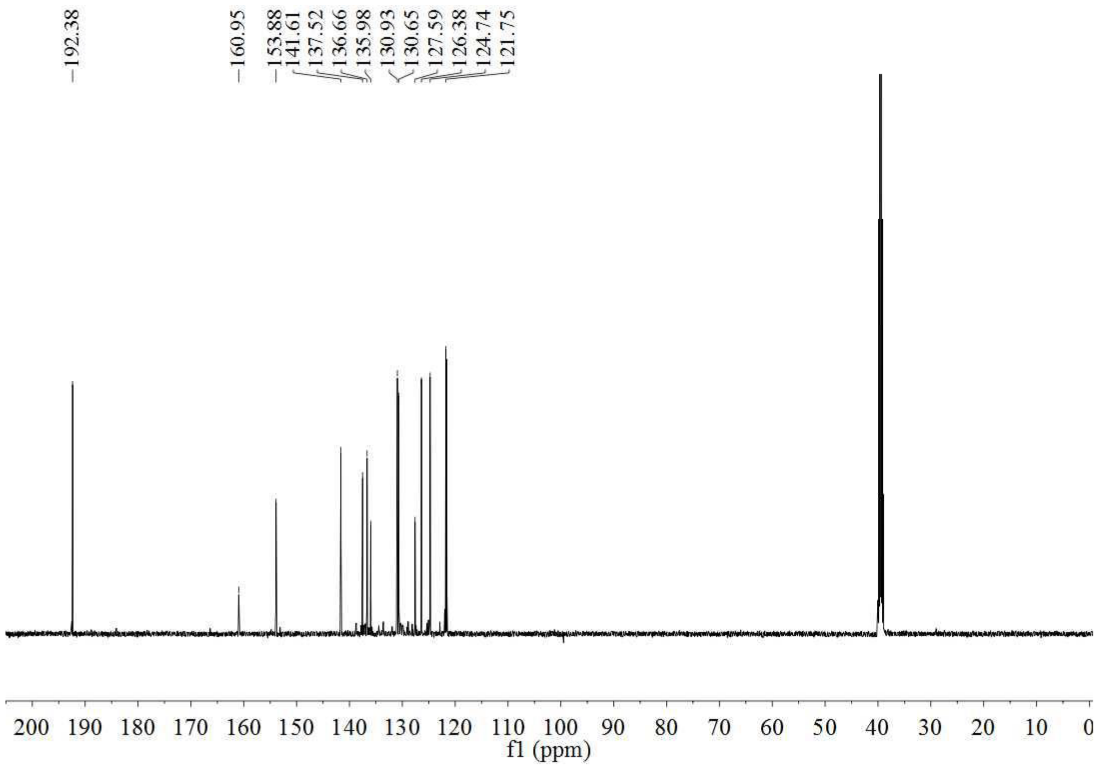

Figure 29. **4n** ^{13}C NMR

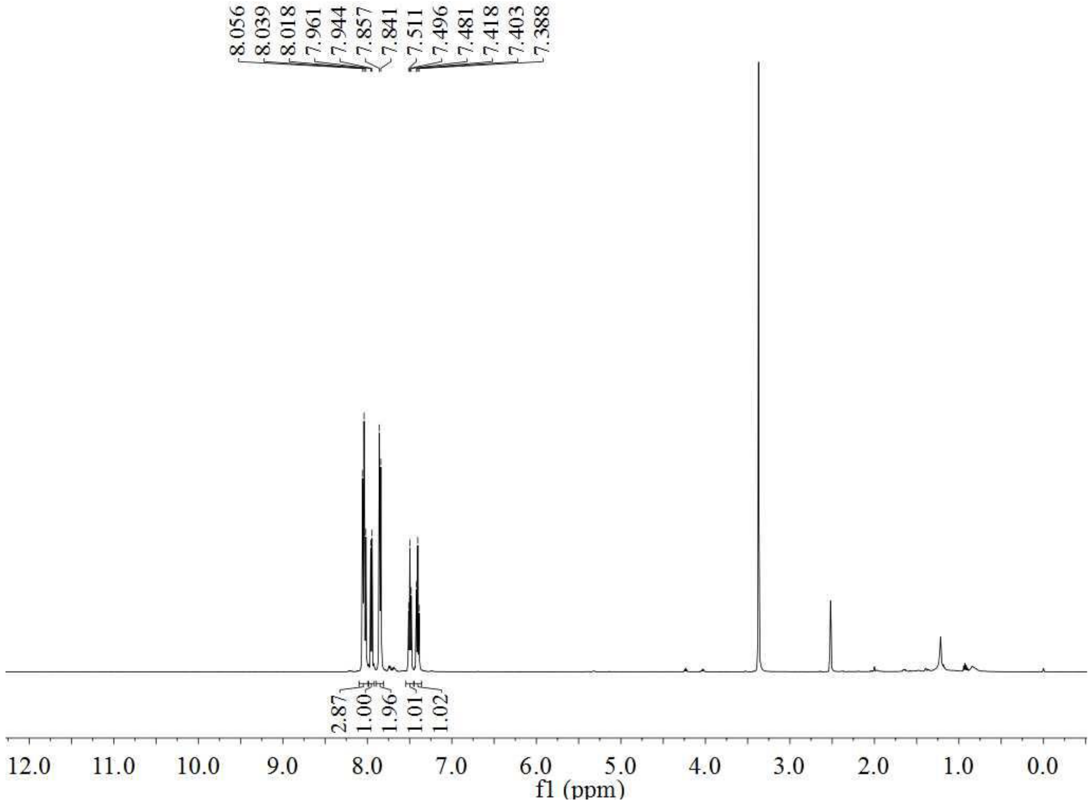

Figure 30. **4o** ^{1}H NMR

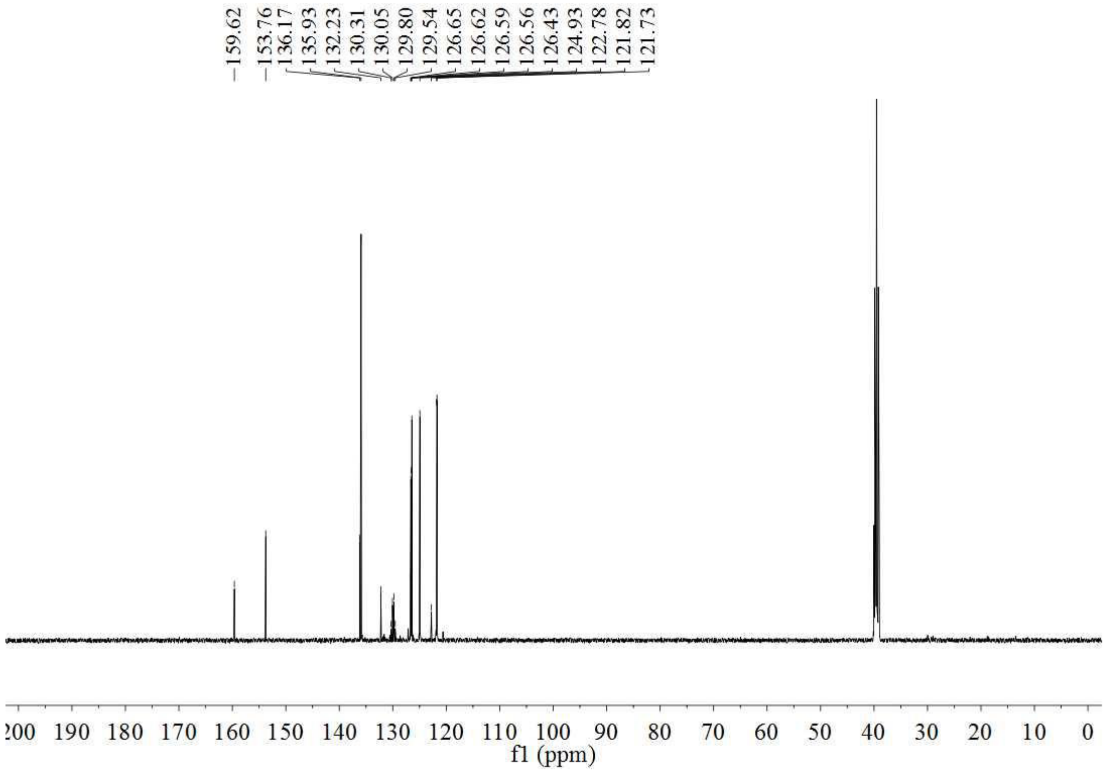

Figure 31. **4o** ^{13}C NMR

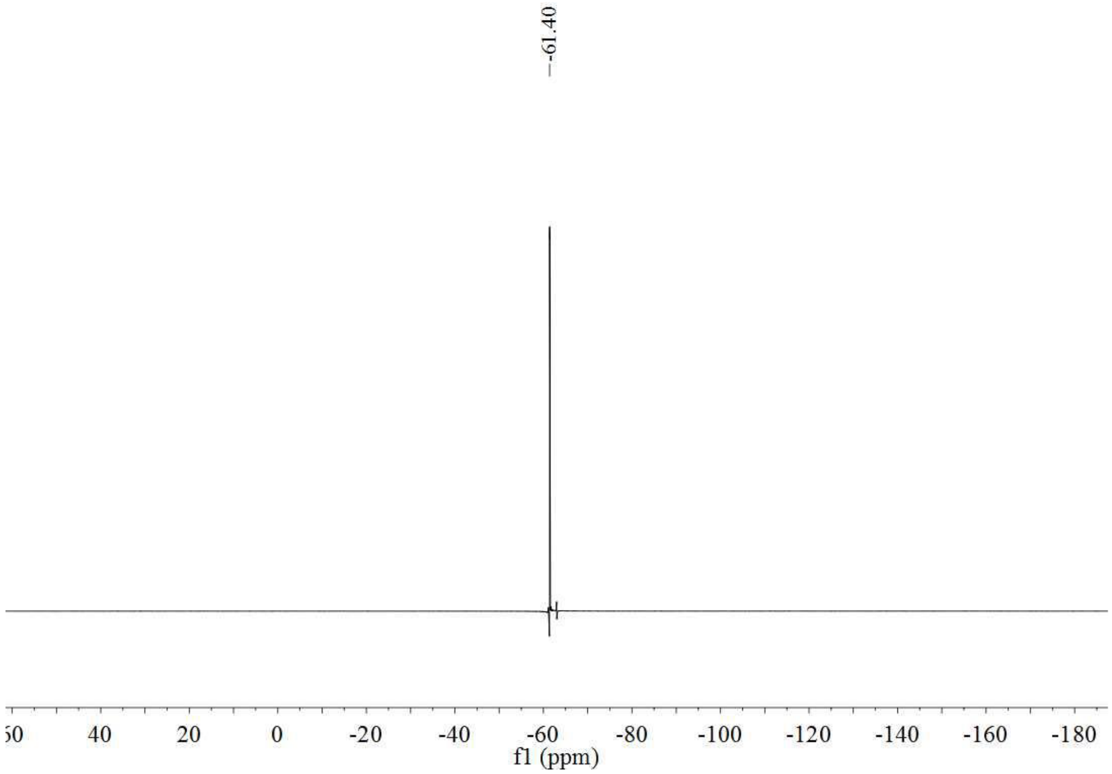

Figure 32. **4o** ^{19}F NMR

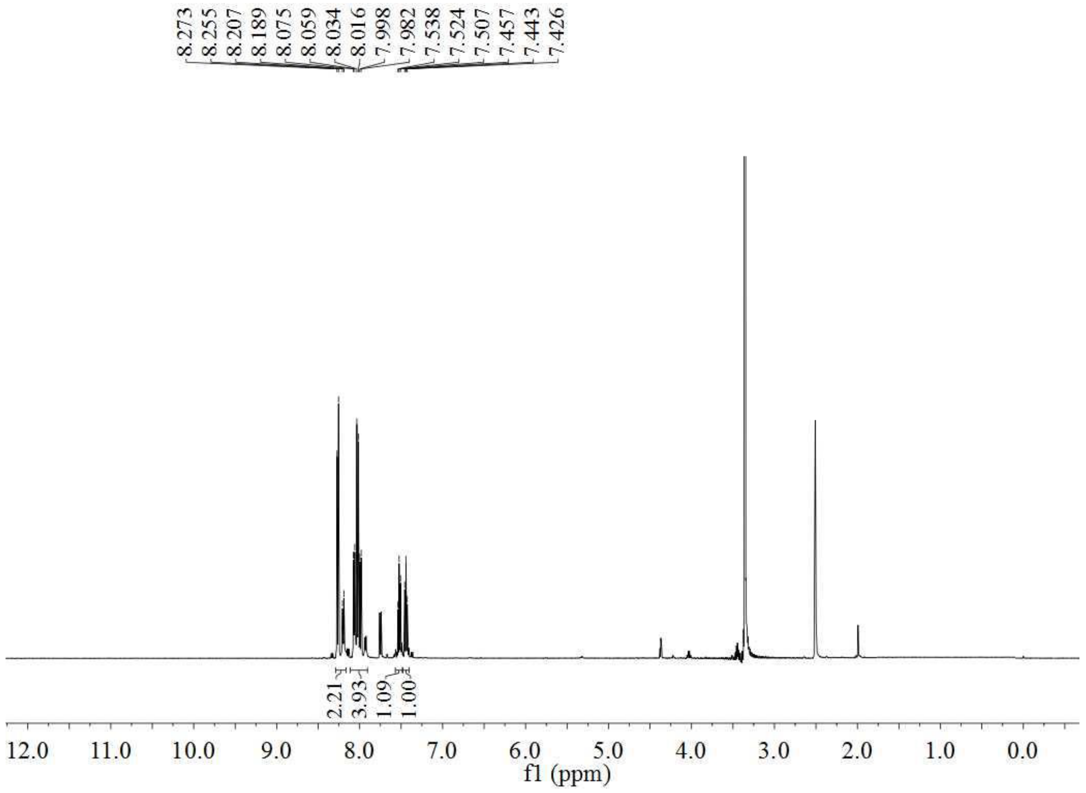

Figure 33. **4p** ^{1}H NMR

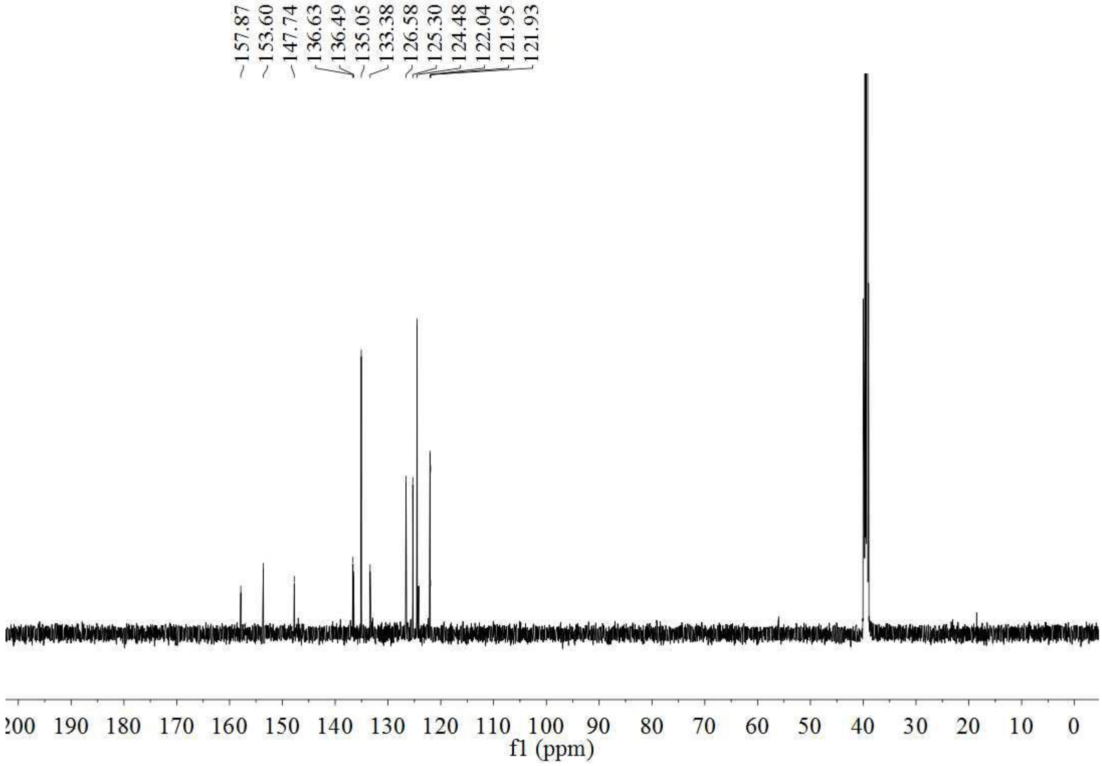

Figure 34. **4p** ^{13}C NMR

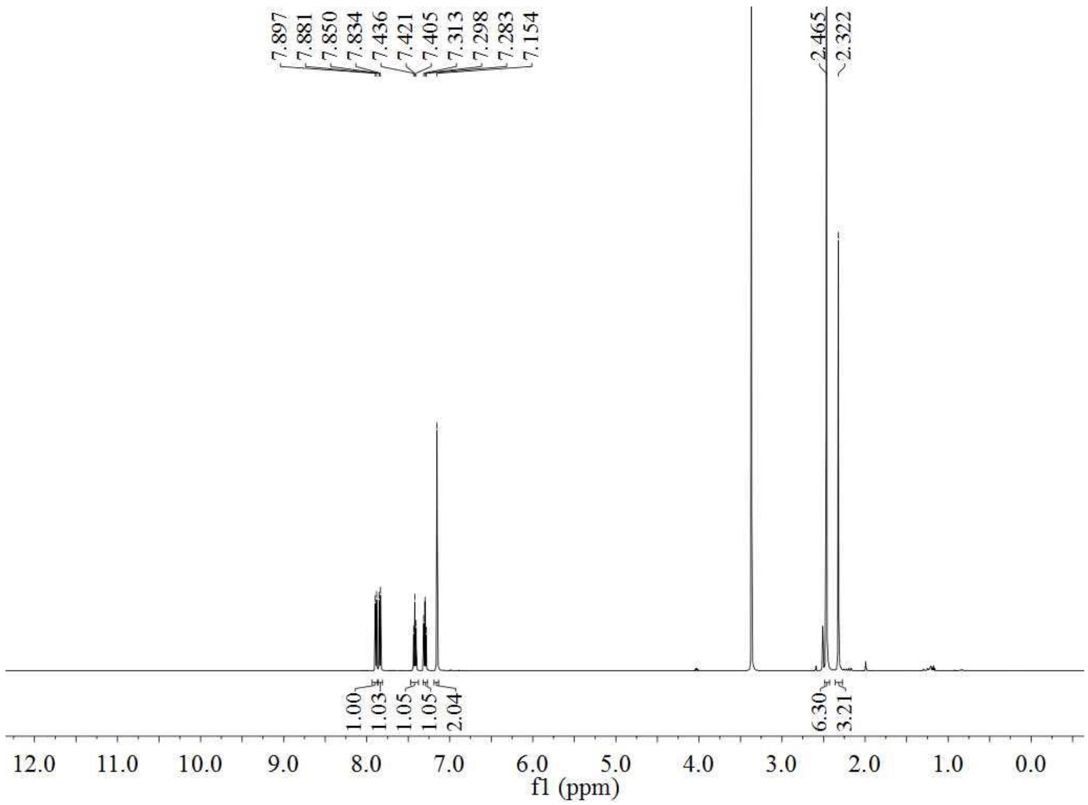

Figure 35. **4q** ^{1}H NMR

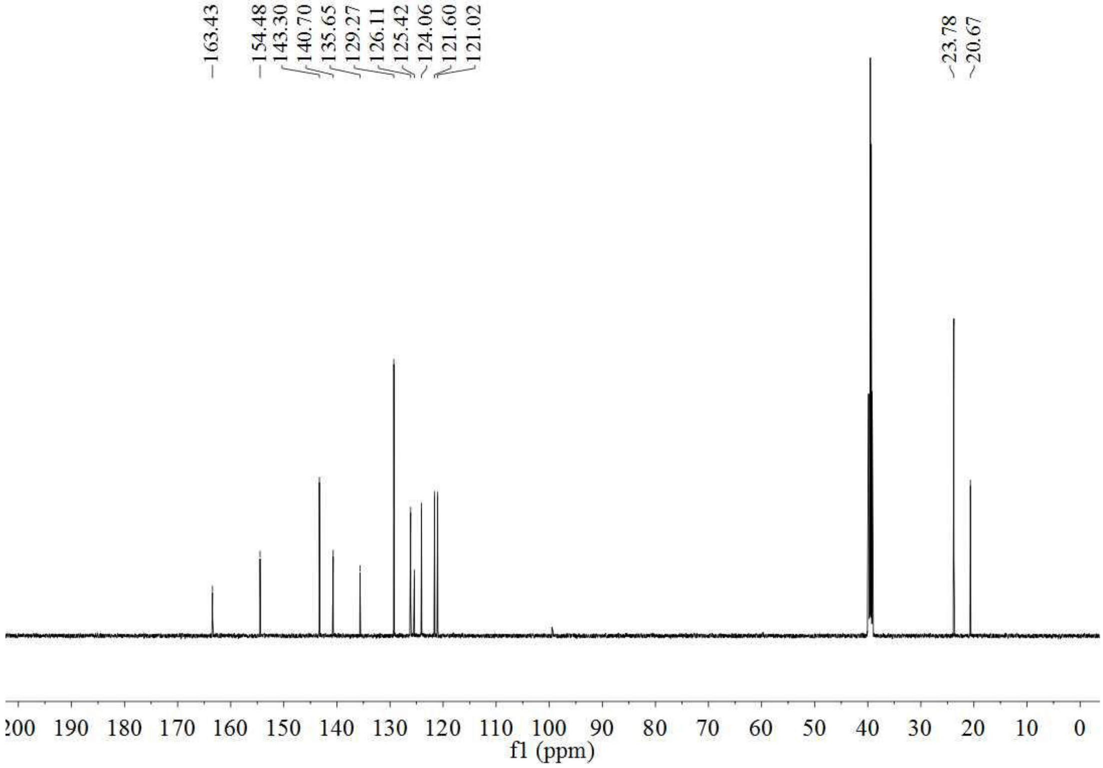

Figure 36. **4q** ^{13}C NMR

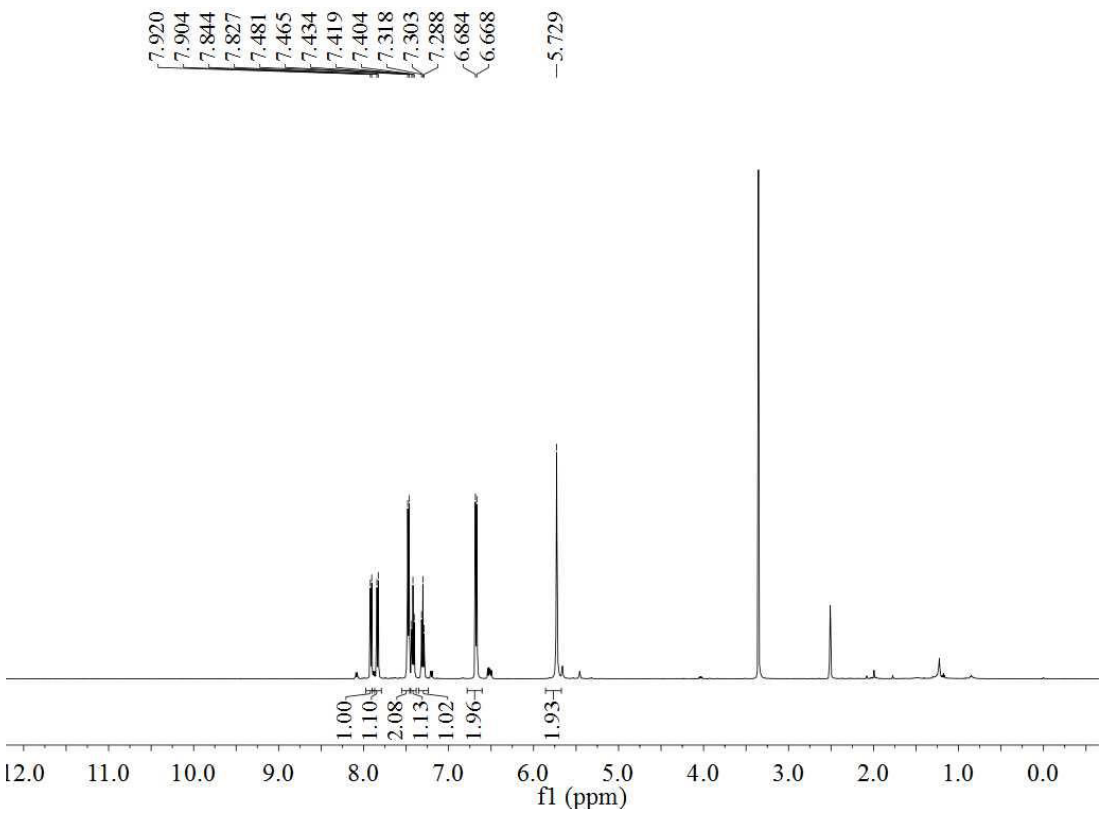

Figure 37. **4r**^{1}H NMR

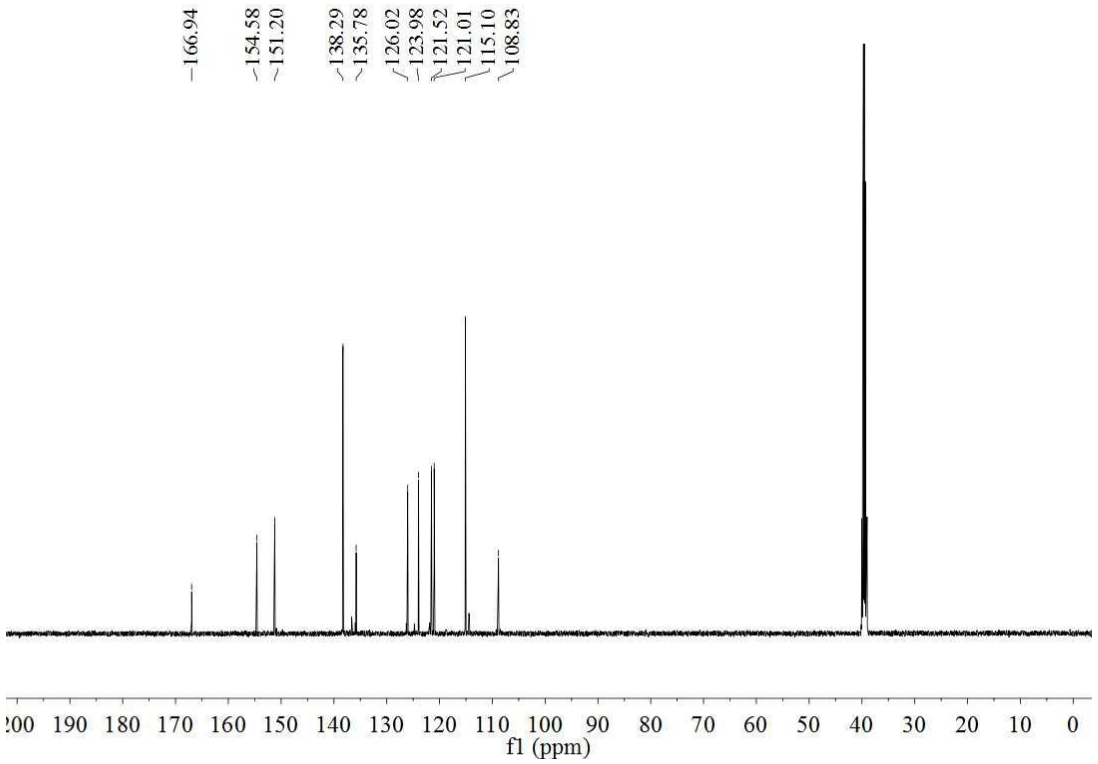

Figure 38. **4r** ^{13}C NMR

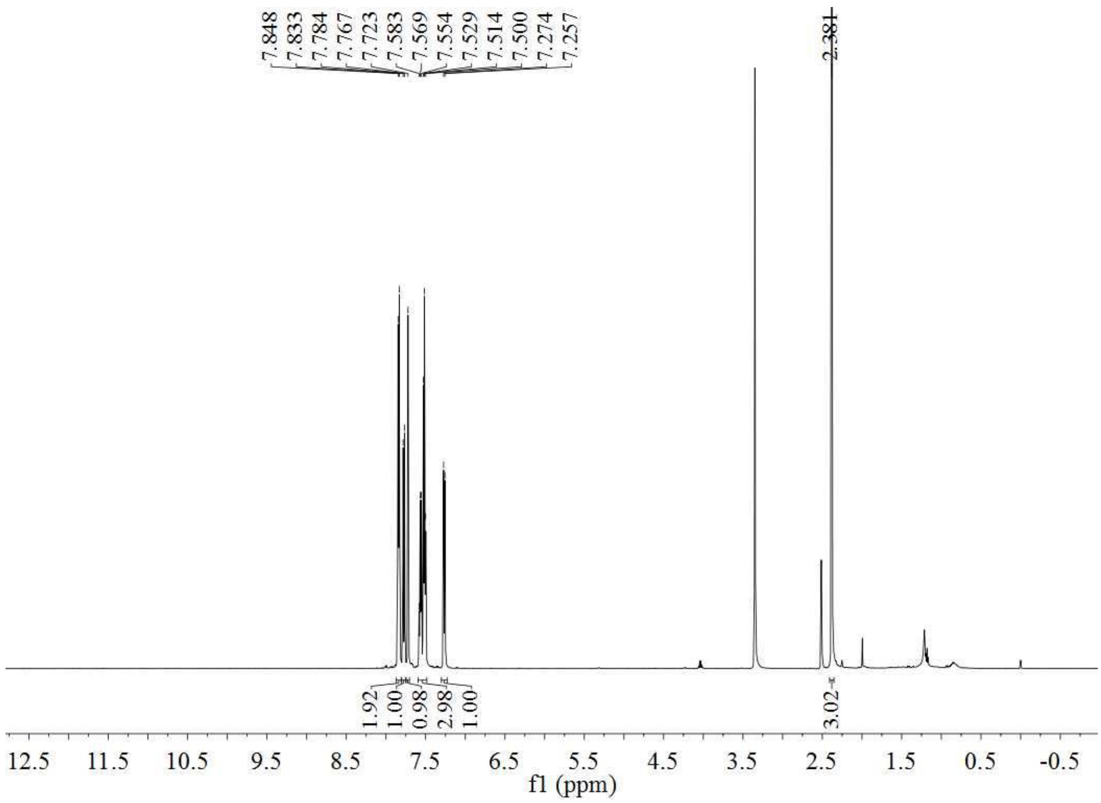

Figure 39. **7a** ^{1}H NMR

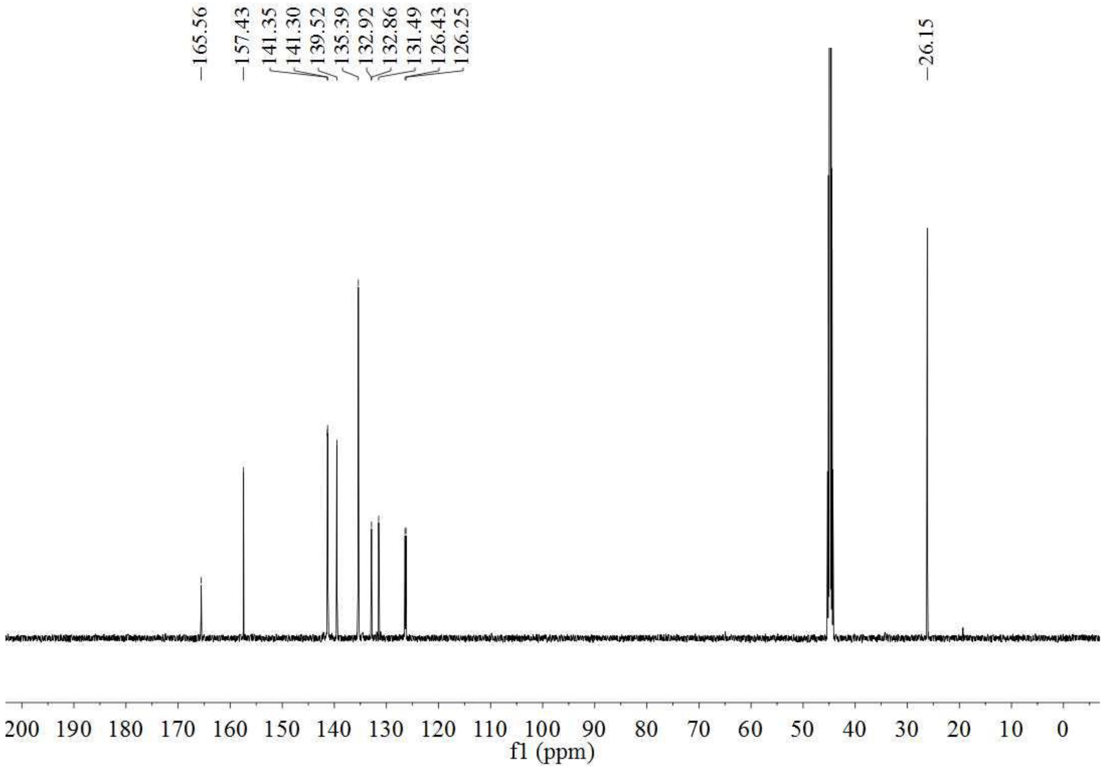

Figure 40. **7a** ^{13}C NMR

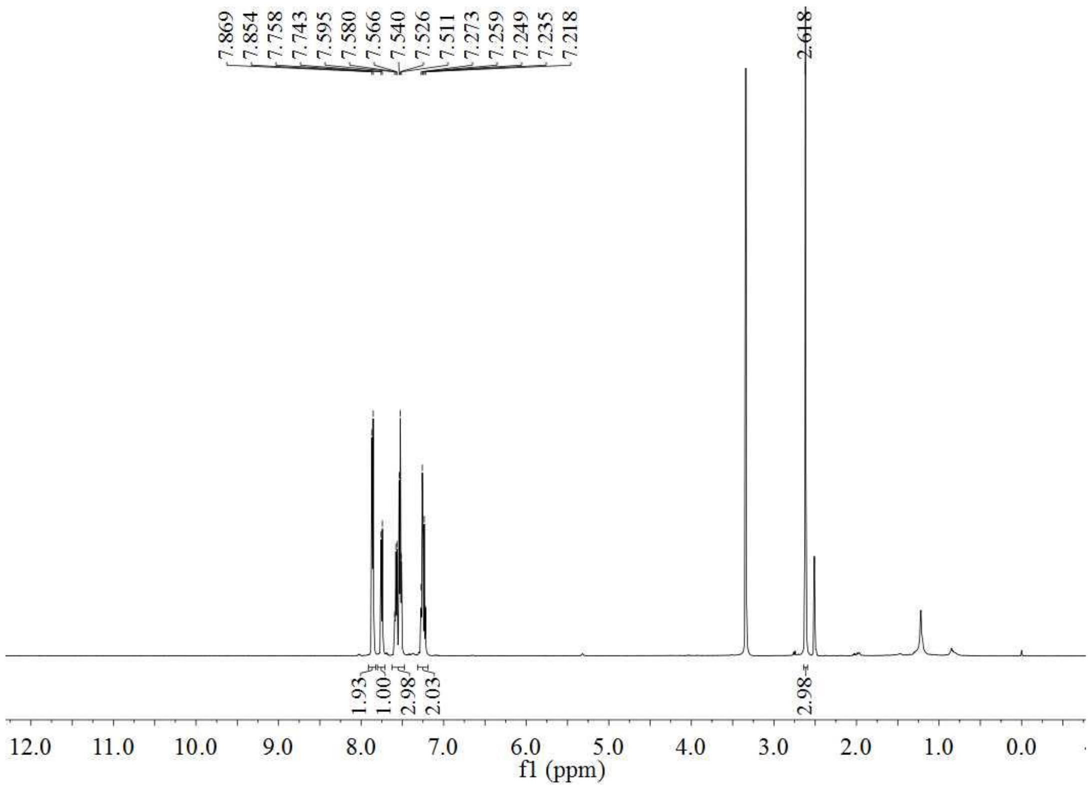

Figure 41. **7b** ^{1}H NMR

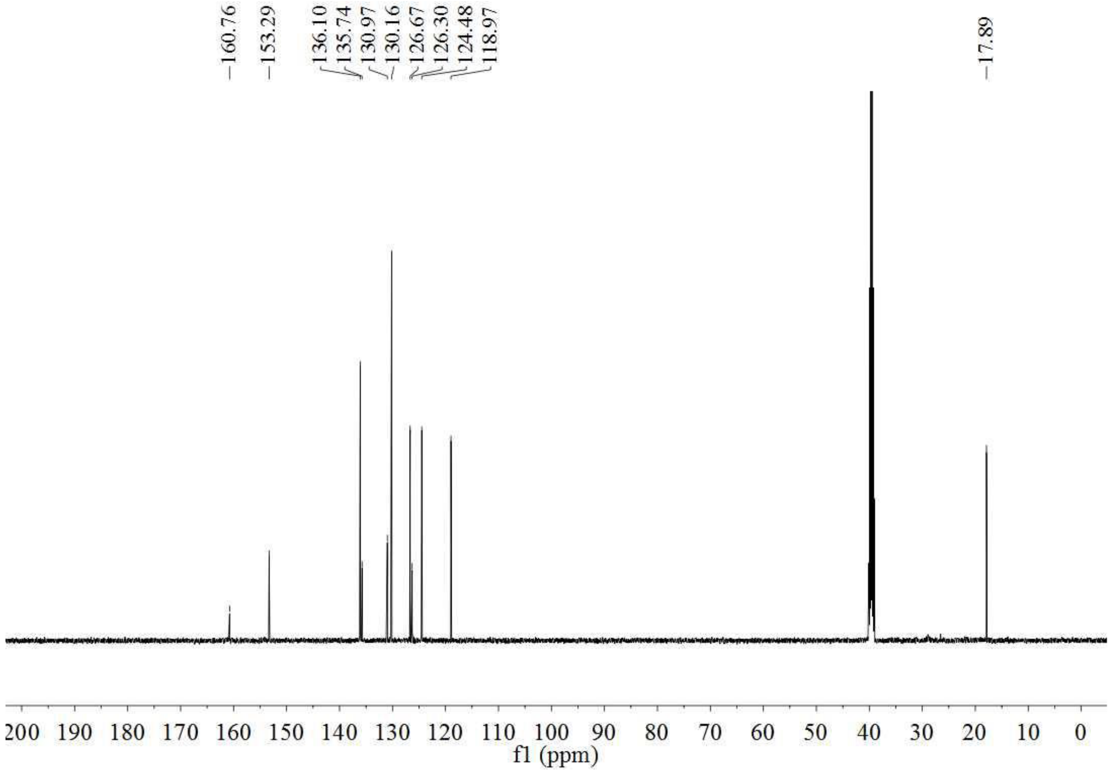

Figure 42. **7b** ^{13}C NMR

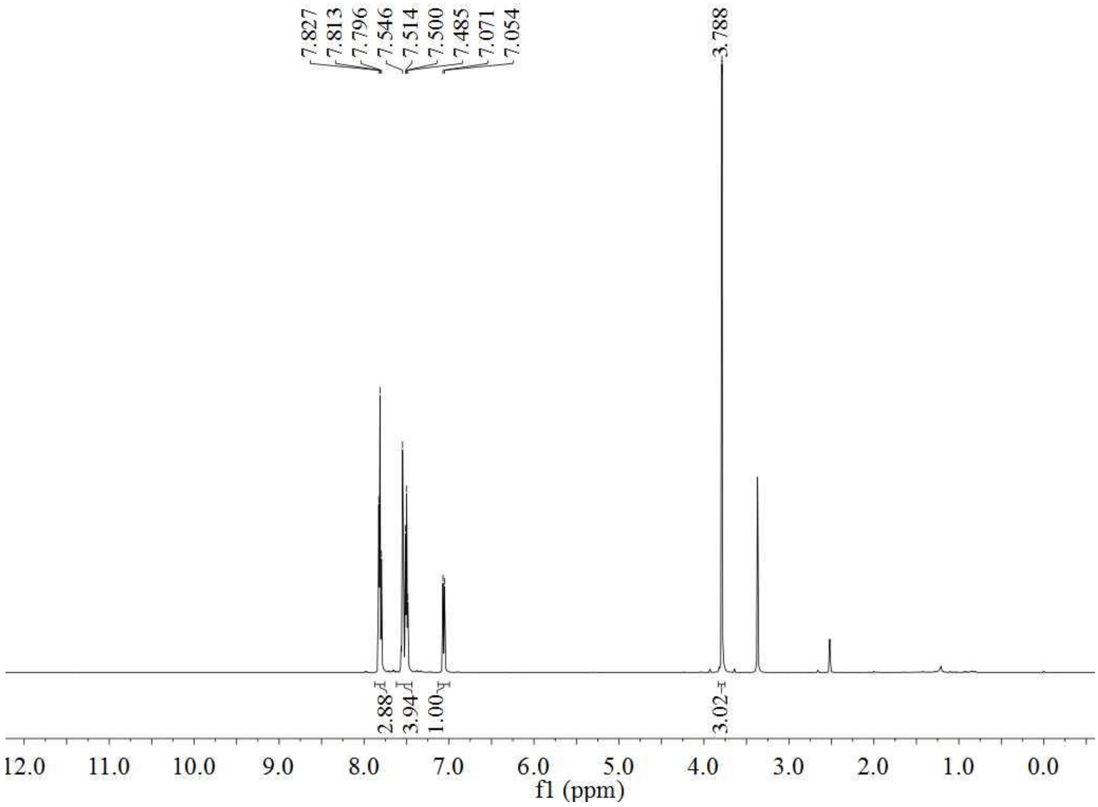

Figure 43. **7c** ^{1}H NMR

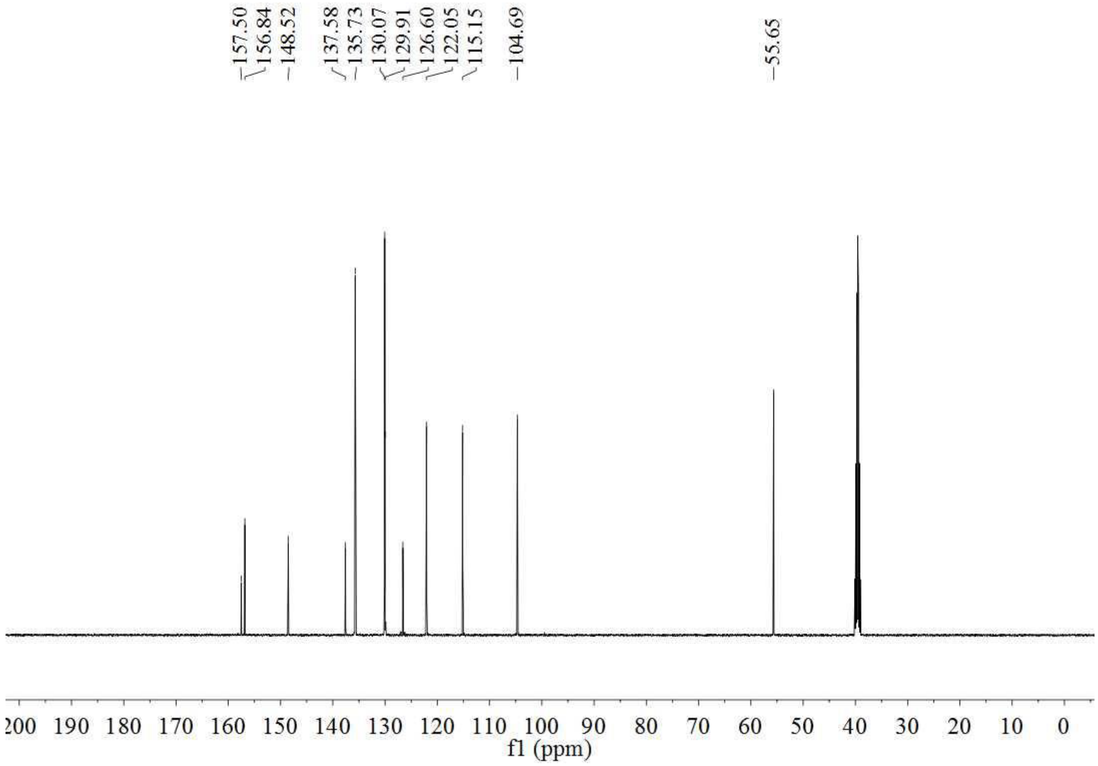

Figure 44. **7c** ^{13}C NMR

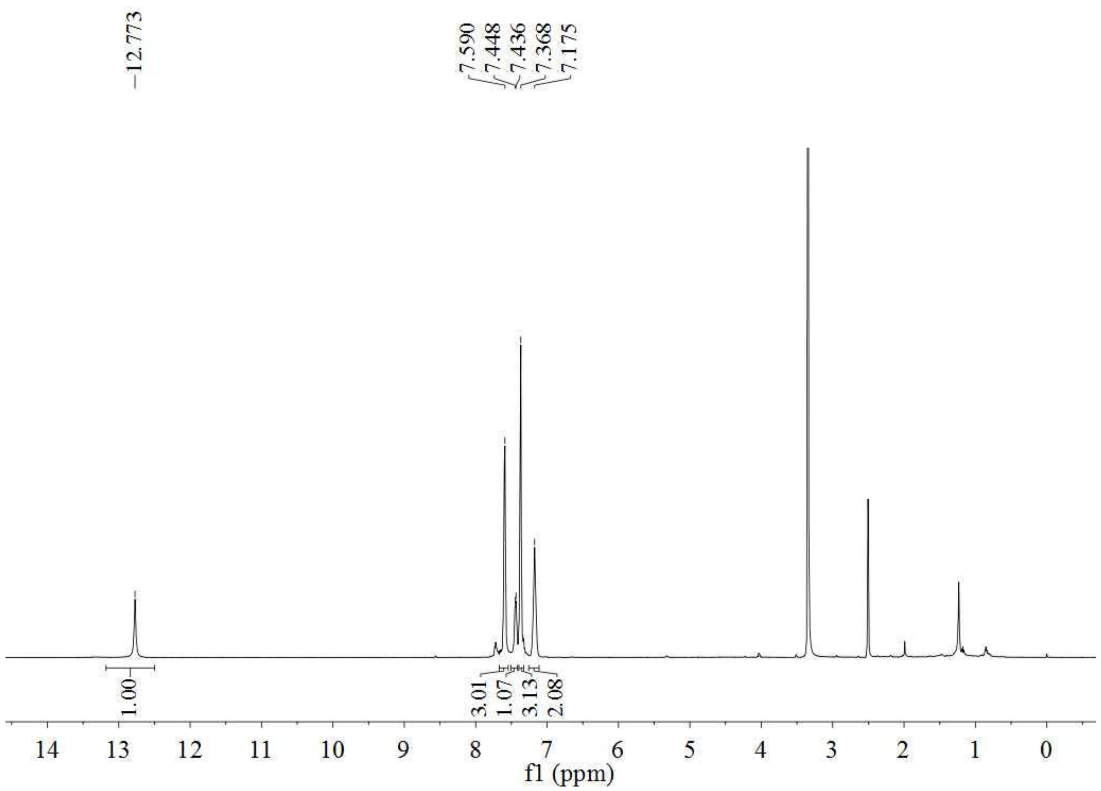

Figure 47. **9a** ^{1}H NMR

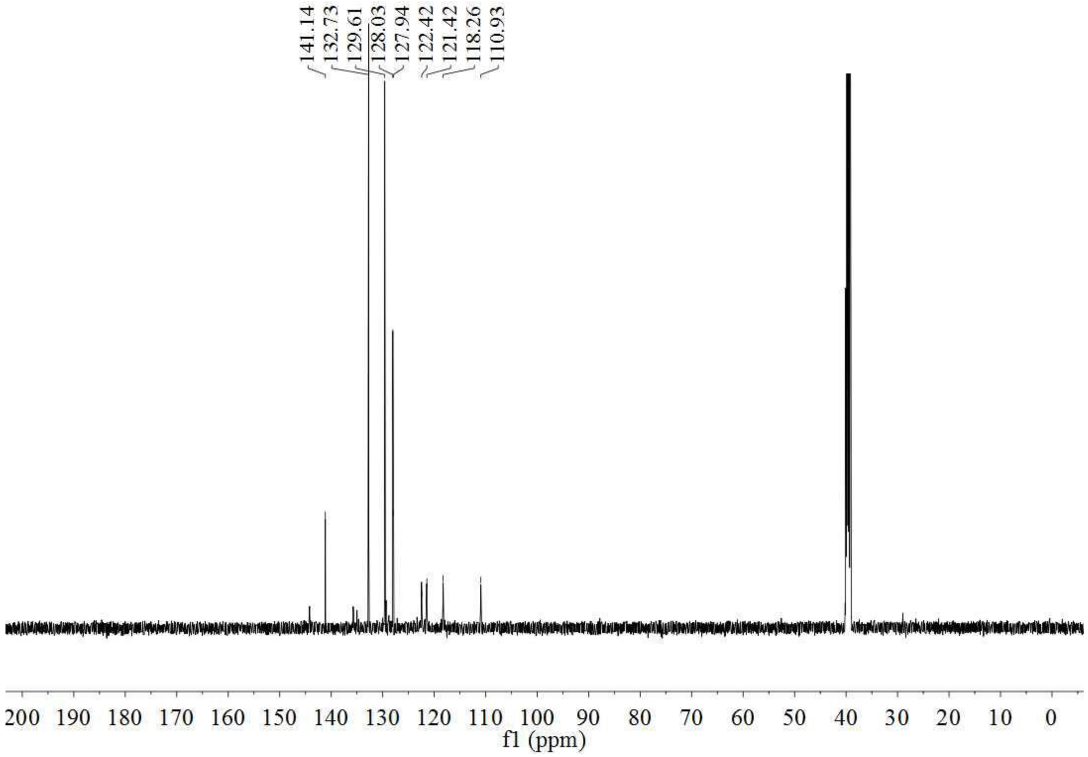

Figure 48. **9a** ^{13}C NMR

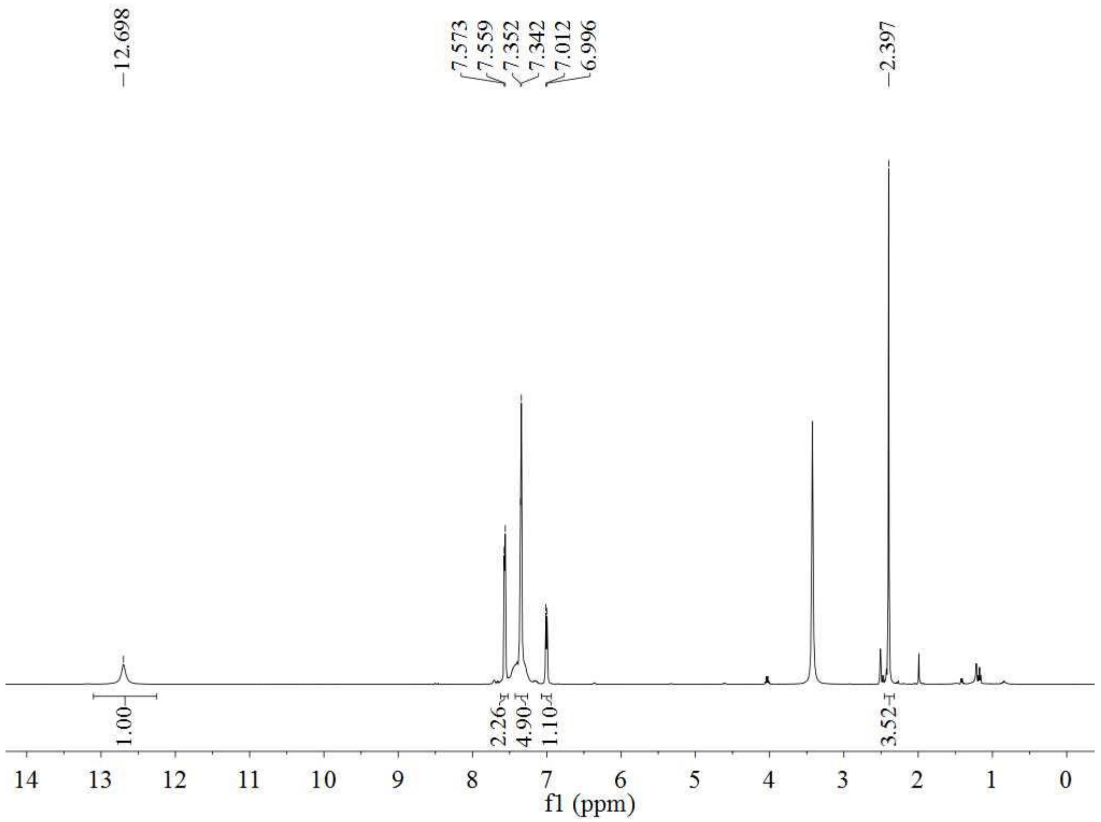

Figure 49. **9b** ^{1}H NMR

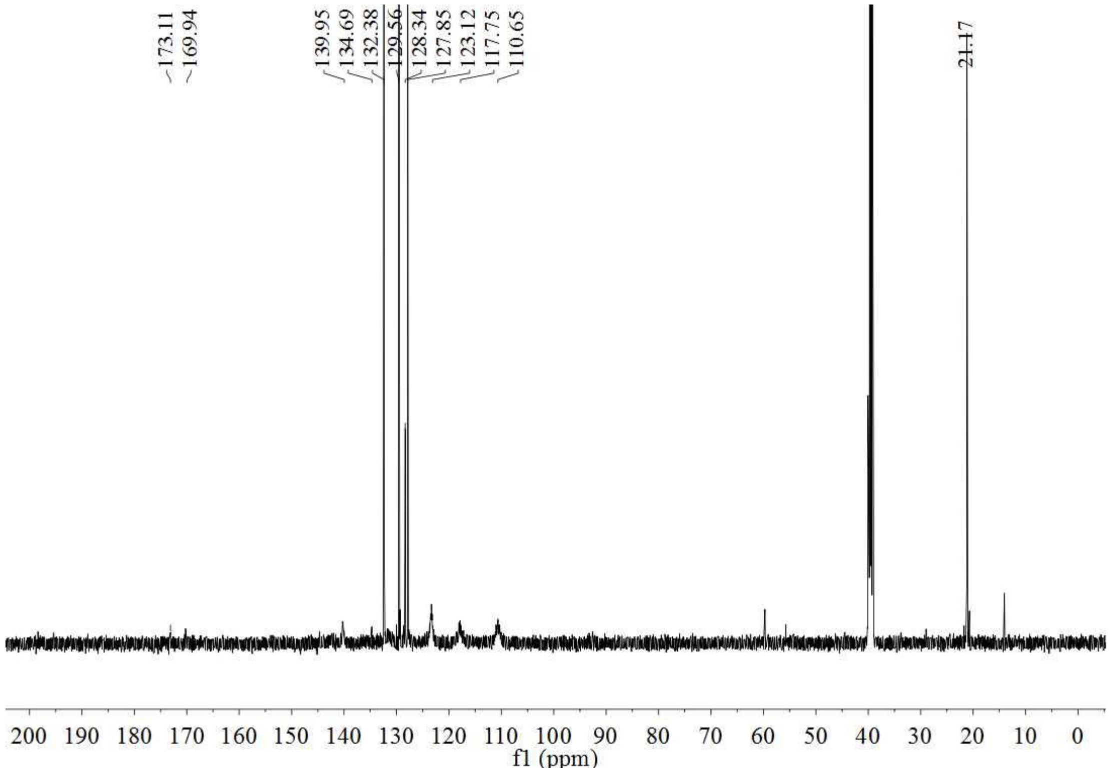

Figure 50. **9b** ^{13}C NMR

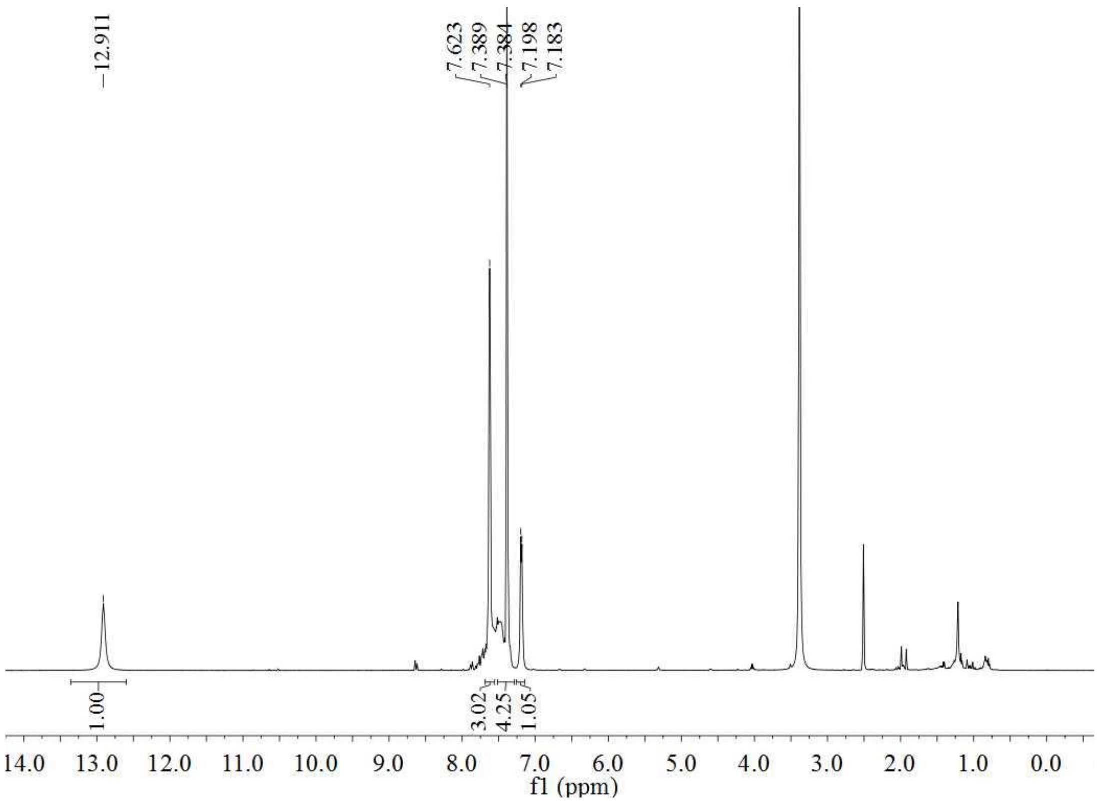

Figure 51. **9c** ^{1}H NMR

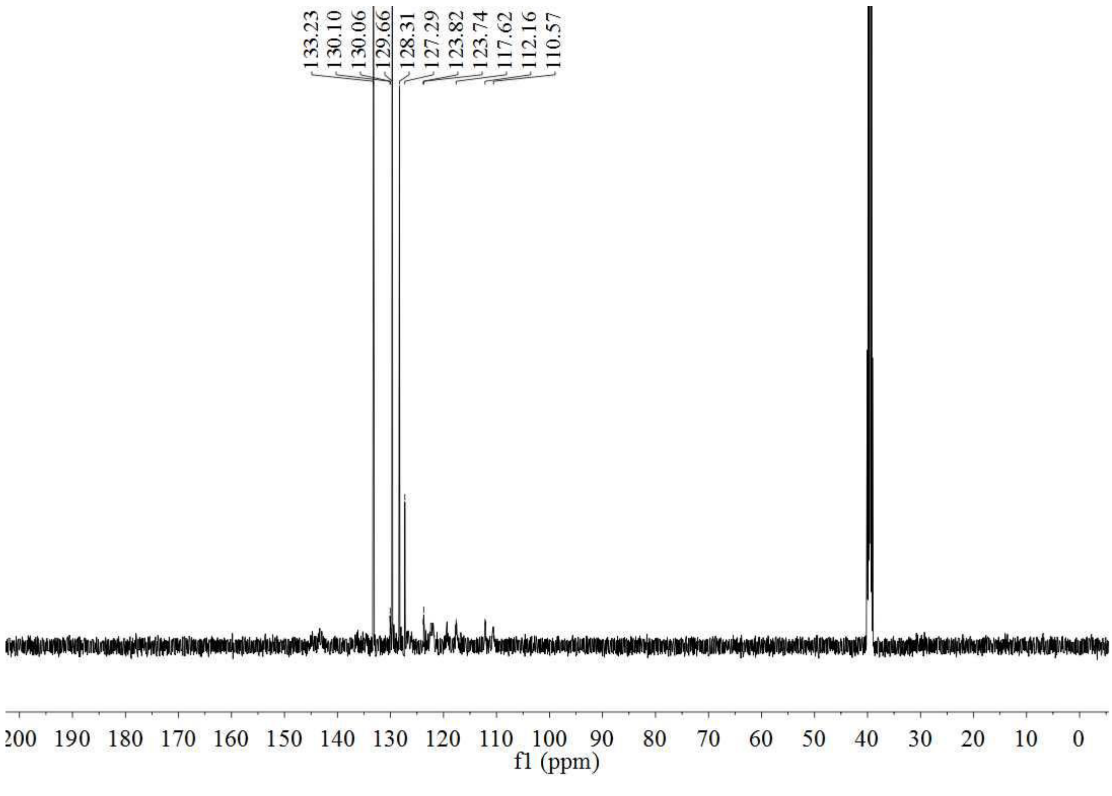

Figure 52. **9c** ^{13}C NMR

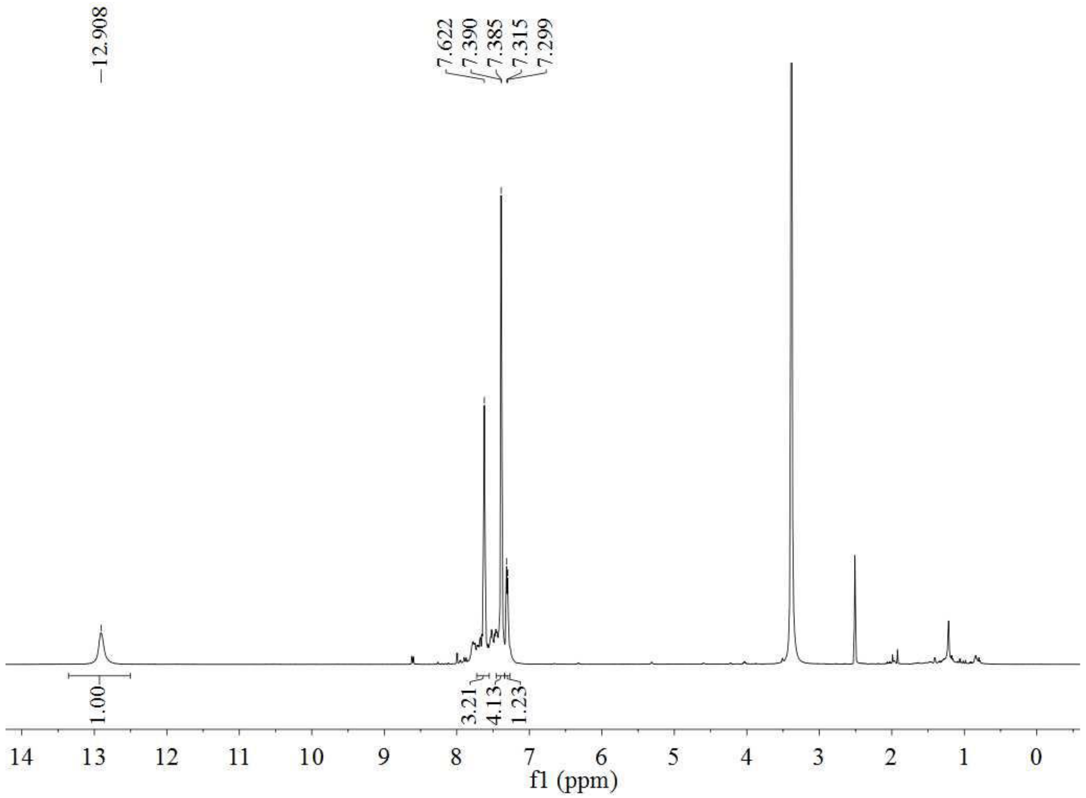

Figure 53. **9d** ^{1}H NMR

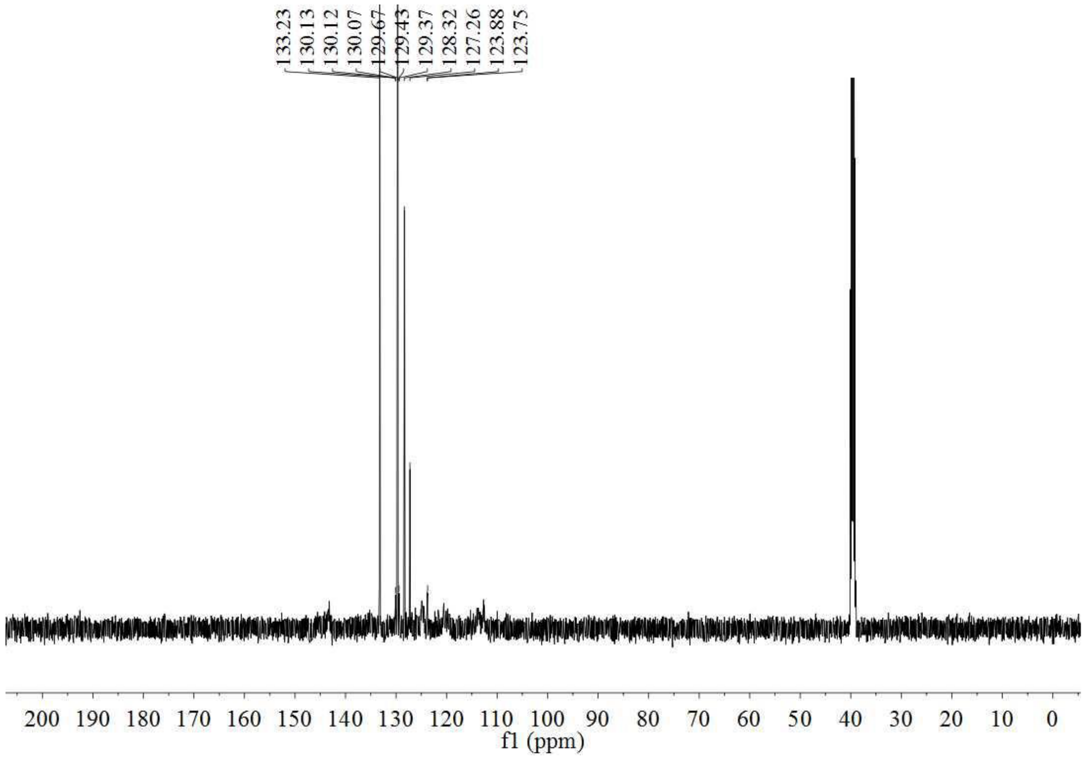

Figure 54. **9d** ^{13}C NMR

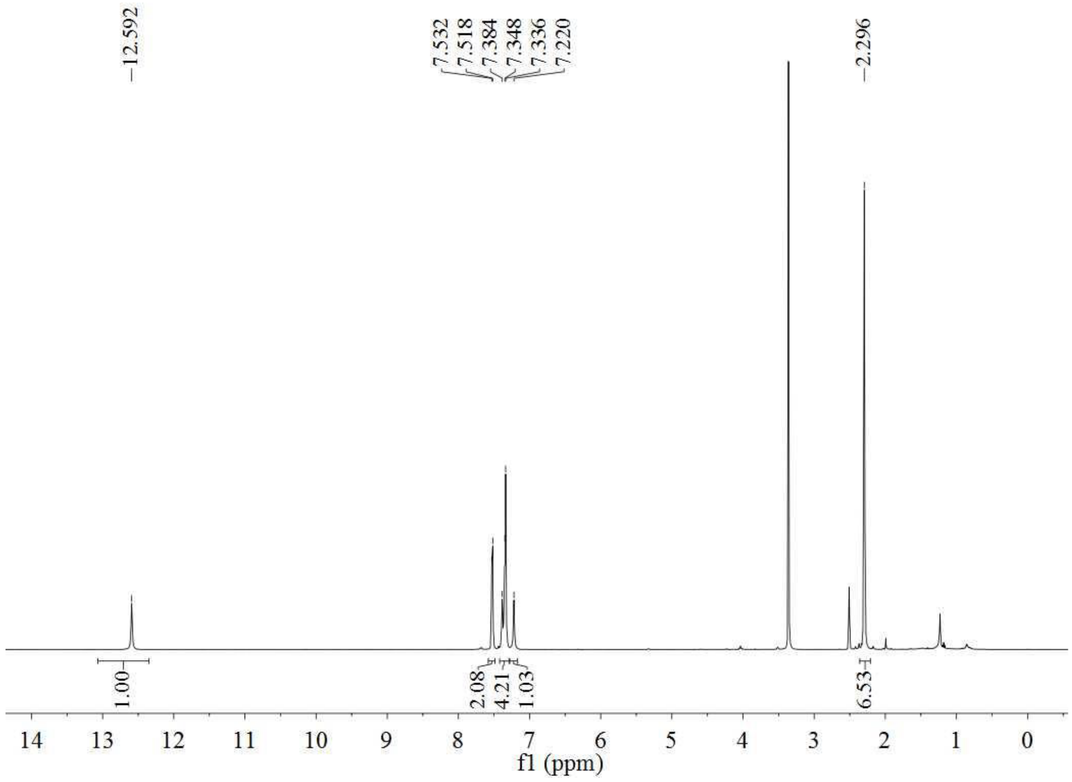

Figure 55. **9e** ^{1}H NMR

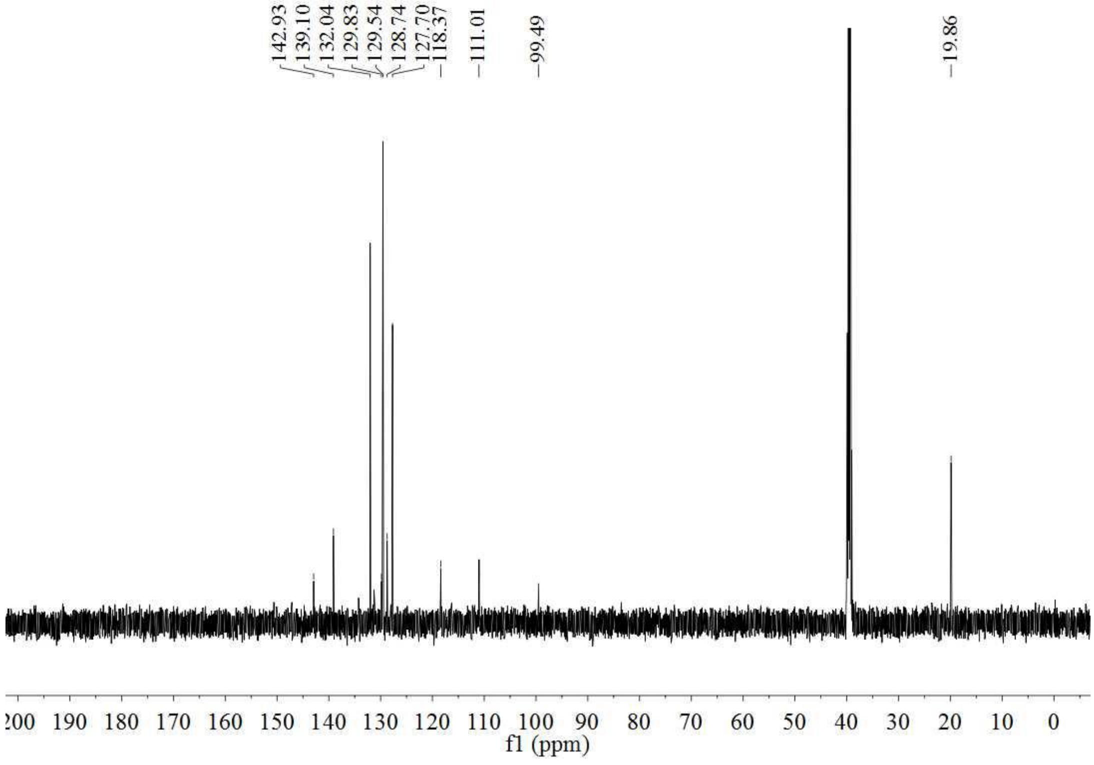

Figure 56. **9e** ^{13}C NMR
